# Maternal Circulatory NAD Precursor Levels and the Yolk Sac Determine NAD Deficiency‐Driven Congenital Malformation Risk

**DOI:** 10.1096/fj.202500708RR

**Published:** 2025-07-21

**Authors:** Kayleigh Bozon, Hartmut Cuny, Delicia Z. Sheng, Alena Sipka, Antonia W. Shand, Natasha Nassar, Sally L. Dunwoodie

**Affiliations:** ^1^ Victor Chang Cardiac Research Institute Sydney New South Wales Australia; ^2^ University of New South Wales Sydney New South Wales Australia; ^3^ School of Clinical Medicine, Faculty of Medicine and Health University of New South Wales Sydney New South Wales Australia; ^4^ Children's Hospital at Westmead Clinical School University of Sydney Sydney New South Wales Australia; ^5^ Department of Maternal Fetal Medicine Royal Hospital for Women Sydney New South Wales Australia; ^6^ Menzies Centre for Health Policy and Economics, Faculty of Medicine and Health University of Sydney Sydney New South Wales Australia

**Keywords:** congenital malformation, embryonic development, metabolism, NAD, pregnancy

## Abstract

Nicotinamide adenine dinucleotide (NAD) is an essential cofactor in hundreds of cellular processes. Genetic disruption of NAD *de novo* synthesis causes congenital NAD deficiency disorder (CNDD), characterized by multiple congenital malformations or death in utero. Patient outcomes are highly variable, likely due to differences in the availability of maternal NAD precursors vitamin B3 and tryptophan to the embryo and its extraembryonic tissues. Here, maternal plasma and yolk sac NAD metabolomes, embryonic NAD levels, and pregnancy outcomes were quantified in a CNDD mouse model to determine how maternal circulatory NAD precursor provision affects pregnancy outcome and to identify metabolic markers of CNDD risk. Maternal levels of nicotinamide positively correlated with embryonic NAD levels, highlighting its central role for embryonic NAD metabolism. Levels of nicotinamide‐derived excretion metabolites were the best predictors of adverse pregnancy outcome. NAD metabolomic analysis of pregnant women confirmed the relationship between dietary NAD precursor intake and circulatory nicotinamide and derived excretion product levels seen in mice, as women taking vitamin B3 supplements had elevated levels. Furthermore, mouse embryos with genetic disruption of NAD *de novo* synthesis (*Haao* ^−/−^) were more susceptible to CNDD when maternal circulatory nicotinamide was limited, as their yolk sacs cannot generate NAD *de novo* from tryptophan. Metabolites originating from *Haao* ^−/−^ embryos were detectable in maternal plasma, showing that embryonic NAD metabolism also affects maternal circulation. Together, our findings elucidate the complex interplay between NAD metabolism of mother and conceptus and identify metabolic markers in maternal circulation that predict risk of NAD deficiency‐related adverse pregnancy outcomes.

## Graphical abstract

Maternal circulatory L‐tryptophan (TRP) and nicotinamide (NAM) levels are proportional to dietary TRP intake. Yolk sacs, but not embryos, perform NAD *de novo* synthesis. This study explores the exchange of NAD precursors between mother and conceptus, and NAD generation in conceptal tissues via the NAD *de novo* synthesis (blue) or salvage (red) pathways according to *Haao* genotype. As *Haao* ^−/−^ conceptuses cannot perform NAD *de novo* synthesis, they are most susceptible to NAD deficiency. Created in BioRender. Bozon (2025) https://BioRender.com/pnnofdi

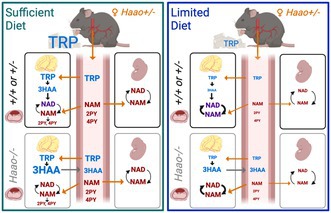

## 1 Introduction

Severe congenital malformations originate due to genetic predisposition, nongenetic factors, or combinations of gene–environment interactions [1, 2]. This complexity of risk factors makes determining the etiology difficult if not impossible, hindering diagnosis and prevention in subsequent pregnancies. Furthermore, multiple congenital malformations often co‐occur as syndromes or nonrandom malformation associations. Classification of such associations with unknown etiology is difficult because of the heterogeneity in malformation types and severity among affected individuals [3] and it has been suggested to collectively label them as “recurrent constellations of embryonic malformations” [4]. More research is needed to identify the factors modulating the variable expressivity of phenotypes and determining a mother's risk of having such adverse pregnancy outcomes.

**FIGURE 1:**
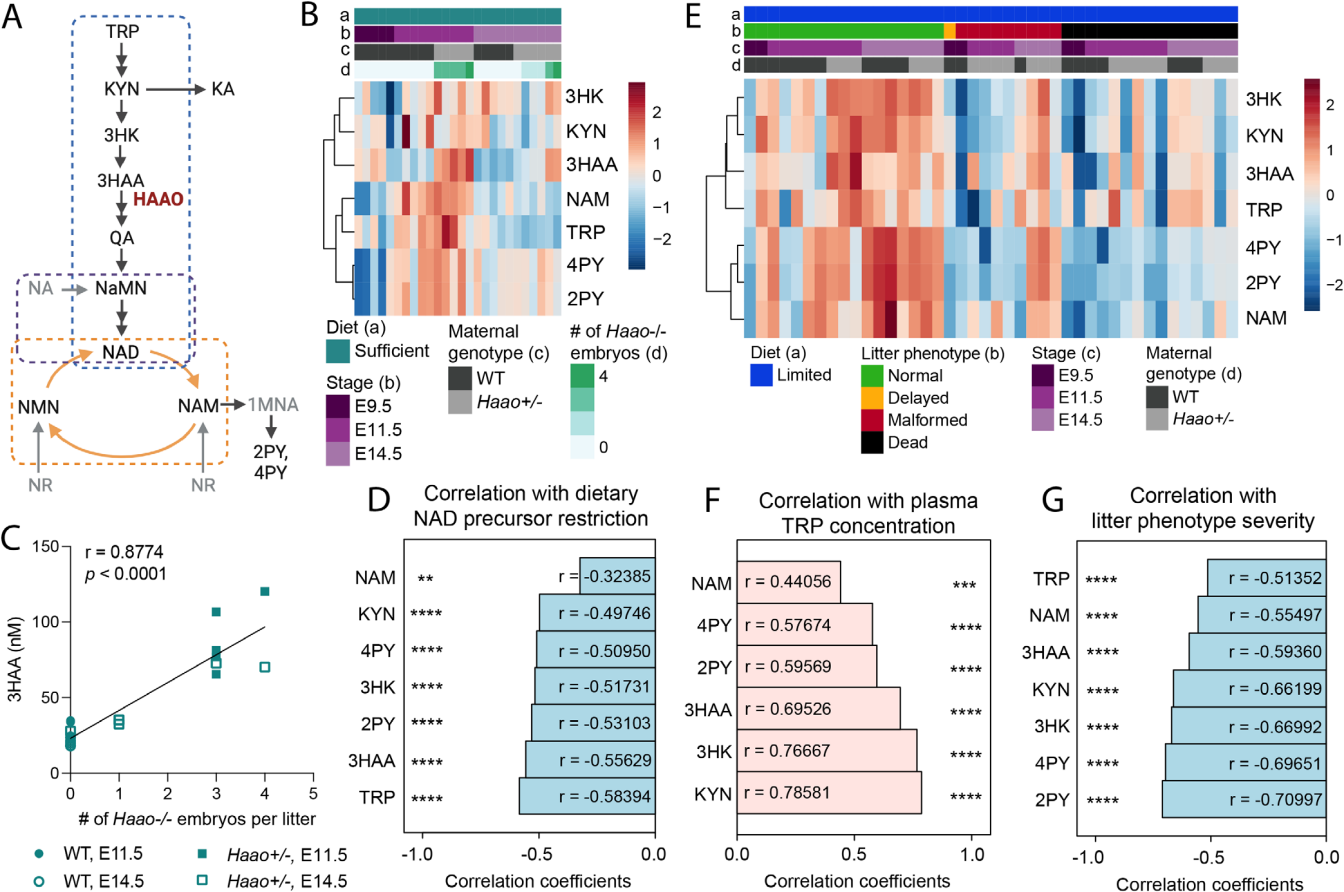
Maternal diet, maternal *Haao* genotype, and genotype of embryos affect the maternal plasma NAD metabolome. (A) Schematic overview of the NAD biosynthesis pathways. The NAD *de novo* Synthesis Pathway, Preiss‐Handler Pathway, and NAD Salvage Pathway are outlined by a blue, purple, and orange box, respectively. Metabolites quantified in this study (black), other relevant metabolites (gray), and HAAO enzyme position in the pathway (red) are shown. Created in BioRender. Bozon, K. (2025) https://BioRender.com/6t7mh24. (B) Heatmap comparing standardized concentrations of NAD‐related metabolites in the plasma of pregnant mice on Sufficient Diet (Table S1). Data are organized by diet (a), gestational stage (b), maternal *Haao* genotype (c), and number of *Haao* ^−/−^ embryos per litter (d). (C) Spearman's correlation (*r*) of maternal plasma 3HAA concentration with the number of *Haao* ^−/−^ embryos per litter at E11.5 and E14.5 from wild‐type and *Haao* ^+/−^ females provided the Sufficient Diet. (D) Pearson's correlation (*r*) of standardized maternal plasma metabolite levels with maternal dietary NAD precursor content (Sufficient and Limited Diet as categorial variables). Negative correlation indicates metabolite levels are lower in females on Limited Diet. (E) Heatmap comparing standardized levels of NAD‐related metabolites in the maternal plasma of females on Limited Diet. Data are organized by diet (a), litter phenotype (b), gestational stage (c), and maternal genotype (d). (F) Pearson's correlation (*r*) of standardized maternal plasma metabolite levels with plasma TRP concentration. (G) Pearson's correlation (*r*) of standardized maternal plasma metabolite levels with the phenotype of the mothers' respective litters. Litters were assigned phenotype categories ordered from normal to most severe (litter normal, litter included delayed embryos, litter included malformed embryos, litter dead). Negative correlation indicates that metabolite levels are highest in mothers with normal litters and lowest in mothers with dead litters. Panels (B) and (D–G) include data from maternal plasma collected at E9.5, E11.5, and E14.5; see Tables S8–S10 for sample size and numerical values. **q < 0.01, ***q < 0.001, ****q < 0.0001. WT, wild‐type; TRP, L‐tryptophan; KYN, kynurenine; KA, kynurenic acid; 3HK, 3‐hydroxykynurenine; 3HAA, 3‐hydroxyanthranilic acid; QA, quinolinic acid; NaMN, nicotinic acid mononucleotide; NA, nicotinic acid; NMN, nicotinamide mononucleotide; NAM, nicotinamide; NR, nicotinamide riboside; 1MNA, 1‐methylnicotinamide; 2PY, N‐methyl‐2‐pyridone‐5‐carboxamide; 4PY, N‐methyl‐4‐pyridone‐5‐carboxamide.

Congenital NAD deficiency disorder (CNDD) is an example of such recurrent malformations with variable expressivity, and miscarriages are also frequent among affected families [5]. CNDD is caused by biallelic loss‐of‐function variants in genes of the nicotinamide adenine dinucleotide (NAD) *de novo* Synthesis Pathway, a series of enzymatic steps by which L‐tryptophan (TRP) is metabolized to NAD [6]. In the mother, this conversion occurs in the liver [7] and in the conceptus (embryo and extraembryonic tissue) it occurs in the yolk sac and then in the embryonic liver once it has formed and becomes functional [8]. NAD is essential in hundreds of cellular processes [5]. Apart from TRP, NAD can be generated from the vitamin B3 vitamers nicotinic acid (NA) via the Preiss‐Handler Pathway and from nicotinamide (NAM) and nicotinamide riboside feeding into the NAD Salvage Pathway (from here onwards referred to as Salvage Pathway) [9, 10] (Figure 1A). All mammalian cells can generate NAD via the Salvage Pathway. TRP and NAM are the major NAD precursors present in the maternal circulation [7] and therefore accessible for the conceptus to generate NAD.

Human CNDD patients with biallelic loss‐of‐function variants in either *HAAO, KYNU*, *or NADSYN1* (genes of the NAD *de novo* Synthesis Pathway encoding 3‐hydroxyanthranilate 3,4‐dioxygenase, kynureninase, and NAD synthetase 1) have phenotypic variability even between individuals with the same genetic predisposition [6, 11, 12, 13, 14]. In mouse models, CNDD‐like multiple embryonic malformations can be induced by restricting the maternal NAD precursor provision [6, 13, 15], even in mice without genetic predisposition [15]. In those models, pregnancy outcomes vary widely between mice, from unaffected litters to those with malformed embryos and entire litters that died in utero, although the mice are genetically homogeneous and receive diets standardized in NAD precursor content [13, 15]. This indicates that the phenotypic severity of CNDD in mice and humans is modulated by unidentified nongenetic factors that influence the amount of NAD precursors available for the embryos to generate NAD.

Here, we address this in CNDD mouse models by quantifying the maternal plasma and yolk sac NAD metabolomes, the NAD status of embryos during and after organogenesis, and determining how these correlate to each other and to the pregnancy outcome.

## 2 Materials and Methods

### 2.1 Animal Experiments

All animal experiments were approved by the Garvan Institute of Medical Research/St Vincent's Animal Experimentation Ethics Committee, Sydney, Australia (approvals 18/27 and 21/18). All animal experiments were performed in accordance with the Animal Research: Reporting of In Vivo Experiments (ARRIVE) guidelines and the guidelines and regulations specified in the animal ethics approval. The animal experiments include dietary and genotype control groups. Numbers of mice used per experiment are indicated in the respective figures or Tables S1–S18. No experimental mice were excluded. The *Haao* loss‐of‐function mouse line (allele Haao^em1Dunw^, RRID:MGI:6285800) has been described previously [6].

Female C57BL/6J wild‐type (WT) (RRID:MGI:3028467) and *Haao* ^+/−^ mice to be used in timed matings were fed a Standard Diet with defined NAD precursor content (Specialty Feeds, Yanderra, NSW, Australia; Cat. #: SF22‐100) for at least 3 weeks prior to mating between 58 and120 days of age, as described previously [13, 15]. WT females were mated with WT males, and *Haao* ^+/−^ females were mated with *Haao* ^+/−^ males. From the start of pregnancy, confirmed by the presence of a vaginal copulation plug in the morning (defined as timepoint embryonic day [E]0.5), the feed was replaced with either Sufficient Diet, which is feed depleted in vitamin B3 and containing TRP as NAD precursor (Specialty Feeds; Cat. #: SF16‐049), representing the control group, or Limited Diet, which is feed depleted in both vitamin B3 and TRP (Specialty Feeds Cat. #: SF16‐097) and drinking water supplemented with 600 mg/L TRP, as described previously [15]. The diets and their NAD precursor compositions are summarized in Table S1.

Pregnant females were maintained on their respective diets until E9.5, E11.5, or E14.5, after which they were euthanized by CO_2_ asphyxiation followed by cervical dislocation. Maternal plasma was collected as described previously [16]. At E9.5, yolk sacs of whole litters of WT mothers were collected and pooled due to their small size. No yolk sacs were collected from litters of *Haao* ^+/−^ mothers at E9.5. Embryos were collected and assessed for gross external malformations such as exencephaly and for developmental delay, with embryos morphologically resembling E8.5 or earlier classified as delayed. At E11.5, yolk sacs were collected individually and from litters of both WT and *Haao* ^+/−^ mothers. E11.5 embryos were weighed at collection and phenotyped for external malformations. For an overview of assessed structures, see Table S3. At E14.5, embryos were weighed, phenotyped for external malformations, and their kidneys were dissected to measure the kidney length (to determine kidney hypoplasia as a CNDD‐related anomaly). For an overview of assessed structures, see Table S4. The embryonic liver was dissected as well. All tissue samples were snap frozen for storage until metabolomic analysis. All plasma and tissue samples for enzymatic and metabolic analysis were measured in randomized batches with the experimenters blinded to the genotype and diet conditions of the samples.

Amnion samples of each conceptus were genotyped for the *Haao* loss‐of‐functional allele as described previously [6].

### 2.2 Metabolite Quantification by UHPLC–MS/MS

NAD‐related metabolites TRP, kynurenine (KYN), 3‐hydroxykynurenine (3HK), 3‐hydroxyanthranilic acid (3HAA), NAD^+^, NAM, N‐methyl‐2‐pyridone‐5‐carboxamide (2PY), and N‐methyl‐4‐pyridone‐5‐carboxamide (4PY) were quantified in plasma samples of pregnant mice collected at dissection using ultra‐high performance liquid chromatography–tandem mass spectrometry (UHPLC‐MS/MS) as described previously [16]. The same metabolites plus nicotinic acid mononucleotide (NAMN) and nicotinamide mononucleotide (NMN) were measured in E9.5 and E11.5 yolk sacs by UHPLC–MS/MS and normalized to protein concentration as described previously [8], except that the methanol used to wash the pellets contained 0.75% β‐mercaptoethanol.

### 2.3 NAD Quantification by Enzymatic Cycling Assay

Total NAD(H) levels (NAD^+^ and NADH) of E11.5 whole embryos were measured with an enzymatic cycling assay as described previously [15]. E9.5 whole embryos and E14.5 partial embryos (details see below) were measured by the same assay, with the protocol previously used for adult liver tissue [15]. Due to their larger size and high NAD(H) content, E14.5 embryos were dissected to remove the kidneys and livers, frozen, and ground into powders with a mortar and pestle. The frozen powders were mixed well, weighed, and lysis buffer was added to 25 mg of embryo powder for NAD(H) quantification. NAD(H) concentration was normalized to protein concentration determined in the lysate by Pierce BCA Protein Assay Kit (Thermo Fisher Scientific (RRID:SCR_008452); Cat. #: 23225).

### 2.4 HAAO Enzyme Assay

HAAO enzyme activity assays were performed on E11.5 yolk sacs as described previously [8]. Yolk sacs were analyzed individually, with either 6 or 9 μL of tissue lysis buffer (10 mM protease inhibitor in PBS) added per mg of yolk sac tissue to obtain a total volume of ≥ 45 μL. Protein concentration was determined by BCA protein assay.

### 2.5 Human Clinical Study

One hundred twelve pregnant women between 20 and 40 years of age were recruited at the Royal Hospital for Women, Randwick, Australia for a clinical study. Ethics approval has been obtained from the South‐Eastern Sydney Local Health District Human Research Ethics Committee (Approval number 2021_ETH11117). All methods were performed in accordance with the relevant guidelines and regulations. Written informed consent was received from all participants. Participants completed a questionnaire to assess their vitamin B3 supplementation status during pregnancy from multivitamins, B vitamin complex, pure vitamin B3, and/or other vitamin B3‐containing products. The daily vitamin B3 intake from supplements (on top of the vitamin B3 intake from the diet, which was not assessed) was then calculated based on product specifics and dosage.

From each participant, a fasting morning blood sample was collected between 10 and 24 weeks of pregnancy. Blood collection, sample processing to obtain plasma, metabolite extraction from the plasma, and quantification of NAD‐related metabolites via UHPLC–MS/MS was done as described [13, 16].

### 2.6 Statistical Analysis

MetaboAnalyst 6.0 [17] (RRID:SCR_015539) was used for the analysis of all plasma and yolk sac NAD metabolome data except for Spearman's correlation analyses and one‐way ANOVA, for which GraphPad Prism (version 10; RRID:SCR_002798) was used. To allow comparisons across metabolites with different concentration ranges in MetaboAnalyst, measured concentrations were standardized by log10 transformation and Pareto scaling. Standardized concentration data was used to generate heatmaps, principal component analysis (PCA) plots, Pearson's correlation analyses, and two‐way ANOVA. Two‐way ANOVA was used to compare the effect of embryo genotype and phenotype on E11.5 yolk sac data. Spearman's correlation analysis was used to test the correlation of nonstandardized NAD‐related metabolite levels within and between tissues or metabolite levels with embryo NAD concentration or the number of *Haao* ^−/−^ embryos in the litter. One‐way ANOVA with Tukey's multiple comparisons test was used to compare embryo NAD concentrations between diet groups. Kruskal–Wallis test was used to test litter‐to‐litter variability in NAD enzyme assay data. Kruskal–Wallis with Dunn's multiple comparisons test was used to test the effect of diet on HAAO enzyme activity in E11.5 yolk sacs. Unpaired two‐tailed t‐test was used to compare plasma concentrations of NAM, 2PY, and 4PY between pregnant women who took ≥ 18 mg/d vitamin B3 during pregnancy and those that did not take vitamin B3 supplements.

## 3 Results

### 3.1 Embryo Phenotype Varies Between Litters Despite Provision of a Defined NAD Precursor Restricted Diet During Gestation

To better understand the extent and features of the phenotypic variability of CNDD patients and corresponding mouse models, we used two different diets and assessed pregnant mice at three times of gestation (E9.5, E11.5, and E14.5). The two diets provided during pregnancy, based on a previous study [15], are depleted in vitamin B3 and either contain sufficient TRP to generate NAD via the NAD *de novo* Synthesis Pathway (Sufficient Diet) or are restricted in TRP (Limited Diet; see Table S1 for diet specifics). Limited Diet was empirically established to be at the NAD precursor threshold that causes CNDD in mice [15]. *Haao* is a nonredundant gene of NAD *de novo* synthesis (Figure 1A). To identify how embryonic *Haao* genotype influences the phenotypic variability between litters, *Haao* ^+/−^ females were mated with *Haao* ^+/−^ males to generate offspring of three *Haao* genotypes (*Haao* ^+/+^, *Haao* ^+/−^, and *Haao* ^−/−^). These were also compared to offspring of wild‐type (WT) intercrosses.

When Limited Diet was provided during gestation, variable pregnancy outcomes occurred, ranging from phenotypically normal litters to litters with embryos exhibiting CNDD‐related congenital malformations or entire litters that died in utero (Supporting Information: Results, Figure S1, Tables S2–S7). This was irrespective of whether the mothers and embryos were WT or carried *Haao* loss‐of‐function alleles, similar to previous observations [15], indicating a genotype‐independent effect.

### 3.2 NAD Related Metabolite Levels in Maternal Plasma Are Affected by Diet and Correlate With Pregnancy Outcome

To establish the relationship between maternal provision of NAD precursors, pregnancy outcome, and phenotypic litter‐to‐litter variability, we measured the maternal circulatory NAD metabolome in plasma samples collected from pregnant females at E9.5, E11.5, and E14.5 by UHPLC–MS/MS [16] (Tables S8–S10). The chosen stages span the period of organogenesis and represent the stages when embryos develop malformations or die.

We first assessed samples from mice provided with the Sufficient Diet to determine how the maternal circulatory NAD metabolome changes during pregnancy. Levels of TRP and NAM, the two most abundant NAD precursors in the murine circulation [7, 16], increased from E9.5 to E11.5, then declined at E14.5 (Figure 1B, Figure S2B,C). The two main degradation products of NAM, 2PY and 4PY, showed the same trend (Figure 1B, Figure S2D,E). Furthermore, pregnant *Haao* ^+/−^ mice had elevated plasma concentrations of 3HAA, the substrate of HAAO enzymatic activity, relative to their WT counterparts at E11.5 and E14.5 (Figure 1B). To determine whether this elevated 3HAA originated from the lack of one functional *Haao* allele in the mother or from *Haao* ^−/−^ embryos in the litter, which are unable to metabolize 3HAA, we correlated the maternal 3HAA levels to the number of *Haao* ^−/−^ embryos in the litter. There was a significant positive correlation between the two (Figure 1C), showing that *Haao* ^−/−^ embryos influence maternal circulatory metabolite levels.

Next, we determined how reducing the provision of the NAD precursor TRP, as with Limited Diet, affects the maternal circulatory NAD metabolome and whether variability in metabolite levels correlates with the observed phenotypic variability between litters. With Limited Diet, levels of NAM, 2PY, and 4PY changed dynamically between E9.5, E11.5, and E14.5, but unlike with Sufficient Diet, they were highest at E14.5 relative to the earlier time points (Figure S2C–E). Overall, Limited Diet provision resulted in lower plasma TRP and NAM levels relative to Sufficient Diet, and all other measured metabolites followed the same trend (Figure 1D, Figure S2A). This indicates that the difference in TRP content of the diets drives metabolic changes (Figure 1F) and confirmed that the amount of NAD precursors provided in the diet is reflected in the levels of the major NAD precursor metabolites in the plasma (Figure 1D). In addition, metabolite levels correlated with the severity of pregnancy outcomes, with the strongest correlation seen with the Salvage Pathway excretion products 2PY and 4PY (Figure 1E,G, Figure S2A,D,E). Furthermore, the maternal levels of 3HAA significantly correlated with the number of *Haao* ^−/−^ embryos in the litter (Figure S2F), as observed with Sufficient Diet.

Taken together, these data show that the maternal circulatory NAD metabolome is influenced by the diet and *Haao* genotypes of mother and embryos. In addition to this dietary effect, several metabolites of the NAD *de novo* and Salvage Pathway were less abundant in mothers with adverse pregnancy outcomes compared to those with phenotypically normal litters, with the strongest correlation seen with the Salvage Pathway excretion products 2PY and 4PY. This indicates that the NAD precursor levels in the circulation dictate phenotypic outcome and that variability in phenotypic severity between litters is driven by differences in this circulatory NAD precursor availability.

### 3.3 Embryo NAD Levels Are Dynamic During Organogenesis

**FIGURE 2:**
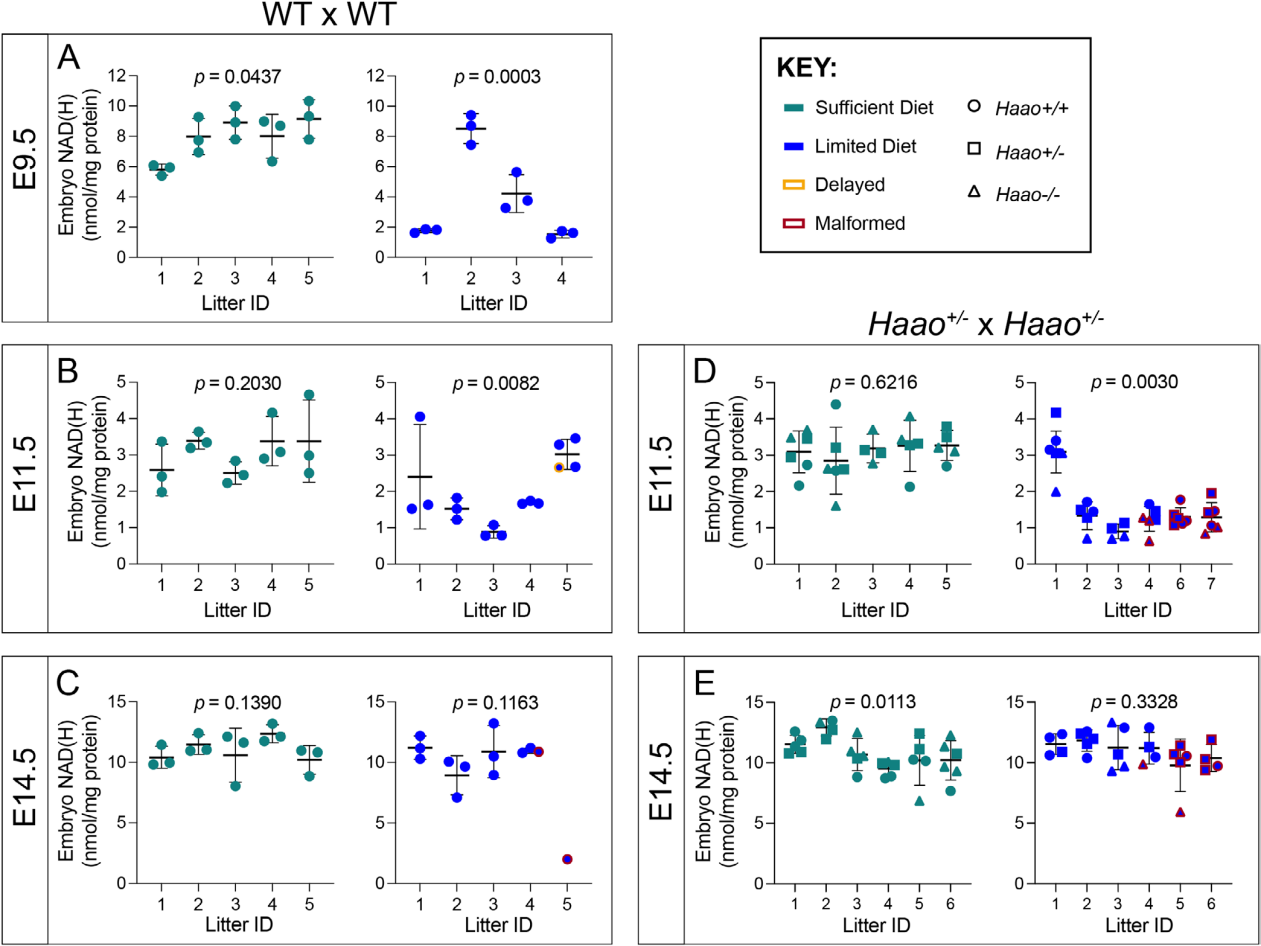
Maternal provision of Limited Diet results in varied embryo NAD(H) levels between litters at E9.5 and E11.5, reflecting the observed variability in pregnancy outcomes. (A–C) Concentrations of NAD(H) relative to protein content in E9.5 (A), E11.5 (B), or E14.5 (C) embryos from pregnant WT mice mated to WT males and provided the Sufficient Diet (teal) or Limited Diet (blue). (D), (E) NAD(H) concentrations in E11.5 (D) or E14.5 (E) embryos from *Haao* ^+/−^ × *Haao* ^+/−^ matings. Embryos delayed or malformed at the time of dissection are indicated by yellow or red outline, respectively. For details of the malformations observed, see Tables S3 and S4. Dots represent NAD(H) levels and bars indicate the mean ± standard deviation per litter. One to six embryos were measured per litter (see Figure S1 for total litter sizes). The *p* values represent Kruskal–Wallis test results comparing NAD levels across litters. WT, wild‐type; NAD(H), sum of NAD^+^ and NADH.

Previous data suggest that the period of embryonic NAD deficiency varied and that its extent and timing differ between litters, resulting in variable CNDD phenotypes [8, 18]. To understand how NAD levels compare with phenotypes within and between litters, we measured total NAD(H) levels (NAD^+^ and NADH) in embryos of corresponding litters at E9.5, E11.5, and E14.5 (Tables S11–S14). As observed previously [15], Limited Diet caused a significant overall decrease in NAD(H) levels in embryos from the WT × WT matings at both E9.5 and E11.5. Besides this general trend, embryo NAD(H) levels varied extensively between litters (Figure 2A,B). This variability diminished by E14.5 (Figure 2C). The same trends were observed for *Haao* ^+/−^ mothers on the Limited Diet (Figure 2D,E) and there were no differences in NAD(H) levels between embryonic *Haao* genotypes.

Not all embryos with the lowest NAD(H) levels at E11.5 had visible external malformations (Figure 2, Table S6), suggesting that these embryos either had undetectable internal malformations or had not been NAD‐deficient at critical earlier stages in organogenesis. Embryo death was prevalent at all three stages (Table S2), indicating NAD deficiency occurred prior to the time of sampling in the respective litters. The incidence of embryo death was higher at E11.5 and E14.5 compared to E9.5, indicating that a proportion of embryos die between E9.5 and E11.5. Taken together, we found inter‐litter variability and limited correlation between embryo NAD(H) levels and phenotype at E9.5 to E14.5, confirming that embryonic NAD deficiency is transient and variable in timing.

### 3.4 Embryo NAD Levels Can Be Predicted by Maternal Circulatory Metabolites

To generate NAD, embryos are dependent on maternally provided NAD precursors (largely TRP and NAM [7]) which are metabolized by the yolk sac or the embryo [8]. *Haao* ^−/−^ embryos cannot catabolize TRP to NAD and are dependent on NAM provision. Thus, the type and levels of maternal NAD precursors should determine embryo NAD levels.

**FIGURE 3:**
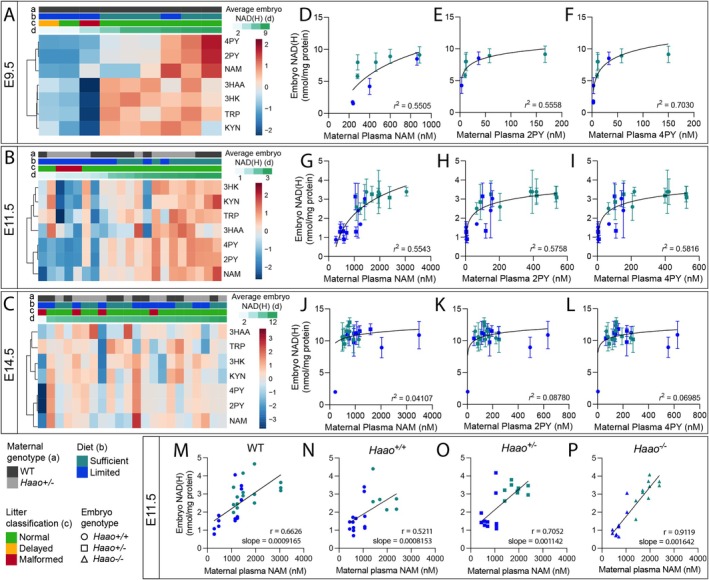
Maternal circulatory NAM and Salvage Pathway excretion products correlate with embryo NAD levels. (A–C) Heatmaps showing standardized levels of NAD‐related metabolites in the plasma of female pregnant WT and *Haao* ^+/−^ mice at E9.5 (A), E11.5 (B), and E14.5 (C). Data are organized by maternal *Haao* genotype (a), diet (b), litter phenotype (c), and average embryo NAD(H) concentration of the litter (nmol/mg protein) (d). See Tables S8–S10 for sample size and numerical values. (D–L) Correlation between embryo NAD(H) concentration and maternal plasma NAM, 2PY, and 4PY concentration at E9.5 (D–F), E115, (G–I), and E14.5 (J–L). Samples from both diets and all *Haao* genotypes are represented. Dots represent average embryo NAD levels of the respective litter and bars indicate the standard deviation. Semilogarithmic curves (X is log, Y is linear) and their goodness of fit (*r* ^2^) are included. (M–P): Correlation between embryo NAD(H) concentration and maternal plasma NAM at E11.5 from the Sufficient and Limited Diet conditions. (M) WT embryos from WT mothers, (N–P) embryos of *Haao* ^+/−^ × *Haao* ^+/−^ matings, separated by *Haao* genotype. Spearman's correlation (*r*) and slope of a linear fit are indicated for each graph. WT, wild‐type; NAD(H), sum of NAD^+^ and NADH; TRP, L‐tryptophan; KYN, kynurenine; 3HK, 3‐hydroxykynurenine; 3HAA, 3‐hydroxyanthranilic acid; NAM, nicotinamide; 2PY, N‐methyl‐2‐pyridone‐5‐carboxamide; 4PY, N‐methyl‐4‐pyridone‐5‐carboxamide.

To understand how NAD precursor levels in the maternal circulation relate to the NAD levels of their embryos, embryo NAD(H) levels were averaged per litter and compared with maternal plasma NAD metabolomes at E9.5, E11.5, and E14.5. This showed that the maternal plasma metabolites NAM, 2PY, and 4PY positively correlated with embryo NAD(H) levels at E9.5 and E11.5 (Figure 3A,B,D–I). At E14.5, embryo NAD(H) levels were more consistent overall and did not differ between Sufficient and Limited Diet (Figure 3C,J–L).

**FIGURE 4:**
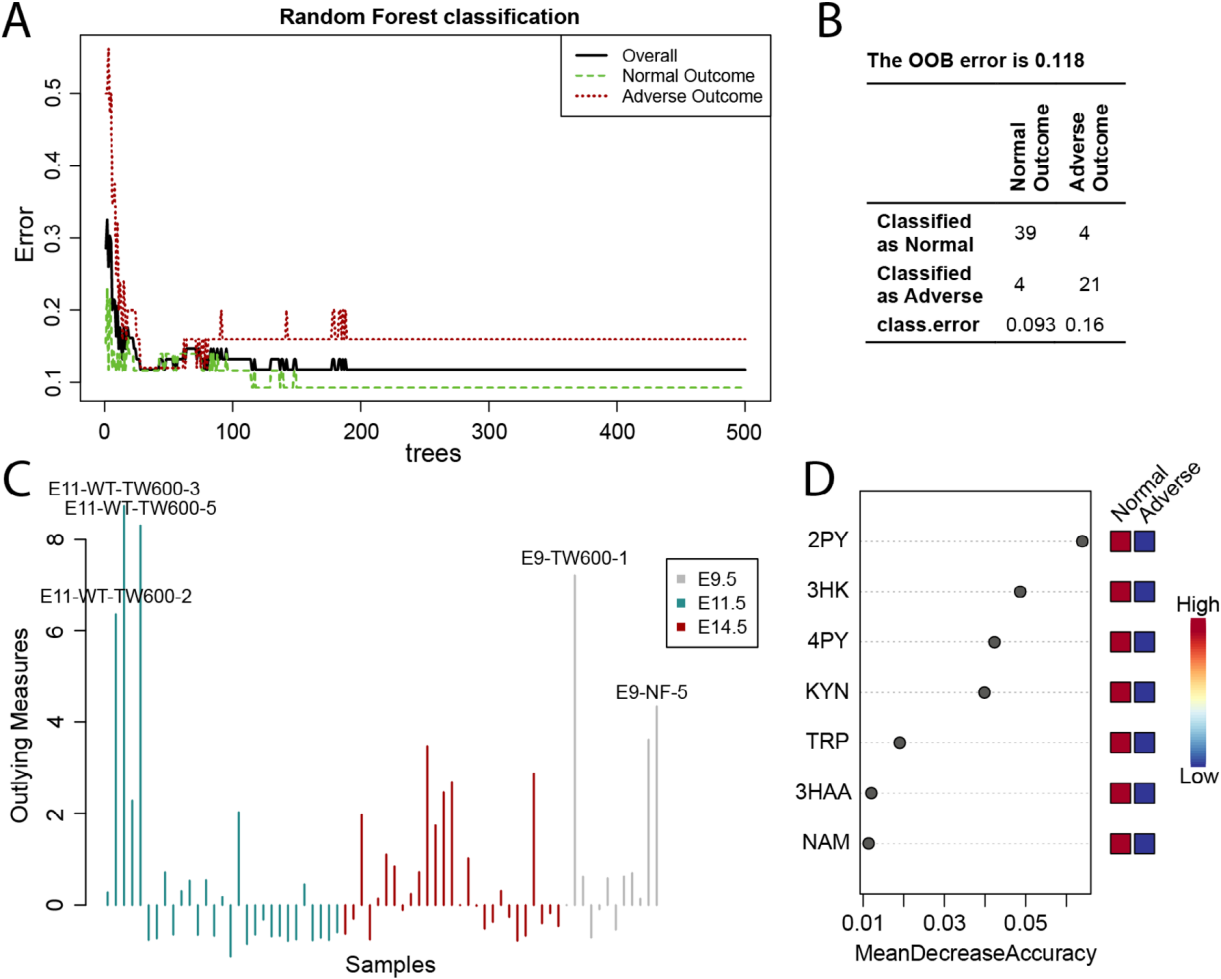
Random Forest supervised machine learning algorithm to predict adverse pregnancy outcome from metabolite levels in the maternal circulation. (A–D) Cumulative maternal plasma NAD metabolome data, collected at three different stages of pregnancy (E9.5, E11.5 or E14.5) with two different diets (Sufficient and Limited Diet), analyzed using a Random Forest algorithm (MetaboAnalyst v6.0). Maternal plasma concentrations of the included seven metabolites were standardized (log10 transformed and Pareto scaled) for analysis. (A) Cumulative classification error rates. Error rates are shown for Overall (black), Normal Outcome (no dead or malformed embryos in the litter, green) and Adverse Outcome (litter had malformed or dead embryos, red). (B) Classification performance. The out‐of‐bag (OOB) error and classification errors (class. error) are indicated. (C) Outlying measures analysis with the top five potential outliers indicated. (D) The seven metabolites, ranked by their contributions to classification accuracy. See Tables S8–S10 for sample size and numerical values. TRP, L‐tryptophan; KYN, kynurenine; 3HK, 3‐hydroxykynurenine; 3HAA, 3‐hydroxyanthranilic acid; NAM, nicotinamide; 2PY, N‐methyl‐2‐pyridone‐5‐carboxamide; 4PY, N‐methyl‐4‐pyridone‐5‐carboxamide.

The correlation between embryo NAD and maternal circulatory NAM and its derivatives 2PY and 4PY indicates that embryo NAD levels can be inferred from these metabolites. Similarly, 2PY and 4PY had the strongest correlation with pregnancy outcome (Figure 1G). To test whether these represent potential biomarkers to predict pregnancy outcome, we used a decision tree‐based statistical machine learning approach, Random Forest, to classify litters into either normal or adverse pregnancy outcomes. Performed on the whole set of maternal NAD metabolome data, Random Forest classified litters relatively accurately with an overall out‐of‐bag error estimate of 0.118 (Figure 4). Furthermore, 2PY and 4PY were two of the three most important variables to the Random Forest decision tree (Figure 4D). Those litters misclassified by the algorithm as malformed were collected at E9.5 or E11.5, where phenotypic analysis was limited to externally visible malformation types. Therefore, these litters either had existing internal defects or would have developed malformations later in gestation.

In summary, embryo NAD levels strongly correlate with maternal provision of NAM, suggesting that Salvage Pathway activity using maternal NAM is the embryo's primary mode of generating NAD during organogenesis. As the amount of the Salvage Pathway waste products 2PY and 4PY reflect NAM availability [13, 18, 19, 20], these represent promising potential biomarkers for predicting embryo NAD levels, and therefore CNDD risk to be used in human patients.

### 3.5 Vitamin B3 Supplementation Increases Circulatory Levels of NAM, 2PY, and 4PY in Pregnant Women

To assess whether these findings in mice may translate to human pregnancy, we determined how vitamin B3 supplementation during pregnancy influences the NAD metabolome in 112 first or second‐trimester pregnant women (average: 15.3 ± 3.3 weeks, range: 10.4–24.1 weeks gestation). Of the study cohort, 40 participants had not taken vitamin B3 supplements (‐B3 group) and 72 participants had taken 18–68 mg/d of vitamin B3 during their pregnancy (+B3 group).

**FIGURE 5:**
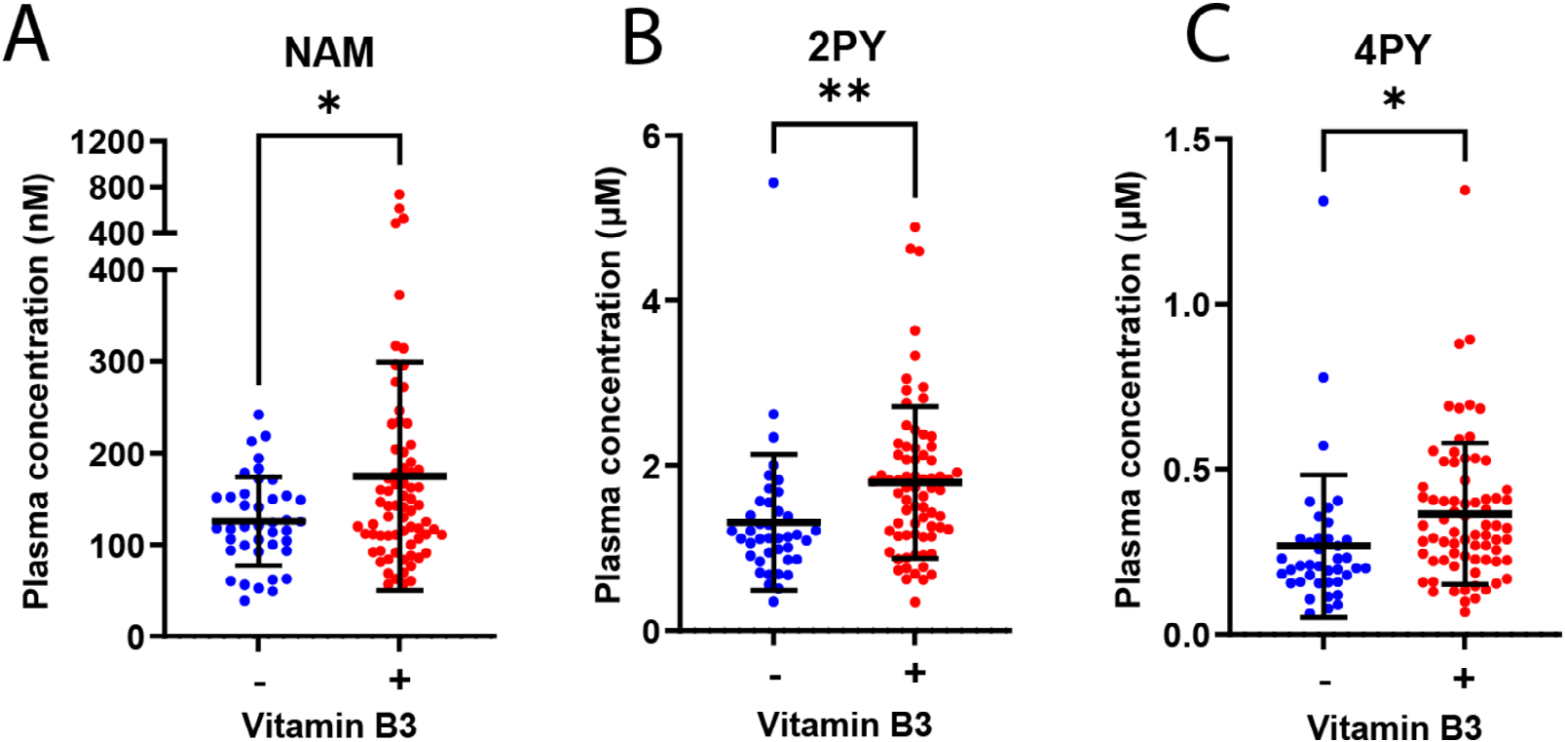
Dietary supplementation with 18–68 mg/d of vitamin B3 affects maternal plasma levels of NAM and Salvage Pathway excretion products in pregnant women. (A–C) Plasma concentrations of NAM (A), 2PY (B), and 4PY (C) of 112 pregnant women, separated into those that did not take vitamin B3‐containing dietary supplements (*n* = 40, blue dots) and those that took between 18 and 68 mg/d of vitamin B3 during pregnancy (*n* = 72, red dots). Bars indicate the mean and standard deviation. *p < 0.05, **p < 0.01 (unpaired two‐tailed t test). NAM, nicotinamide; 2PY, N‐methyl‐2‐pyridone‐5‐carboxamide; 4PY, N‐methyl‐4‐pyridone‐5‐carboxamide.

NAM, 2PY, and 4PY levels were significantly higher in the plasma of the +B3 group (Figure 5). This shows that the additional B3 had a direct effect on circulatory NAM and suggests that NAM levels were in excess as indicated by elevated levels of excretion metabolites 2PY and 4PY. This is in concordance with the effect of diets with different NAD precursor content in the mouse model on NAM and Salvage Pathway excretion metabolite levels (Figure 1D, Figure S2A–E). A difference between the models is that our Limited Diet represents an NAD precursor deficient diet, whereas the basal dietary intake of vitamin B3 in the studied cohort of pregnant women, not including TRP as NAD precursor or any additional B3 supplements, is estimated to be ~22 mg/day [21]. Given the recommended daily intake of vitamin B3 in human pregnancy is 18 mg/day [22], any B3 supplementation on top of the basal intake would represent an excess.

### 3.6 Embryos With Loss of NAD *de Novo* Synthesis Capability Are More Dependent on Maternal NAM Provision

Maternal circulatory levels of NAM and the excretion metabolites reflect the pregnancy outcome of entire litters. However, we observed additional nuances in embryo phenotypes within affected litters on the Limited Diet. *Haao* ^−/−^ embryos were more severely affected than their littermates because all *Haao* ^−/−^ embryos in affected litters were either malformed or absent (Tables S6 and S7). This is likely because their inability to perform *de novo* NAD synthesis makes them more reliant on maternal NAM provision. Although maternal plasma NAM positively correlated with embryo NAD levels for all embryo genotypes at E11.5 (Figure 3M–P), the correlation was strongest and the slope of the line of best fit steepest in *Haao* ^−/−^ embryos. This indicates that *Haao* ^−/−^ embryo NAD levels are more dependent on maternal NAM at critical stages of organogenesis (Figure 3P) than those of *Haao* ^+/+^ or *Haao* ^+/−^ embryos.

By E14.5, the correlation between maternal plasma NAM and embryo NAD was diminished or lost for all *Haao* genotypes. This is because both metabolites were less variable relative to E11.5, resulting in tighter clustering (Figure S3). This suggests that sufficient maternal NAM was available for all embryos at E14.5, which is supported by the lack of NAD‐deficient embryos on Limited Diet at E14.5 (Figure 2E).

### 3.7 Maternal Provision of NAD‐Related Metabolites Dictates Yolk Sac NAD *de Novo* Synthesis Activity at Mid‐Gestation

The inability to perform NAD *de novo* synthesis ultimately distinguishes *Haao* ^−/−^ embryos from *Haao* ^+/+^ and *Haao* ^+/−^ littermates and makes them likely to develop CNDD‐related defects. Further, maternal circulatory levels of TRP and the NAD *de novo* synthesis pathway intermediates 3HK and KYN are important variables to the Random Forest decision tree that accurately predicts pregnancy outcome (Figure 4), confirming NAD *de novo* synthesis activity in the conceptus contributes to phenotype. We recently showed that the yolk sac is the only site of NAD *de novo* synthesis activity in the conceptus from E10.5 until its function is established in the embryonic liver after E12.5 [8]. Here, we showed that NAD *de novo* synthesis activity already starts in the yolk sac from E9.5 (Supporting Information: Results, Table S15, Figure S4A–C).

**FIGURE 6:**
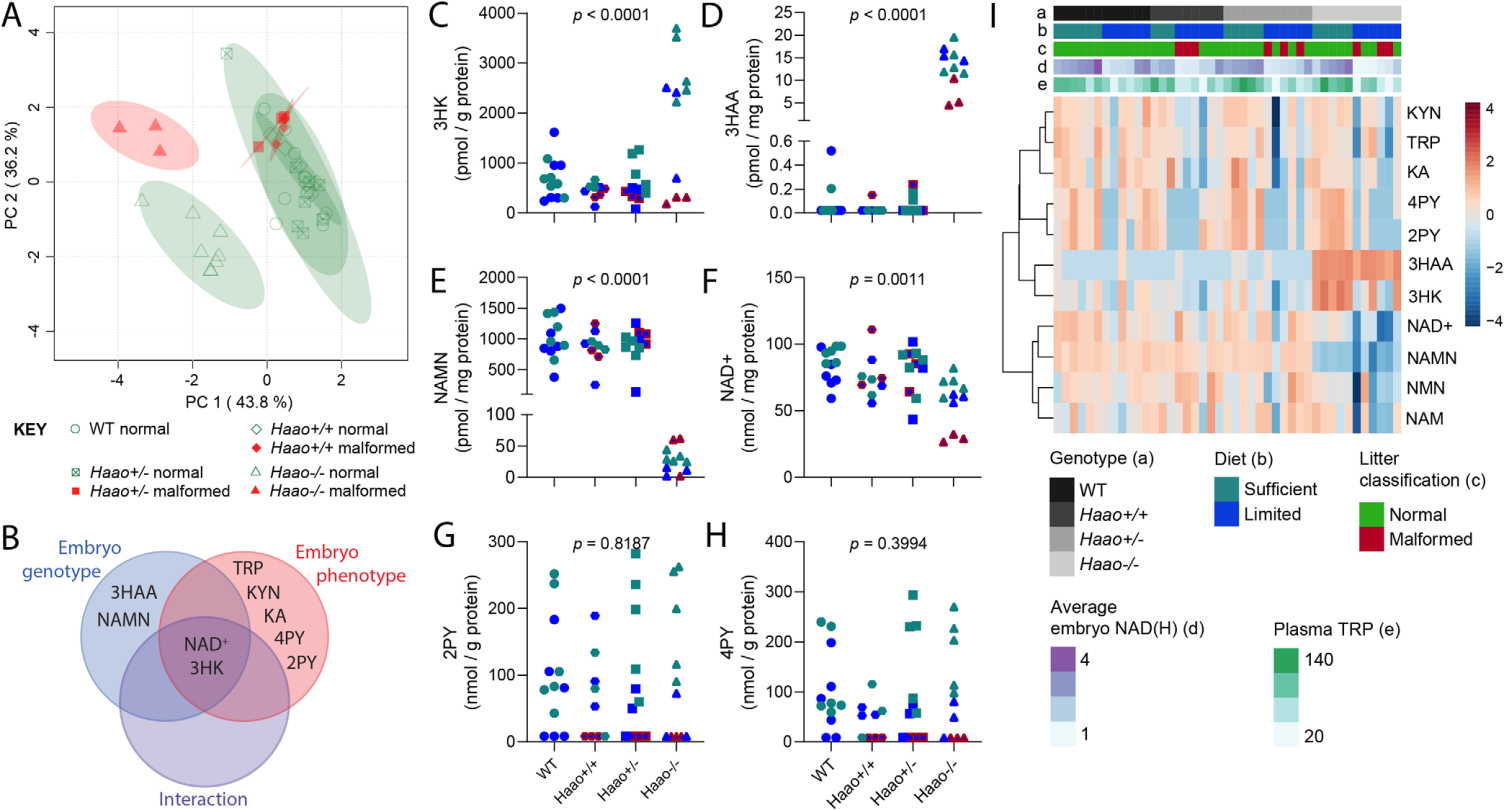
Dietary NAD precursor restriction induces metabolic adaptations in the yolk sac NAD metabolome at E11.5. (A) Principal component analysis (PCA) of concentrations of 11 metabolites of the NAD metabolome in E11.5 yolk sacs. Concentration values were normalized to protein concentration of the sample and standardized. Data from yolk sacs of WT × WT matings and *Haao* ^+/−^ × *Haao* ^+/−^ matings and of both maternal diets are included. Samples are also designated according to the phenotype of the respective embryos (normal or malformed). The PCA indicates two independent variables, embryo genotype (PC 1) and embryo phenotype (PC 2) separating the groups. (B) Venn diagram summarizing a two‐way ANOVA of the standardized yolk sac metabolite values as in (A). Metabolites with concentrations that differed significantly (*p* < 0.05) between embryo genotypes, embryo phenotypes, or both are indicated. (C–H) Concentrations of selected metabolites of the yolk sac NAD metabolome. To allow analysis, metabolite values below the limit of detection were given a value half of the detection limit for the respective metabolite. The *p* values represent one‐way ANOVA results comparing metabolite levels across genotypes. Datapoint colors indicate diets, and red outline indicates malformed litters. For the other quantified metabolites, see Figure S5. (I) Heatmap showing standardized metabolite concentrations in E11.5 yolk sacs. Data are organized by *Haao* genotype, diet, litter phenotype, average embryo NAD(H) concentration (nmol/mg protein), and nonstandardized maternal plasma TRP concentration (μM). See Table S18 for sample size and numerical values for panels (A), (B), and (I). WT, wild‐type; NAD(H), sum of NAD^+^ and NADH; TRP, L‐tryptophan; KYN, kynurenine; KA, kynurenic acid; 3HK, 3‐hydroxykynurenine; 3HAA, 3‐hydroxyanthranilic acid; NaMN, nicotinic acid mononucleotide; NMN, nicotinamide mononucleotide; NAM, nicotinamide; 2PY, N‐methyl‐2‐pyridone‐5‐carboxamide; 4PY, N‐methyl‐4‐pyridone‐5‐carboxamide.

To determine how different maternal diets affect yolk sac NAD *de novo* synthesis activity and how this correlates to embryo phenotypes, the yolk sac NAD metabolome was quantified at E11.5 (Table S18). Principal component (PC) analysis of eleven metabolites indicated two independent variables, embryo genotype (PC 1, 39.8%) and embryo phenotype (PC 2, 36.6%), as the main effectors of observed variation in the NAD metabolome (Figure 6A), with at least one of these variables significantly affecting nine of the metabolites (Figure 6B). Metabolites upstream (3HK, 3HAA) and downstream (NAMN) of HAAO activity significantly increased or decreased in *Haao* ^−/−^ yolk sacs, relative to *Haao* ^+/+^ and *Haao* ^+/−^, respectively (Figure 6C–E), thereby separating *Haao* ^−/−^ yolk sacs from the other genotypes (PC1, Figure 6A). This trend was independent of the maternal diet (Figure 6C–E).

The separation of the yolk sac NAD metabolome by embryo phenotype (PC2, Figure 6A) was driven by early NAD *de novo* synthesis metabolites (TRP, KYN, KA) and the Salvage Pathway excretion metabolites 2PY and 4PY (Figure 6B). Yolk sacs of malformed embryos had decreased abundance of these metabolites relative to normal embryos, suggesting NAD *de novo* pathway activity was decreased. HAAO enzyme activity in the yolk sac was proportional to the number of functional *Haao* alleles present and was independent of maternal diet (Figure S5A, Tables S16 and S17) and embryo phenotype (Figure S5A). Therefore, decreased pathway activity in the yolk sacs of malformed embryos appears to originate from decreased maternal provision of the TRP and other early pathway metabolites. Indeed, plasma TRP and KYN concentrations significantly correlated with those in the yolk sac (Figure S7A,B). Furthermore, plasma TRP and KYN significantly correlated with yolk sac early pathway intermediates including 3HK, with side‐branch product KA, as well as NAD^+^ (Figure S7D,E).

Two‐way ANOVA analyses also identified an interaction between embryo phenotype and genotype, associated with NAD^+^ and 3HK. Indeed, yolk sacs of malformed *Haao* ^−/−^ embryos had the lowest NAD^+^ levels (Figure 6F, red triangles) among all samples analyzed. Accumulation of 3HK, observed in yolk sacs of normal *Haao* ^−/−^ embryos and indicating NAD *de novo* synthesis activity, was reduced in their malformed counterparts (Figure 6C, red triangles). Given that HAAO enzymatic activity was not different, this reduced 3HK accumulation in yolk sacs of malformed embryos further confirmed that maternal provision of precursors for *de novo* NAD synthesis was lowest in these yolk sacs.

Together, this confirms that yolk sac NAD *de novo* synthesis activity is affected by maternal provision of TRP and/or KYN. Though most stark in *Haao* ^−/−^ yolk sacs, this decline in activity was also apparent in the yolk sacs of affected *Haao* ^+/+^ and *Haao* ^+/−^ embryos.

### 3.8 Dietary NAD Precursor Restriction Induces Metabolic Adaptations in the Yolk Sac NAD Metabolome at E11.5

Unlike the early NAD *de novo* synthesis pathway metabolites, the Salvage Pathway metabolites NAM and NMN did not separate by genotype or phenotype in E11.5 yolk sacs (Figure 6B, Figure S5E,F). On the Limited Diet, both NAM and NMN were highly variable between yolk sacs, but they correlated with each other and to NAD^+^ (Figure S6D–F). This variability was not seen with the Sufficient Diet (Figure S6A–C), suggesting that varying maternal NAD precursor provision to the yolk sac on the Limited Diet is directly reflected in Salvage Pathway activity. However, there was no overarching correlation between maternal plasma and yolk sac NAM (Figure S7C,F), indicating that instead it is the maternal TRP and KYN provision and yolk sac's NAD *de novo* synthesis activity that drive this difference, indicated by the positive correlation of plasma and yolk sac TRP and KYN levels (Figure S7A,B). As with the embryo proper (Figure 3M–P), *Haao* ^−/−^ yolk sacs were more dependent on maternal NAM provision than their littermates, with NAD^+^ levels positively correlating with maternal plasma NAM, unlike *Haao* ^+/+^ or *Haao* ^+/−^ littermates (Figure S8A–C).

Next, we assessed whether NAD *de novo* synthesis and the ability of *Haao* ^+/+^ and *Haao* ^+/−^ to maintain NAD levels in the yolk sac affected NAD levels in corresponding embryos, given that metabolite exchange can occur between components of the conceptus [8]. There was no significant correlation between embryo NAD and yolk sac NAD^+^ or NAM for any genotype (Figure S8D–I), suggesting NAD *de novo* synthesis in the yolk sac may primarily serve to maintain NAD levels in the yolk sac itself.

In summary, these data show that maternal NAD precursor availability is reflected in the yolk sac NAD metabolome. *Haao* ^+/+^ and *Haao* ^+/−^ yolk sacs have increased NAM and NMN levels, indicating that in addition to NAD *de novo* synthesis, they also have elevated Salvage Pathway activity, resulting in increased NAD levels compared to *Haao* ^−^^/‐^ yolk sacs. In contrast, as *Haao* ^−/−^ yolk sacs, like their embryos, are entirely dependent on maternal NAM provision and Salvage Pathway activity, they are at higher risk of becoming NAD deficient.

## 4 Discussion

CNDD is characterized by a spectrum of co‐occurring congenital malformations affecting the heart, kidney, limbs, vertebrae, and other organs and structures [5]. The phenotypes identified in patients are highly variable, even among those with loss‐of‐function variants in the same gene, and in one case even between siblings with the same causative variant [6, 11, 12, 13, 14, 23]. Previous studies in mice showed that nongenetic factors modulate the phenotypic severity in gene–environment interactions and that CNDD‐like malformations can be induced by environmental factors alone [15]. Here, we show that the significant litter‐to‐litter heterogeneity in CNDD phenotype severity is driven by differences in the maternal NAD metabolome, which are determined by the cumulative amount of NAD precursors provided in the diet.

Pregnant females were provided diets where the only NAD precursor was TRP. NAD *de novo* synthesis activity in the liver converts this TRP to NAM, which is then released into the circulation [7]. When provided with the Sufficient Diet, circulatory NAM levels are ample to maintain NAD levels in all embryos of the litter, including *Haao* ^−/−^ embryos that lack the capacity to use TRP. Therefore, embryo NAD levels are consistent within and between litters, and all embryos develop normally. By contrast, circulatory NAD metabolite levels are lower and differ between pregnant females on the Limited Diet, as observed previously [13]. Given that females were controlled for age, weight, and genotype, these differences are either driven by behavioral differences in dietary consumption or physiological differences in metabolic turnover.

The variability in circulatory NAD metabolite levels on the Limited Diet is mirrored by embryo NAD levels, which also vary significantly between, but not within, litters. Yet, litters with the lowest embryo NAD level were not always those that were malformed, suggesting that snapshot NAD measurements poorly reflect embryo phenotype at all stages assessed. This is for several reasons. First, embryo NAD levels are dynamic. In those litters where embryos become NAD deficient, the deficiency is transient and can occur at different times. Second, there are delays between when an embryo becomes NAD deficient, the molecular events that perturb development, and the subsequent formation of structural defects. Third, at early embryonic stages, phenotyping is limited to identifying defects in external structures. Therefore, measuring embryo NAD levels at one stage in development does not reflect the NAD status that led to their phenotype, nor how their current NAD status will impact subsequent stages of development.

Instead, because of its direct effect on modulating embryo NAD levels, the maternal circulatory NAD metabolome is a better predictor of phenotypic outcome. Our work shows embryos predominantly use maternally provided NAM to generate NAD via the Salvage Pathway. Both standard correlation and Random Forest analyses relatively accurately classified litters to pregnancy outcome and identified the Salvage Pathway excretion metabolites 2PY and 4PY as having the strongest association with adverse pregnancy outcome. These metabolites are known to reflect available NAM levels in humans and mice [13, 18, 19, 20], with their production elevated when circulatory NAM is in excess [19, 20, 24] but decreased when it is sparse [13]. The utility of the algorithm is further exemplified by its prediction that four litters without visible external defects at E9.5 or E11.5 but low NAD levels were misclassified as “normal,” because their metabolic profile resembled that of affected litters, meaning they might have developed defects or died at later stages. Therefore, whether NAM provision is sufficient for normal development is reflected by, and can be predicted using, plasma 2PY and 4PY levels.

We also showed that NAM, 2PY, and 4PY levels are reflective of dietary NAD precursor provision in humans. Thus, these metabolites represent easily measurable potential biomarkers to identify women with insufficient circulatory NAD precursors and an increased risk of adverse pregnancy outcomes. The window of birth defect susceptibility, organogenesis, occurs very early in human pregnancy, between weeks 3–8 postfertilization [25]. To prevent CNDD, any intervention needs to be similarly early, ideally before conception. Future studies are needed to establish and define normal circulatory metabolite concentration ranges and thresholds of insufficiency prior to and during pregnancy. This will enable the development of metabolic tests to identify at‐risk women who would benefit from NAD precursor supplementation to prevent potential adverse pregnancy outcomes.

Our mouse work also shows that the ability of the conceptus to use given NAD precursors from the maternal circulation affects the severity of the pregnancy outcome when overall levels are limited. This is reflective of human patients, with heterozygous‐null siblings unaffected by CNDD due to their ability to perform NAD *de novo* synthesis. Therefore, risk predictions for pregnancy outcome and subsequent intervention strategy need to consider the carrier status of both parents. To achieve this, we must fully understand the relationship between NAD synthesis capability in the conceptus and how this is shaped by the environment.

The gene–environment interaction governing defect formation in mice is equally complex. Both here and in previous work, we have shown that embryonic genotype composition matters, with *Haao* ^−/−^ embryos always more severely affected than their littermates, and those phenotypic outcomes less severe in litters lacking *Haao* ^−/−^ embryos [15]. Here, we demonstrate that this is because *Haao* ^−/−^ embryos and their extraembryonic tissues, such as the yolk sac, have an increased dependency on maternal NAM provision due to their inability to perform NAD *de novo* synthesis. The yolk sac is the only conceptal site of NAD *de novo* synthesis during organogenesis [8], when CNDD‐susceptible organs likely develop defects. Here, we show for the first time that the pathway is active by at least E9.5, for example, when disruption of kidney ureteric bud formation and outgrowth at E9.5–E10.5 would result in kidney agenesis, a common CNDD‐related defect in humans [5] and mice [15, 18]. Further, yolk sac NAD *de novo* synthesis activity is proportional to maternal provision of TRP and is maintained even after the embryonic liver is formed [26] and is functional.

When maternal NAD precursor supply declines, yolk sacs appear to adapt, conserving NAD via increased Salvage Pathway activity and reduced excretion metabolite (2PY, 4PY) production. These efforts to conserve NAD were unsuccessful in the yolk sacs of malformed *Haao* ^−/−^ embryos, resulting in decreased NAD^+^ levels, confirming that maternal NAM provision in these litters was insufficient to maintain NAD levels in the absence of additional NAD generation via NAD *de novo* synthesis.

Furthermore, our work also has implications for emerging areas of research that examine metabolism in mammalian embryogenesis. Despite fundamental anatomical differences and different timing when a yolk sac is present in mice and humans, many yolk sac functions are conserved between the species [27], likely including NAD *de novo* synthesis activity [8]. In particular, the yolk sac absorbs macro‐ and micronutrients via active pinocytosis, degrades them, and transfers the products to the embryo [28]. The yolk sac has metabolic activity, including NAD *de novo* synthesis, and therefore modulates and controls metabolite supply to the embryo. Disruption of these processes can induce defect formation [29]. Therefore, an NAD deficiency in the yolk sac itself is likely to have additional consequences on embryonic development that would likely exacerbate any embryonic NAD deficiency. How NAD metabolism in the human yolk sac contributes to embryonic NAD status requires further investigation, as does the role the yolk sac plays in human CNDD patients and in congenital defect formation.

CNDD patients with biallelic loss‐of‐function variants in *Haao* have strongly elevated levels of 3HAA [6]. Here, we found that this metabolic dysfunction in murine *Haao* ^−/−^ conceptuses affects the maternal plasma NAD metabolome. The presence of *Haao* ^−/−^ embryos in a litter can be identified in the mother's circulation, with the level of 3HAA, the substrate of HAAO, being proportional to the number of *Haao* ^−/−^ embryos. There is no comparable NAD metabolome data available for human CNDD pregnancies. If a fetus with biallelic loss‐of‐function of NAD *de novo* synthesis activity elicits such a metabolic signature in the maternal circulation as seen in mice, it would provide an early gestational marker of CNDD. This would allow early and continued intervention with vitamin B3 supplementation to mitigate adverse developmental and postnatal effects of NAD deficiency; for example, postbirth pellagra‐like skin symptoms [13]. Furthermore, the excessive levels of metabolites upstream of the genetic block are likely risk factors of poor growth and altered neurodevelopment because, for example, KA, quinolinic acid, and 3HK have neuroactive and/or neurotoxic effects [30, 31] and perturbations of the NAD *de novo* Synthesis Pathway have been linked to neurological conditions such as schizophrenia, bipolar disorder, or Alzheimer's disease [32, 33, 34].

This principle of detecting genetic dysfunction in the fetus by metabolic testing of maternal plasma could be applicable to other metabolic disorders. Autosomal recessive diseases such as Hurler syndrome and Zellweger syndrome can be detected prenatally in the amniotic fluid by their increased glycosaminoglycan [35, 36] and very long chain fatty acid levels [37], respectively. Besides these, other inherited metabolic diseases such as phenylketonuria [38], medium‐chain acyl‐CoA dehydrogenase deficiency [39] and fetal glutaric aciduria type 1 [40] cause accumulation of specific metabolites postnatally and so may also be potentially detectable prenatally. Maternal blood tests during pregnancy for known signature metabolites of such disorders would allow early detection, enabling potential mitigation of adverse effects during pregnancy, immediate actioning after birth, and complementing genetic testing if the disease‐causing gene(s) are known.

## Author Contributions

K.B., H.C., and S.L.D. conceived the study; K.B., H.C., A.W.S., N.N., and S.L.D. designed the study; K.B., H.C., D.Z.S., A.S., and A.W.S. performed research and acquired data; K.B., H.C., D.Z.S., A.S., and A.W.S. analyzed data; S.L.D. and N.N. acquired financial support; H.C., K.B., and S.L.D. drafted the manuscript, and all authors contributed to data interpretation and revised the manuscript.

## Conflicts of Interest

The authors declare no conflicts of interest.

## Supporting information


Data S1.

## Data Availability

All data generated or analyzed during this study, except for detailed human clinical data, are included in this article and it's Files S1. Data from the human clinical study are available from the corresponding authors upon reasonable request.
