# Supplementary material for: Maternal Circulatory NAD Precursor Levels and the Yolk Sac Determine NAD Deficiency‐Driven Congenital Malformation Risk
Source: FASEB J. 2025 Jul 21;39(14):e70834. doi: 10.1096/fj.202500708RR (PMC12278891; doi:10.1096/fj.202500708RR)
Supplement: Supplementary file 1 — Data S1. [file FSB2-39-e70834-s001.docx]

**Supplementary Information for**

Maternal circulatory NAD precursor levels and the yolk sac determine NAD deficiency-driven congenital malformation risk

Kayleigh Bozon^1^, Hartmut Cuny^1,2,*^, Delicia Z. Sheng^1^, Alena Sipka^1^, Antonia Shand^3,4,5^, Natasha Nassar^3,5^, Sally L. Dunwoodie^1,2,*^

^1^Developmental and Stem Cell Biology Division, Victor Chang Cardiac Research Institute, Sydney, NSW 2010, Australia.

^2^School of Clinical Medicine, Faculty of Medicine and Health, University of New South Wales, Sydney, NSW 2052, Australia.

^3^Children’s Hospital at Westmead Clinical School, University of Sydney, Sydney, NSW 2145, Australia

^4^Department of Maternal Fetal Medicine, Royal Hospital for Women, Sydney, NSW 2031, Australia

^5^Menzies Centre for Health Policy and Economics, Faculty of Medicine and Health, University of Sydney, Sydney, NSW 2006, Australia

***Corresponding authors:**

Sally L. Dunwoodie **Email:** [s.dunwoodie@victorchang.edu.au](mailto:s.dunwoodie@victorchang.edu.au),

Hartmut Cuny **Email:** [h.cuny@victorchang.edu.au](mailto:h.cuny@victorchang.edu.au)

Victor Chang Cardiac Research Institute, 405 Liverpool Street, Darlinghurst NSW 2010, Australia

P: +61 2 9295 8613

**Table of contents**

| **Content** | **Page** |
| --- | --- |
| **Supplemental Results** | **3** |
| **Supplemental Figures** | **4** |
| **Figure S1.** Maternal dietary restriction of NAD precursors causes varying degrees of adverse pregnancy outcomes | 4 |
| **Figure S2.** The maternal plasma NAD metabolome changes as pregnancy progresses and is affected by dietary NAD precursor restriction | 5 |
| **Figure S3.** Correlation between embryo NAD(H) concentration and maternal plasma NAM at E14.5 | 6 |
| **Figure S4.** NAD *de novo* synthesis is active in the yolk sac at E9.5 | 7 |
| **Figure S5.** Dietary NAD precursor restriction induces metabolic adaptations in the yolk sac NAD metabolome at E11.5 | 8 |
| **Figure S6.** Correlation between NAD^+^ and NAD Salvage Pathway metabolite (NAM, NMN) concentrations in E11.5 yolk sacs | 9 |
| **Figure S7.** Maternal provision of NAD-related metabolites dictates yolk sac NAD *de novo* synthesis activity at mid-gestation | 10 |
| **Figure S8.** Correlations of yolk sac metabolite concentrations with maternal plasma NAM and embryo NAD(H) concentrations | 11 |
| **Supplemental Tables** | **12** |
| **Table S1.** Overview of mouse diets and abbreviations | 12 |
| **Table S2.** Summary of embryo outcomes at E9.5, E11.5 and E14.5 under different gene-environment conditions | 13 |
| **Table S3.** Summary of types and incidence of congenital malformations observed at E11.5 | 14 |
| **Table S4.** Summary of types and incidence of congenital malformations observed at E14.5 | 15 |
| **Table S5.** E9.5 embryo phenotyping details | 16 |
| **Table S6.** E11.5 embryo phenotyping details | 17 |
| **Table S7.** E14.5 embryo phenotyping details | 18 |
| **Table S8.** E9.5 maternal plasma NAD metabolome quantification | 19 |
| **Table S9.** E11.5 maternal plasma NAD metabolome quantification | 20 |
| **Table S10.** E14.5 maternal plasma NAD metabolome quantification | 21 |
| **Table S11.** Summary of embryo NAD(H) concentrations at E9.5, E11.5 and E14.5 | 22 |
| **Table S12.** E9.5 embryo NAD(H) concentrations | 23 |
| **Table S13.** E11.5 embryo NAD(H) concentrations | 24 |
| **Table S14.** E14.5 embryo NAD(H) concentrations | 26 |
| **Table S15.** Concentrations of NAD^+^ and related metabolites in E9.5 yolk sacs | 28 |
| **Table S16.** Summary of HAAO enzyme activity measured in yolk sacs collected at E11.5 | 29 |
| **Table S17.** HAAO enzyme activity in E11.5 yolk sacs | 30 |
| **Table S18.** Concentrations of NAD^+^ and related metabolites in E11.5 yolk sacs | 31 |
| **SI References** | **33** |

Supplemental Results

**Embryo phenotypes.** We compared two different dietary models, based on a previous study^1^ which are NAD precursor vitamin-depleted feed (NF), designated Sufficient Diet, and NAD precursor vitamin-depleted and TRP-free feed and 600 mg/L of TRP in the drinking water (NTF+TW600), designated Limited Diet (Table S1), at three times of gestation. Furthermore, to identify how loss-of-function *Haao* alleles influence the phenotypic variability between litters, we compared litters from wild-type (WT) females mated with WT males to litters from matings of *Haao^+/-^* females with *Haao^+/-^* males, with the latter generating embryos of all three *Haao* genotypes in Mendelian ratio. Litters were collected at E9.5, E11.5 and E14.5 (Figure S1).

As observed previously^1-3^, maternal dietary NAD precursor restriction as with the Limited Diet induced adverse pregnancy outcomes in WT mothers (Table S2). Litter outcomes were heterogeneous, encompassing entirely unaffected as well as entirely dead litters (Figure S1), similar to previous observations^1^. The incidence of embryo death was higher at E11.5 and E14.5 compared to E9.5, suggesting that embryos died either between implantation and E9.5 or between E9.5 and E11.5. The same trend was evident with litters from *Haao*+/- mothers (Table S2). One embryo of the Limited Diet condition was developmentally delayed at E9.5 (Figure S1, Table S5), a phenotype observed previously^1^. At E9.5, many organs and structures of interest are at the early stages of development. Thus, embryos cannot be extensively phenotyped for Congenital NAD deficiency disorder (CNDD) related anomalies like this is possible at later stages when they have grown and their organs and structures are developed further. However, one E9.5 embryo of the Limited Diet group had exencephaly (Table S5).

At E11.5, malformations in the same organs/structures were observed as at E14.5, including in the neural tube, eyes, limbs, tail, as well as occurrences of oedema (Table S3, Table S4, Table S6, Table S7), indicating these malformations were caused prior to E11.5. Additional malformation types were identified at E14.5, which either derived from tissues being further developed (digits, jaw) or from internal organs that could be more readily assessed at this later time point, such as the kidneys. Malformations occurred in embryos of all *Haao* genotypes (Table S4).

**NAD *de novo* synthesis is active in the yolk sac at E9.5, but embryo NAD levels primarily rely on maternal plasma nicotinamide (NAM) provision at this stage.** NAD *de novo* synthesis is functionally active in the yolk sac at E10.5 and intermediate metabolites are extensively exchanged between the yolk sac and embryo at this stage^4^. To determine whether the NAD *de novo* Synthesis Pathway is already functionally active earlier in development, when some of the CNDD-susceptible organs are starting to develop, we quantified E9.5 yolk sac NAD metabolomes using ultra-high performance liquid chromatography-tandem mass spectrometry (UHPLC-MS/MS). The yolk sacs corresponded to the WT litters of which the maternal NAD metabolomes and embryo NAD(H) levels were measured. Given their limited size at this stage of development, all yolk sacs were pooled to generate a single representative sample per litter.

The same metabolites as in E11.5 yolk sacs could be quantified, except for the Salvage Pathway waste metabolites N-methyl-2-pyridone-5-carboxamide (2PY) and N-methyl-4-pyridone-5-carboxamide (4PY) irrespective of dietary treatment (Figure S4A, Table S15). Yolk sac NAD^+^ levels significantly positively correlated with NAD *de novo* Synthesis Pathway intermediates kynurenine (KYN), 3-hydroxykynurenine (3HK), quinolinic acid (QA), and nicotinic acid mononucleotide (NAMN), but not with yolk sac L-tryptophan (TRP), NAM, or nicotinamide mononucleotide (NMN) (Figure S4B). Yet, all measured NAD *de novo* Synthesis Pathway intermediates from TRP to NAD^+^ in E9.5 yolk sacs significantly positively correlated with maternal plasma TRP, whereas yolk sac TRP and NAM levels did not correlate with this maternal NAD precursor (Figure S4C). No significant correlation was seen with maternal plasma NAM and any of the yolk sac metabolites (Figure S4D).

Together, this suggests that NAD *de novo* synthesis activity in the yolk sac from maternally provided TRP contributes significantly to the NAD^+^ levels in the E9.5 yolk sac. However, as embryo NAD and yolk sac NAM levels do not correlate positively (Figure S4E), NAD levels in the embryo proper are mostly driven by maternal plasma NAM provision at this early organogenesis stage.

**
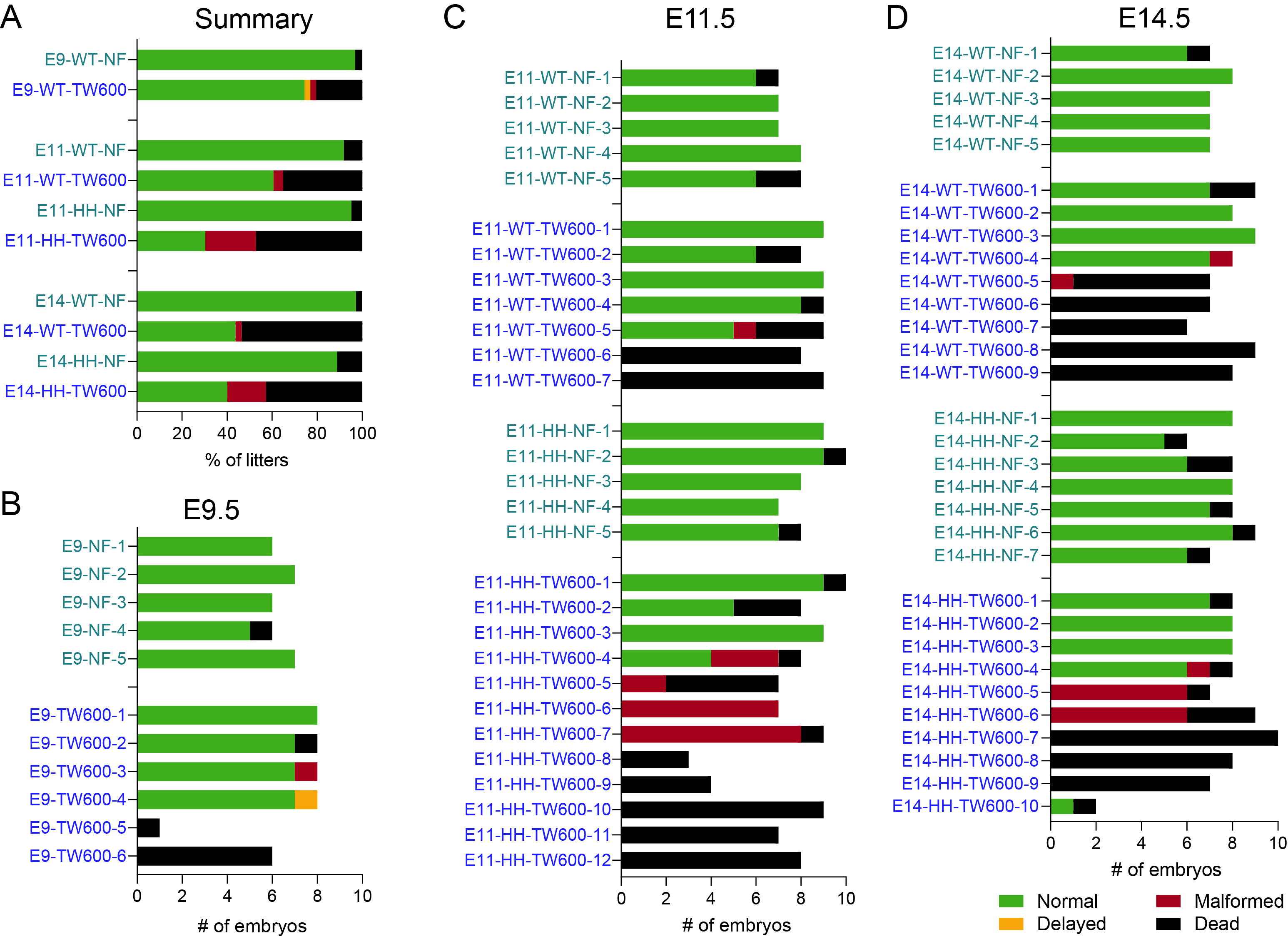
**

**Figure S1. Maternal dietary restriction of NAD precursors causes varying degrees of adverse pregnancy outcomes.** (*A*) Summary of the phenotypic outcomes of embryos from wild-type (WT) mothers mated with WT males and *Haao*+/- mothers mated with *Haao*+/- males at E9.5, E11.5, and E14.5. Each label includes information about the embryonic stage (E9 = E9.5, E11 = E11.5, E14 = E14.5), the mating scheme (WT = wild-type x wild-type mating, HH = *Haao*+/- x *Haao*+/- mating), and the maternal diet given throughout pregnancy as NF = Sufficient Diet (teal) and TW600 = Limited Diet (blue). (*B*)-(*D*): Datasets of (*A*), are separated by litters at E9.5 (B), E11.5 (C) and E14.5 (D). Each horizontal bar represents a litter, and the length of the bars indicates the number of embryos per litter. Total counts and percentages of embryos within each treatment group are summarized in Table S2. Details of the phenotypes of E11.5 and E14.5 embryos are given in Tables S3 and S4, respectively. E9.5 embryos phenotypically resembling E8.5 or younger embryos were defined as ‘delayed’. All dead embryos were found to be early resorptions. Only litters from WT x WT matings were collected at E9.5.


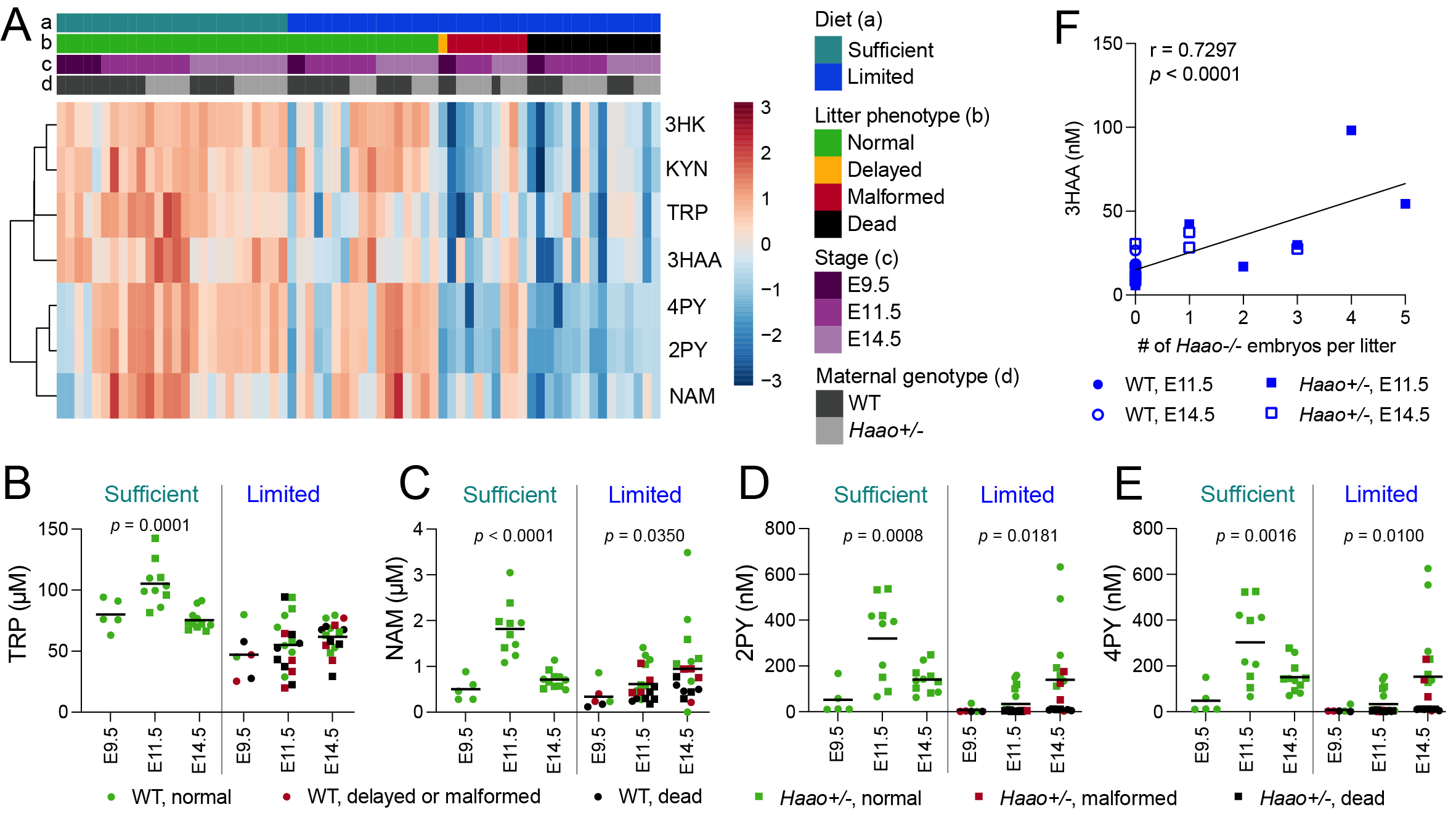


Figure S2. The maternal plasma NAD metabolome changes as pregnancy progresses and is affected by dietary NAD precursor restriction. (*A*) Heatmap showing standardized levels of NAD-related metabolites in the plasma of pregnant wild-type (WT) and *Haao*+/- mice provided either Sufficient Diet or Limited Diet. Data are organized by diet (a), litter phenotype (b), gestational stage (c), and *Haao* genotype (d). See Tables S8,9,10 for sample size and numerical values. (*B*)-(*E*): Concentrations of maternal plasma TRP (*B*), NAM (*C*), 2PY (*D*), and 4PY (*E*) at the three different stages and with the two different diets. Colors indicate whether the litter consisted of normal (green), delayed or malformed (red) or dead (black) embryos. Horizontal line indicates the mean. The *p* values indicate one-way ANOVA results comparing metabolite levels across the three timepoints within the same diet group. (*F*) Spearman’s correlation (r) of maternal plasma 3HAA with the number of *Haao*-/- embryos per litter at E11.5 and E14.5 from WT and *Haao*+/- females provided the Limited Diet. WT, wild-type; TRP, L-tryptophan; KYN, kynurenine; 3HAA, 3-hydroxyanthranilic acid; NAM, nicotinamide; 2PY, N-methyl-2-pyridone-5-carboxamide; 4PY, N-methyl-4-pyridone-5-carboxamide.


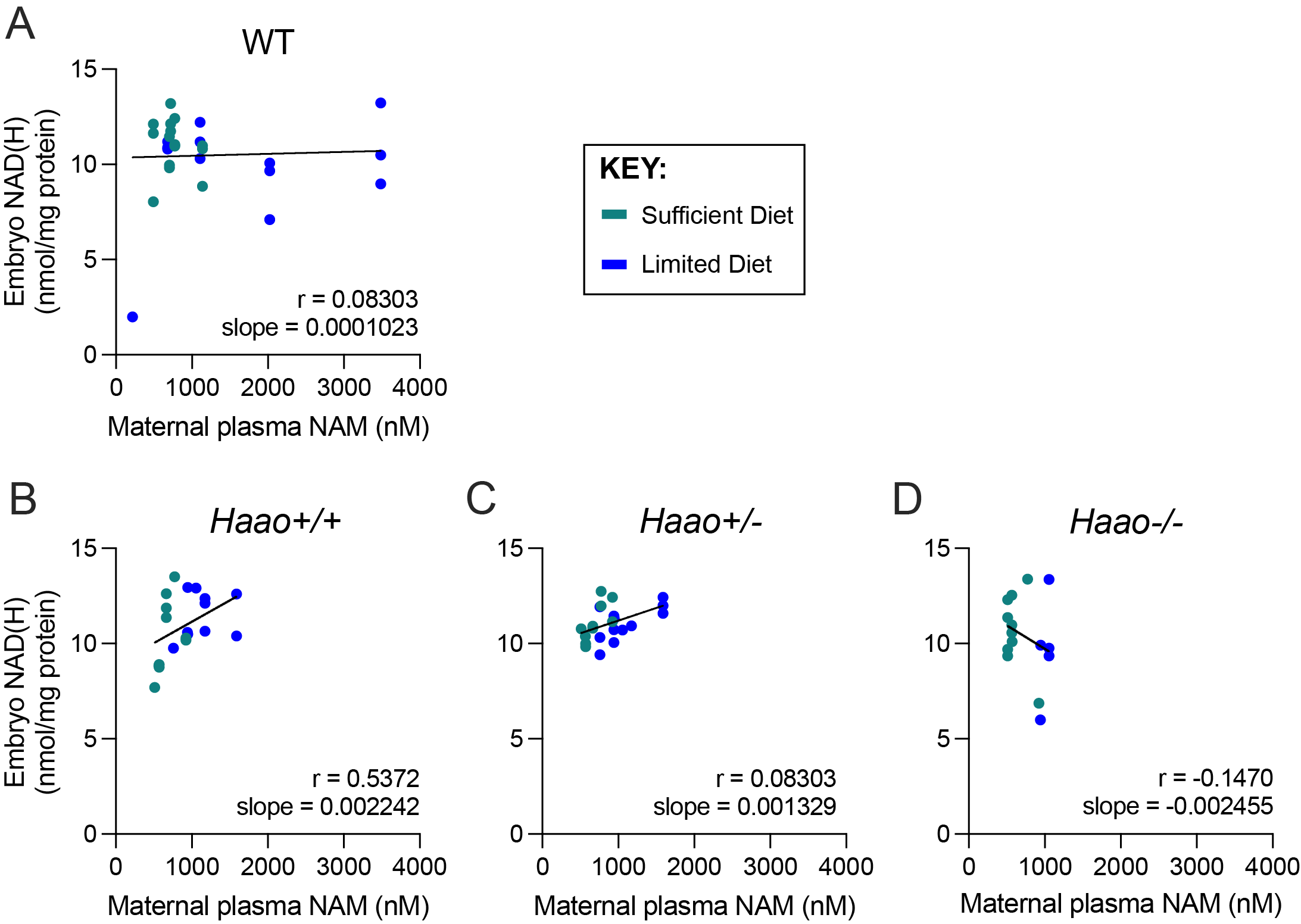


Figure S3. Correlation between embryo NAD(H) concentration and maternal plasma NAM at E14.5. (*A*) Wild-type (WT) embryos from WT mothers, (*B*)-(*D*) embryos of *Haao*+/- x *Haao*+/- matings, separated by *Haao* genotype. Spearman’s correlation (r) and slope are indicated for each graph. Colors indicate maternal diets. NAM, nicotinamide; NAD(H), sum of NAD^+^ and NADH.


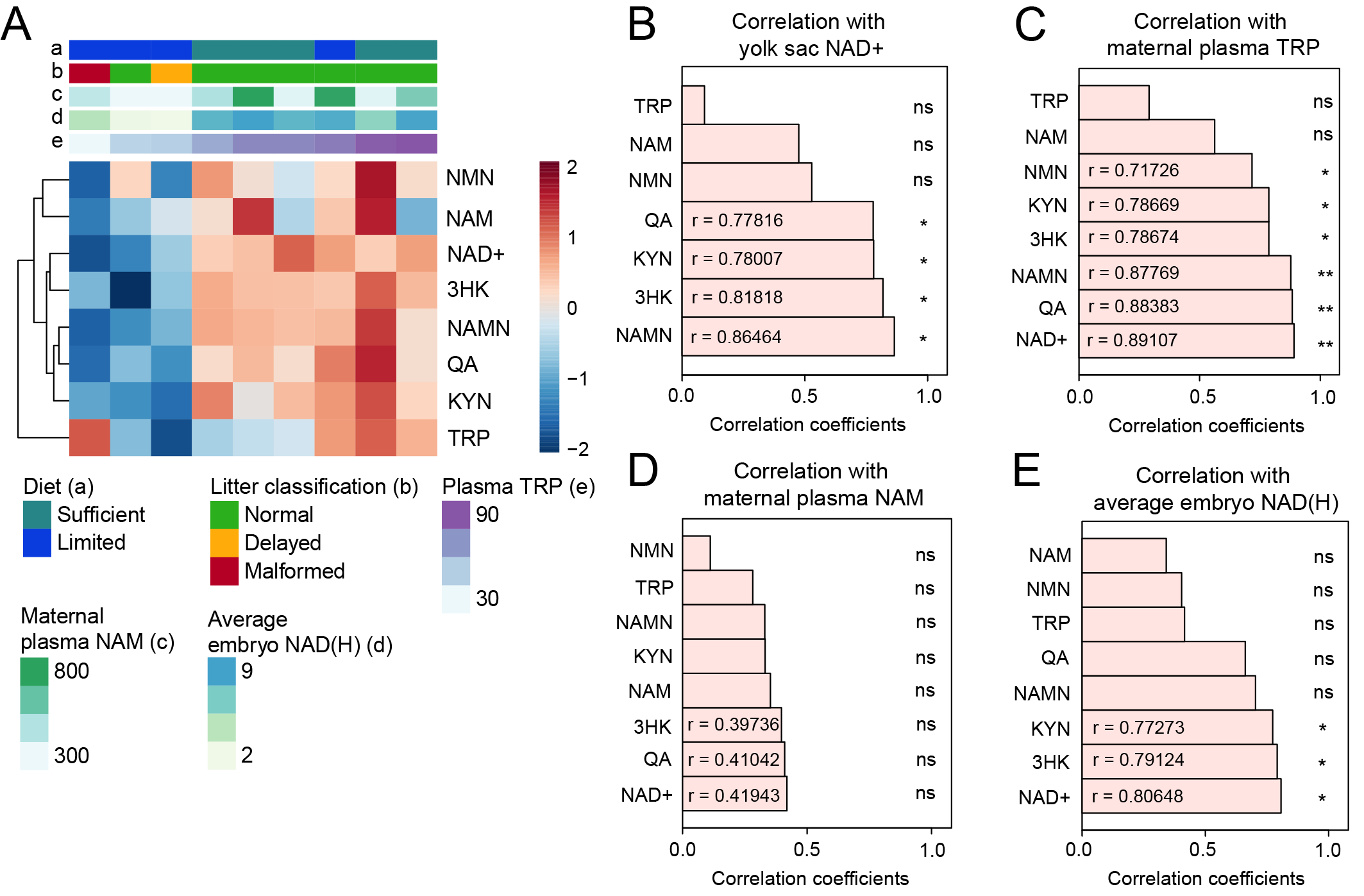


**Figure** **S4.** **NAD *de novo* synthesis is active in the yolk sac at E9.5.** (*A*) Heatmap showing standardized concentrations of nine measured NAD-related metabolites in E9.5 yolk sacs. Data are organized by diet (a), litter classification (b), non-standardized maternal plasma NAM concentration (nM) (c), average embryo NAD(H) concentration (nmol/mg protein) of the respective litters (d), and non-standardized maternal TRP concentration (µM) (e). (*B*)-(*E*) Pearson’s correlation (r) of standardized yolk sac metabolite levels with yolk sac NAD^+^ (*B*), maternal plasma TRP (*C*), maternal plasma NAM (*D*), and embryo NAD(H) (*E*). *q<0.05; **q<0.01; ns, not significant. See Table S15 for sample size and numerical values. TRP, L-tryptophan; KYN, kynurenine; 3HK, 3-hydroxykynurenine; QA, quinolinic acid; NaMN, nicotinic acid mononucleotide; NMN, nicotinamide mononucleotide; NAM, nicotinamide; NAD(H), sum of NAD^+^ and NADH.


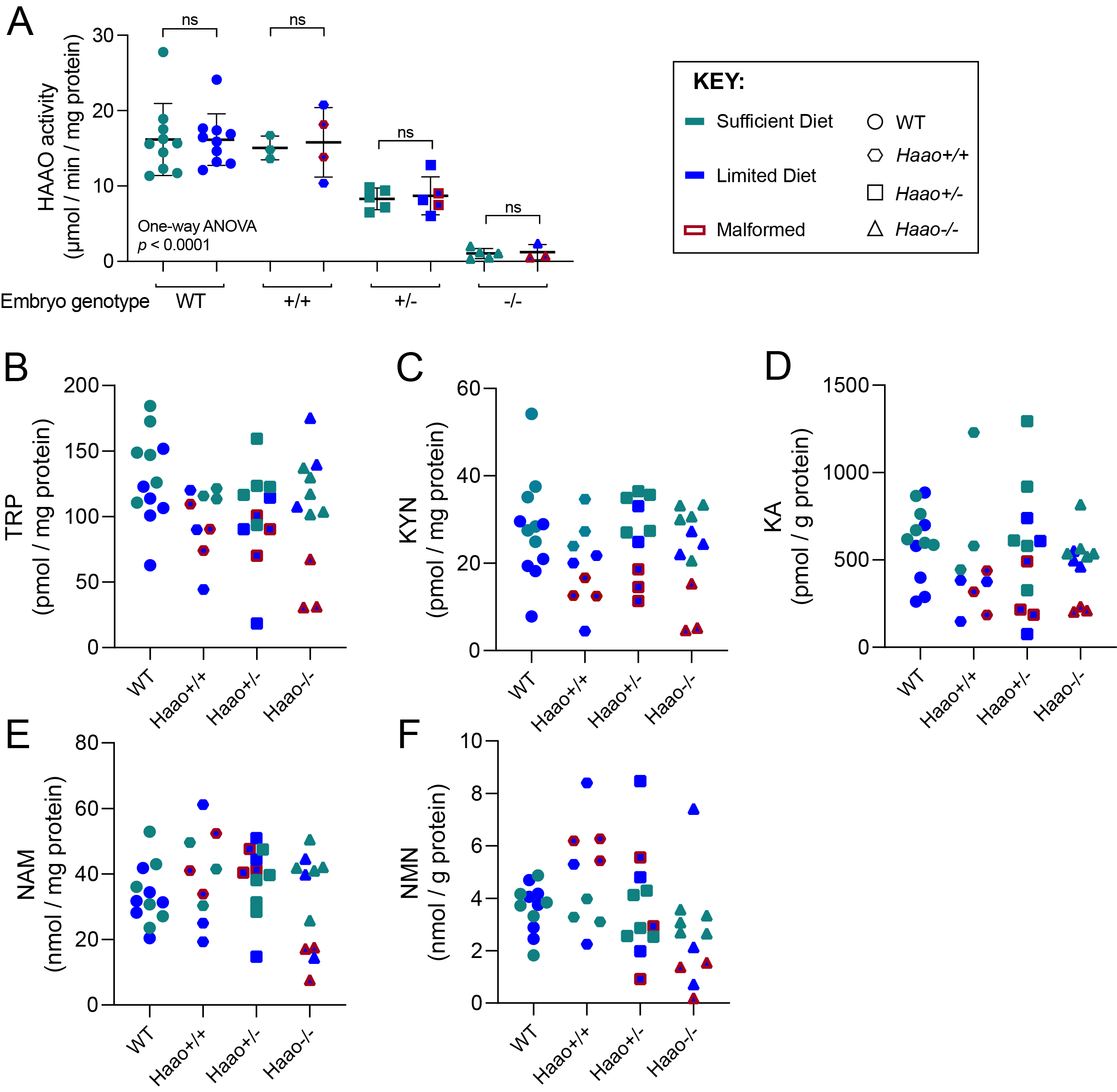


Figure S5. Dietary NAD precursor restriction induces metabolic adaptations in the yolk sac NAD metabolome at E11.5. (*A*) Enzymatic HAAO activity measured in E11.5 yolk sacs collected either from pregnant WT females mated with WT males or from pregnant *Haao*+/- females mated with *Haao*+/- males (generating *Haao*+/+, *Haao*+/-, and *Haao*-/- conceptuses) and provided either Sufficient or Limited Diet during pregnancy. Overall difference in HAAO activity was determined by one-way ANOVA. One-way ANOVA with Dunn’s multiple comparisons test was used to compare pairs. (*B*-*F*) Concentrations of selected metabolites of the yolk sac NAD metabolome at E11.5, separated by genotype. Datapoint shapes represent *Haao* genotypes, colors represent diets, and red outline indicates malformed litters. For the other quantified metabolites, see Figure 4. TRP, L-tryptophan; KYN, kynurenine; KA, kynurenic acid; NAM, nicotinamide; NMN, nicotinamide mononucleotide; WT, wild-type yolk sacs from wild-type matings.

**
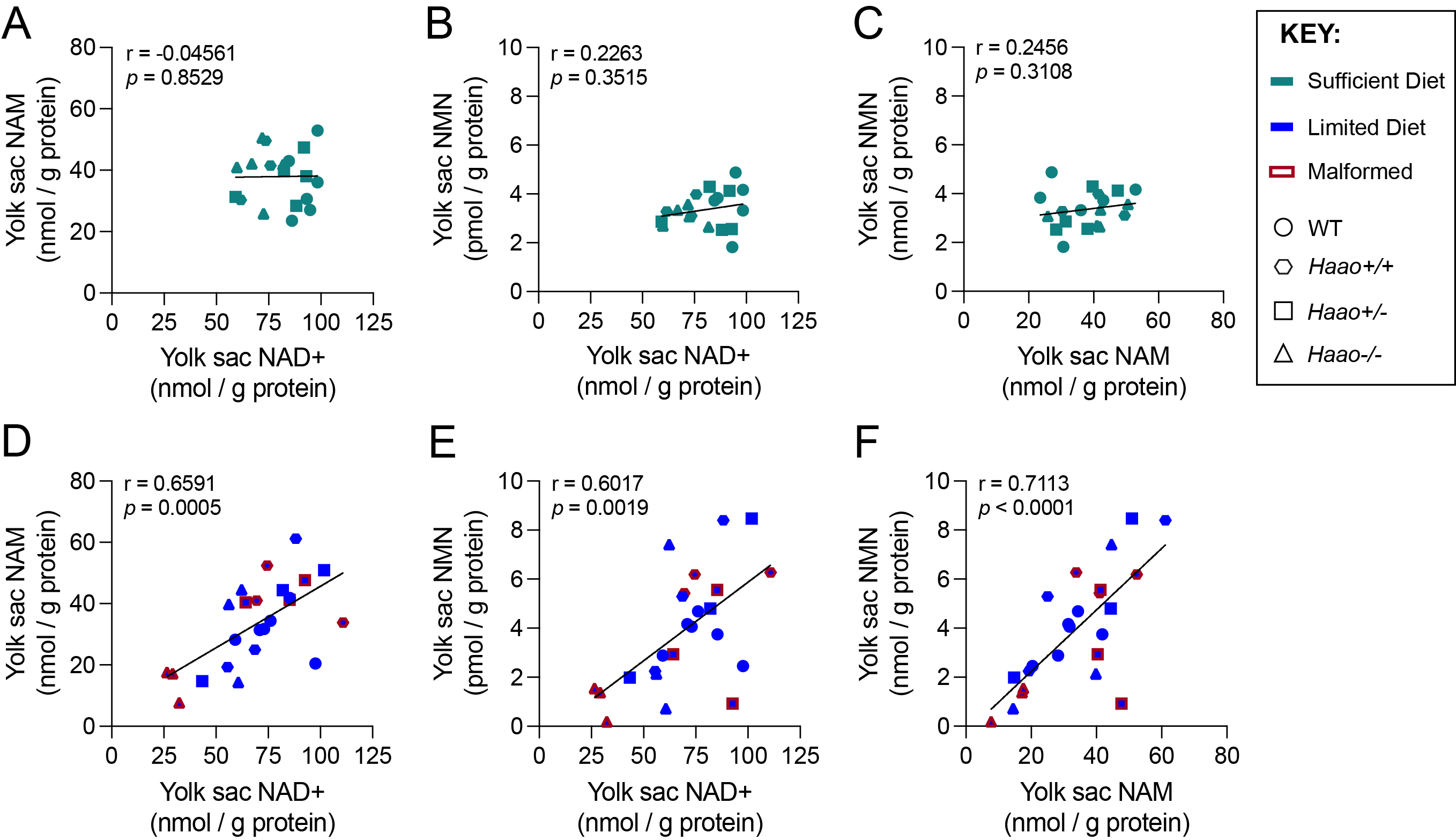
**

**Figure** **S6. Correlation between NAD^+^ and NAD Salvage Pathway metabolite (NAM, NMN) concentrations in E11.5 yolk sacs.** Samples were collected from mice on the Sufficient Diet (A-C) or the Limited Diet (*D-F*). Spearman’s correlation (r) and its significance (*p*, two-tailed) are indicated for each graph. WT, wild-type embryos from wild-type matings; NAM, nicotinamide; NMN, nicotinamide mononucleotide.

**
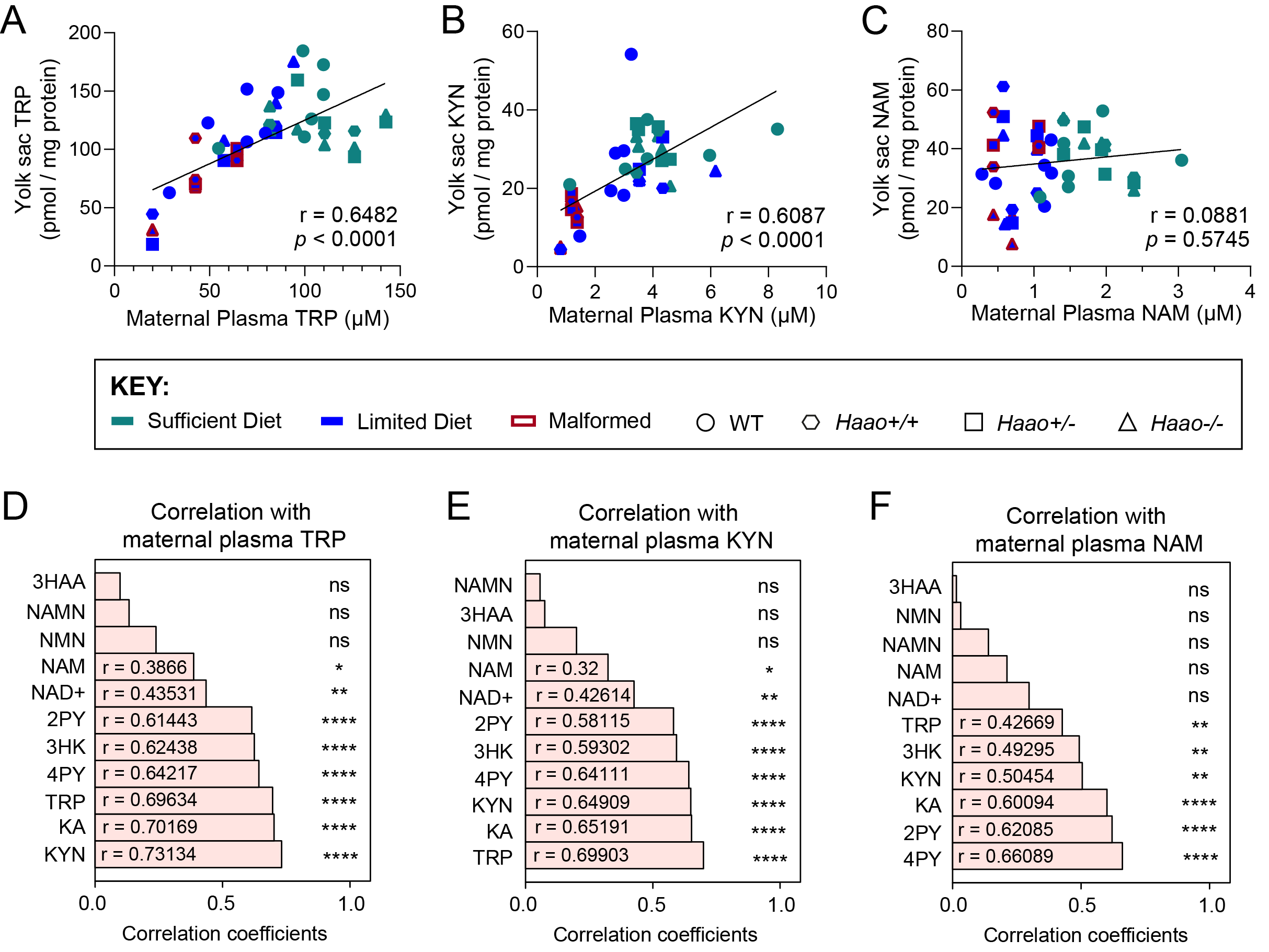
**

**Figure** **S7. Maternal provision of NAD-related metabolites dictates yolk sac NAD *de novo* synthesis activity at mid-gestation.** (*A*), (*B*), (*C*) Correlation between concentrations of maternal plasma TRP (*A*), KYN (*B*), NAM (*C*) and their respective concentration in the yolk sac at E11.5. Spearman’s correlation (r) and its significance (*p*, two-tailed) are indicated for each graph. (*D*), (*E*), (*F*) Pearson’s correlation between standardized concentrations of maternal plasma TRP (*D*), KYN (*E*), and NAM (*F*) and various NAD-related metabolites measured in yolk sacs. *q<0.05, **q<0.01; ***q<0.001; ****q<0.0001; ns, not significant. See Tables S9 and S18 for sample size and numerical values. WT, wild-type embryos from wild-type matings; TRP, L-tryptophan; KYN, kynurenine; KA, kynurenic acid; 3HK, 3-hydroxykynurenine; 3HAA, 3-hydroxyanthranilic acid; NaMN, nicotinic acid mononucleotide; NMN, nicotinamide mononucleotide; NAM, nicotinamide; 2PY, N-methyl-2-pyridone-5-carboxamide; 4PY, N-methyl-4-pyridone-5-carboxamide.

**
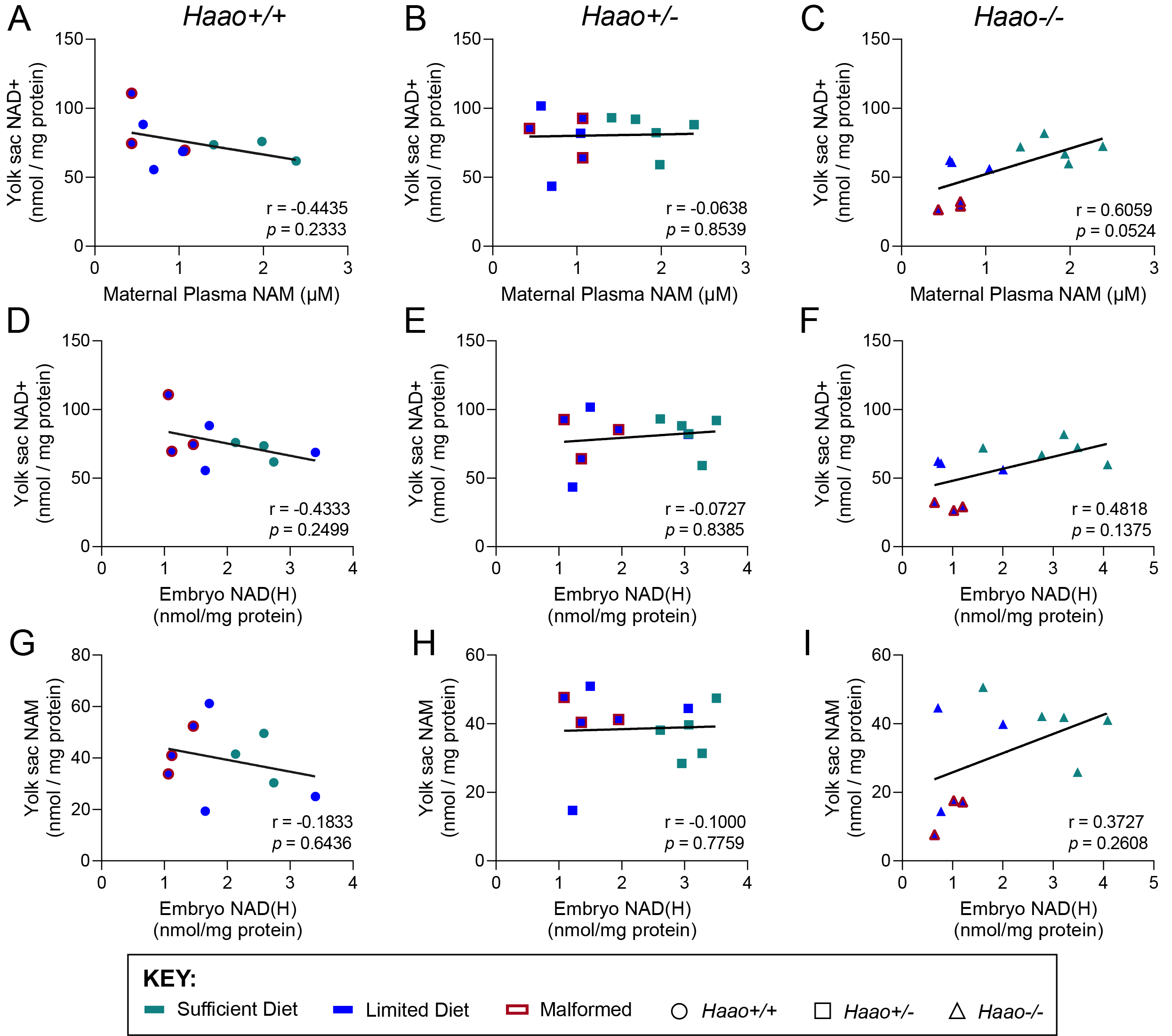
**

**Figure** **S8. Correlations of yolk sac metabolite concentrations with maternal plasma NAM and embryo NAD(H) concentrations.** (*A-C*) Correlation between yolk sac NAD^+^ and maternal plasma NAM, separated by yolk sac *Haao* genotype. (*D-F*) Correlation between yolk sac NAD^+^ and embryo NAD(H). (*G*-*I*) Correlation between yolk sac NAM and embryo NAD(H). Spearman’s correlation coefficient (r) and two-tailed *p* value are indicated within each graph. TRP, L-tryptophan; NAM, nicotinamide; NAD(H), sum of NAD^+^ and NADH.

Table S1. Overview of mouse diets and abbreviations

| Diet abbreviation | Diet designation | Feed code^1^ | NAD precursors in feed (mg/kg) | | | TRP in water (mg/L) | Dietary NAD precursors | |
| --- | --- | --- | --- | --- | --- | --- | --- | --- |
|  |  |  | NA | NAM | TRP |  | (µg/day)^2^ | (% NF)^3^ |
| Standard | Standard | SF22-100 | 0.5 | 29.5 | 2700 | 0 | 292.5 | 238.8 |
| NF | Sufficient | SF16-049 | 0 | 1.4 | 1800 | 0 | 122.5 | - |
| NTF+TW600 | Limited | SF16-097 | 0 | 1.4 | 0 | 600 | 67.5 | 55.1% |

^1^Product code of the manufacturer (Specialty Feeds, Glen Forrest, Australia).

^2^Estimated amounts of NAD precursors per day are based on an average consumption of 3.9 g food and 6.2 mL water per day and on the approximation that 60 mg of dietary tryptophan is equivalent to 1 mg of vitamin B3 for the conversion to NAD^5,6^. The Sufficient Diet is considered sufficient in dietary NAD precursors based on the calculated daily NAD precursor provision which can be converted to human equivalent values^1^.

^3^The percentages refer to the NAD precursor supply (vitamin B3 and tryptophan) relative to Sufficient Diet.

TRP, tryptophan; NA, nicotinic acid; NAM, nicotinamide; NF, NAD precursor vitamin-depleted feed; NTF, NAD precursor vitamin-depleted and tryptophan-free feed; TW, tryptophan-supplemented water.

Table S2. Summary of embryo outcomes at E9.5, E11.5 and E14.5 under different gene-environment conditions.

|  | Genotype | |  | Of all embryos | | | | Of live embryos | | | |
| --- | --- | --- | --- | --- | --- | --- | --- | --- | --- | --- | --- |
| Stage | M | P | Diet | Alive | | Dead | | Normal | | Malformed/  Delayed^1^ | |
| E9.5 | +/+ | +/+ | Sufficient | 31 | (96.9%) | 1 | (3.1%) | 30 | (96.8%) | 1 | (3.2%) |
|  | +/+ | +/+ | Limited | 31 | (79.5%) | 8 | (20.5%) | 29 | (93.5%) | 2 | (6.5%) |
| E11.5 | +/+ | +/+ | Sufficient | 34 | (91.9%) | 3 | (8.1%) | 34 | (100%) | 0 | (0%) |
|  | +/+ | +/+ | Limited | 38 | (62.3%) | 23 | (37.7%) | 37 | (97.4%) | 1 | (2.6%) |
|  | +/- | +/- | Sufficient | 40 | (95.2%) | 2 | (4.8%) | 40 | (100%) | 0 | (0%) |
|  | +/- | +/- | Limited | 47 | (52.8%) | 42 | (47.2%) | 27 | (57.4%) | 20 | (42.6%) |
| E14.5 | +/+ | +/+ | Sufficient | 35 | (97.2%) | 1 | (2.8%) | 35 | (100%) | 0 | (0%) |
|  | +/+ | +/+ | Limited | 33 | (46.5%) | 38 | (53.5%) | 31 | (93.9%) | 2 | (6.1%) |
|  | +/- | +/- | Sufficient | 48 | (88.9%) | 6 | (11.1%) | 48 | (100%) | 0 | (0%) |
|  | +/- | +/- | Limited | 44 | (57.9%) | 32 | (42.1%) | 31 | (70.5%) | 13 | (29.5%) |

E, embryonic day; M, maternal *Haao* genotype; P, paternal *Haao* genotype. For diet specifics, see Table S1.

^1^Malformed/Delayed category refers to developmental delay and/or exencephaly at E9.5, external structural anomalies at E11.5, and external structural anomalies and kidney anomalies (hypoplasia or agenesis) at E14.5. For a summary of the malformations observed at E11.5, see Table S3, and for those at E14.5, see Table S4. For individual litter outcome data, see Figure S1 and Tables S5, S6, S7.

**Table S3.** Summary of types and incidence of congenital malformations observed at E11.5.

| Malformation |  | | Sufficient Diet | Limited | Sufficient Diet | | | Limited Diet | | |
| --- | --- | --- | --- | --- | --- | --- | --- | --- | --- | --- |
|  |  |  |  | Diet |  |  |  |  |  |  |
|  | M | +/+ | | +/+ | +/- | | | +/- | | |
|  | E | +/+  n=34 | | +/+  n=38 | +/+  n=6 | +/-  n=18 | -/-  n=16 | +/+  n=12 | +/-  n=20 | -/-  n=15 |
| Exencephaly or open neural tube |  | 0  (0%) | | 0  (0%) | 0  (0%) | 0  (0%) | 0  (0%) | 1  (8%) | 4  (20%) | 0  (0%) |
| Underdeveloped or no eye(s) |  | 0  (0%) | | 1  (3%) | 0  (0%) | 0  (0%) | 0  (0%) | 1  (8%) | 4  (20%) | 1  (7%) |
| Underdeveloped limb(s) |  | 0  (0%) | | 0  (0%) | 0  (0%) | 0  (0%) | 0  (0%) | 1  (8%) | 3  (15%) | 5  (33%) |
| Kinks in spine |  | 0  (0%) | | 0  (0%) | 0  (0%) | 0  (0%) | 0  (0%) | 5  (42%) | 3  (15%) | 0  (0%) |
| Hooked tail |  | 0  (0%) | | 0  (0%) | 0  (0%) | 0  (0%) | 0  (0%) | 4  (33%) | 5  (25%) | 5  (33%) |
| Oedema |  | 0  (0%) | | 0  (0%) | 0  (0%) | 0  (0%) | 0  (0%) | 3  (25%) | 4  (20%) | 1  (7%) |
| TOTAL EMBRYO MALFORMED |  | 0  (0%) | | 1  (3%) | 0  (0%) | 0  (0%) | 0  (0%) | 7  (58%) | 8  (40%) | 5  (33%) |

M, maternal *Haao* genotype; E, embryonic *Haao* genotype. Paternal *Haao* genotype for the first two columns was *Haao*+/+, resulting in *Haao*+/+ embryos only. Paternal *Haao* genotype in the other columns was *Haao*+/-, resulting in embryos of all three *Haao* genotypes being generated. Percentages summarize malformation observed in live embryos only; dead embryos could not be phenotyped.

NB: Some embryos with underdeveloped eyes and limbs may also be attributed to developmental delay, as determined by embryonic weight measurements.

**Table S4.** Summary of types and incidence of congenital malformations observed at E14.5.

| Malformation |  | Sufficient Diet | Limited | Sufficient Diet | | | Limited Diet | | |
| --- | --- | --- | --- | --- | --- | --- | --- | --- | --- |
|  |  |  | Diet |  |  |  |  |  |  |
|  | M | +/+ | +/+ | +/- | | | +/- | | |
|  | E | +/+  n=36 | +/+  n=33 | +/+  n=15 | +/-  n=23 | -/-  n=12 | +/+  n=12 | +/-  n=26 | -/-  n=5 |
| Exencephaly |  | 0  (0%) | 1  (3%) | 0  (0%) | 0  (0%) | 0  (0%) | 0  (0%) | 3  (12%) | 0  (0%) |
| Underdeveloped or no eye(s) |  | 0  (0%) | 1  (3%) | 0  (0%) | 0  (0%) | 0  (0%) | 2  (17%) | 6  (23%) | 1  (20%) |
| *Anophthalmia* |  | *-* | *1*  *(3%)* | *-* | *-* | *-* | *2*  *(17%)* | *1*  *(4%)* | *1*  *(20%)* |
| *Microphthalmia or*  *coloboma* |  | *-* | *-* | *-* | *-* | *-* | *-* | *5*  *(19%)* | *-* |
| Limbs |  | 0  (0%) | 1  (3%) | 0  (0%) | 0  (0%) | 0  (0%) | 0  (0%) | 4  (15%) | 2  (40%) |
| *Underdeveloped*  *or short* |  | *-* | *1*  *(3%)* | *-* | *-* | *-* | *-* | *1*  *(4%)* | *1*  *(20%)* |
| *Talipes* |  | *-* | *-* | *-* | *-* | *-* | *-* | *3*  *(12%)* | *1*  *(20%)* |
| Digits |  | 0  (0%) | 1  (3%) | 0  (0%) | 0  (0%) | 0  (0%) | 2  (17%) | 9  (35%) | 1  (20%) |
| *Oligodactyly* |  | *-* | *1*  *(3%)* | *-* | *-* | *-* | *-* | *1*  *(4%)* | *1*  *(20%)* |
| *Syndactyly* |  | *-* | *-* | *-* | *-* | *-* | *-* | *1*  *(4%)* | *-* |
| *Polydactyly* |  | *-* | *-* | *-* | *-* | *-* | *2*  *(17%)* | *8*  *(31%)* | *-* |
| Lower jaw and palate |  | 0  (0%) | 0  (0%) | 0  (0%) | 0  (0%) | 0  (0%) | 0  (0%) | 1  (4%) | 2  (40%) |
| *Underdeveloped / partly missing* |  | *-* | *-* | *-* | *-* | *-* | *-* | *1*  *(4%)* | *2*  *(40%)* |
| *Cleft lip* |  | *-* | *-* | *-* | *-* | *-* | *-* | *-* | *-* |
| Caudal agenesis^1^ |  | 0  (0%) | 1  (3%) | 0  (0%) | 0  (0%) | 0  (0%) | 1  (8%) | 5  (19%) | 2  (40%) |
| Oedema |  | 0  (0%) | 2  (6%) | 0  (0%) | 0  (0%) | 0  (0%) | 1  (8%) | 7  (27%) | 2  (40%) |
| Kidney |  | 0  (0%) | 1  (3%) | 0  (0%) | 0  (0%) | 0  (0%) | 1  (8%) | 5  (19%) | 2  (40%) |
| *Agenesis* |  | *-* | *1*  *(3%)* | *-* | *-* | *-* | *1*  *(8%)* | *2*  *(8%)* | *1*  *(20%)* |
| *Hypoplasia^2^* |  | *-* | *-* | *-* | *-* | *-* |  | *5*  *(19%)* | *2*  *(40%)* |
| TOTAL MALFORMED |  | 0  (0%) | 2  (6%) | 0  (0%) | 0  (0%) | 0  (0%) | 2  (17%) | 9  (35%) | 2  (40%) |

M, maternal *Haao* genotype; E, embryonic *Haao* genotype. Paternal *Haao* genotype for the first two columns was *Haao*+/+, resulting in *Haao*+/+ embryos only. Paternal *Haao* genotype in the other columns was *Haao*+/-, resulting in embryos of all three *Haao* genotypes being generated. Percentages summarize malformations in live embryos only; dead embryos could not be phenotyped.

^1^Caudal agenesis refers to tails being bent backwards and/or curly in addition to being shortened.

^2^Average kidney length of E14.5 embryos on a breeder diet containing 90 mg/kg niacin and 3.7 g/kg L-tryptophan was 1.23 mm (n=11) whereas the average kidney length of E13.5 embryos on breeder diet was 0.932 mm (n=5). Five embryos across the dataset weighed less than the average for E13.5 on breeder diet (140.5 mg; n=5). To account for potential developmental delay in these embryos, kidney hypoplasia threshold was set for at least one kidney to be ≤0.7 mm.

NB: Some embryos with underdeveloped eyes and limbs may also be attributed to developmental delay, as determined by embryonic weight measurements.

**Table S5.** E9.5 embryo phenotyping details.

| Litter ID | Maternal genotype | Diet | Litter classification^1^ | Total implantations | | Embryo genotypes | | | Phenotype (live embryos) | | | Malformation types^2^ |
| --- | --- | --- | --- | --- | --- | --- | --- | --- | --- | --- | --- | --- |
|  |  |  |  | Live | Dead | *Haao*+/+ | *Haao*+/- | *Haao*-/- | Normal | Delayed | Malformed | Exencephaly |
| E9-NF-1 | WT | Sufficient | Normal | 6 | 0 | 6 | 0 | 0 | 6 | 0 | 0 | 0 |
| E9-NF-2 | WT | Sufficient | Normal | 7 | 0 | 7 | 0 | 0 | 7 | 0 | 0 | 0 |
| E9-NF-3 | WT | Sufficient | Normal | 6 | 0 | 6 | 0 | 0 | 6 | 0 | 0 | 0 |
| E9-NF-4 | WT | Sufficient | Normal | 5 | 1 | 5 | 0 | 0 | 5 | 0 | 0 | 0 |
| E9-NF-5 | WT | Sufficient | Normal | 7 | 0 | 7 | 0 | 0 | 7 | 0 | 0 | 0 |
| E9-TW600-1 | WT | Limited | Normal | 8 | 0 | 8 | 0 | 0 | 8 | 0 | 0 | 0 |
| E9-TW600-2 | WT | Limited | Normal | 7 | 1 | 7 | 0 | 0 | 7 | 0 | 0 | 0 |
| E9-TW600-3 | WT | Limited | Malformed | 8 | 0 | 8 | 0 | 0 | 7 | 0 | 1 | 1 |
| E9-TW600-4 | WT | Limited | Delayed | 8 | 0 | 8 | 0 | 0 | 7 | 1 | 0 | 0 |
| E9-TW600-5 | WT | Limited | Dead | 0 | 1 | 0 | 0 | 0 | 0 | 0 | 0 | 0 |
| E9-TW600-6 | WT | Limited | Dead | 0 | 6 | 0 | 0 | 0 | 0 | 0 | 0 | 0 |

^1^The ‘delayed’ group among E9.5 embryos was defined as embryos phenotypically E8.5 or younger.

WT, wild-type.

^2^Numbers indicate the number of embryos with the indicated malformation type.

**Table S6.** E11.5 embryo phenotyping details.

| Litter ID | Maternal genotype | Diet | Litter classification | Total implantation | | Embryo genotypes | | | Phenotype (live embryos) | | Malformation types^1^ | | | | | |
| --- | --- | --- | --- | --- | --- | --- | --- | --- | --- | --- | --- | --- | --- | --- | --- | --- |
|  |  |  |  | Live | Dead | *Haao*+/+ | *Haao*+/- | *Haao*-/- | Normal | Malformed | Exen-cephaly | Limbs | Kink in spine | Tail | Eye | Oedema |
| E11-WT-NF-1 | WT | Sufficient | Normal | 6 | 1 | 6 | 0 | 0 | 6 | 0 | 0 | 0 | 0 | 0 | 0 | 0 |
| E11-WT-NF-2 | WT | Sufficient | Normal | 7 | 0 | 7 | 0 | 0 | 7 | 0 | 0 | 0 | 0 | 0 | 0 | 0 |
| E11-WT-NF-3 | WT | Sufficient | Normal | 7 | 0 | 7 | 0 | 0 | 7 | 0 | 0 | 0 | 0 | 0 | 0 | 0 |
| E11-WT-NF-4 | WT | Sufficient | Normal | 8 | 0 | 8 | 0 | 0 | 8 | 0 | 0 | 0 | 0 | 0 | 0 | 0 |
| E11-WT-NF-5 | WT | Sufficient | Normal | 6 | 2 | 6 | 0 | 0 | 6 | 0 | 0 | 0 | 0 | 0 | 0 | 0 |
| E11-WT-TW600-1 | WT | Limited | Normal | 9 | 0 | 9 | 0 | 0 | 9 | 0 | 0 | 0 | 0 | 0 | 0 | 0 |
| E11-WT-TW600-2 | WT | Limited | Normal | 6 | 2 | 6 | 0 | 0 | 6 | 0 | 0 | 0 | 0 | 0 | 0 | 0 |
| E11-WT-TW600-3 | WT | Limited | Normal | 9 | 0 | 9 | 0 | 0 | 9 | 0 | 0 | 0 | 0 | 0 | 0 | 0 |
| E11-WT-TW600-4 | WT | Limited | Normal | 8 | 1 | 8 | 0 | 0 | 8 | 0 | 0 | 0 | 0 | 0 | 0 | 0 |
| E11-WT-TW600-5 | WT | Limited | Normal | 6 | 3 | 6 | 0 | 0 | 5 | 1 | 0 | 0 | 0 | 0 | 1 | 0 |
| E11-WT-TW600-6 | WT | Limited | Dead | 0 | 8 | 0 | 0 | 0 | 0 | 0 | 0 | 0 | 0 | 0 | 0 | 0 |
| E11-WT-TW600-7 | WT | Limited | Dead | 0 | 9 | 0 | 0 | 0 | 0 | 0 | 0 | 0 | 0 | 0 | 0 | 0 |
| E11-HH-NF-1 | *Haao*+/- | Sufficient | Normal | 9 | 0 | 2 | 4 | 3 | 9 | 0 | 0 | 0 | 0 | 0 | 0 | 0 |
| E11-HH-NF-2 | *Haao*+/- | Sufficient | Normal | 9 | 1 | 2 | 3 | 4 | 9 | 0 | 0 | 0 | 0 | 0 | 0 | 0 |
| E11-HH-NF-3 | *Haao*+/- | Sufficient | Normal | 8 | 0 | 0 | 5 | 3 | 8 | 0 | 0 | 0 | 0 | 0 | 0 | 0 |
| E11-HH-NF-4 | *Haao*+/- | Sufficient | Normal | 7 | 0 | 1 | 3 | 3 | 7 | 0 | 0 | 0 | 0 | 0 | 0 | 0 |
| E11-HH-NF-5 | *Haao*+/- | Sufficient | Normal | 7 | 1 | 1 | 3 | 3 | 7 | 0 | 0 | 0 | 0 | 0 | 0 | 0 |
| E11-HH-TW600-1 | *Haao*+/- | Limited | Normal | 9 | 1 | 2 | 2 | 5 | 9 | 0 | 0 | 0 | 0 | 0 | 0 | 0 |
| E11-HH-TW600-2 | *Haao*+/- | Limited | Normal | 5 | 3 | 2 | 2 | 1 | 5 | 0 | 0 | 0 | 0 | 0 | 0 | 0 |
| E11-HH-TW600-3 | *Haao*+/- | Limited | Normal | 9 | 0 | 0 | 5 | 4 | 9 | 0 | 0 | 0 | 0 | 0 | 0 | 0 |
| E11-HH-TW600-4 | *Haao*+/- | Limited | Malformed | 7 | 1 | 1 | 3 | 3 | 4 | 3 | 0 | 3 | 0 | 3 | 0 | 0 |
| E11-HH-TW600-5 | *Haao*+/- | Limited | Malformed | 2 | 5 | 0 | 2 | 0 | 0 | 2 | 2 | 2 | 0 | 1 | 0 | 0 |
| E11-HH-TW600-6 | *Haao*+/- | Limited | Malformed | 7 | 0 | 3 | 4 | 0 | 0 | 7 | 2 | 2 | 3 | 3 | 4 | 5 |
| E11-HH-TW600-7 | *Haao*+/- | Limited | Malformed | 8 | 1 | 4 | 2 | 2 | 0 | 8 | 1 | 2 | 5 | 7 | 2 | 3 |
| E11-HH-TW600-8 | *Haao*+/- | Limited | Dead | 0 | 3 | 0 | 0 | 0 | 0 | 0 | 0 | 0 | 0 | 0 | 0 | 0 |
| E11-HH-TW600-9 | *Haao*+/- | Limited | Dead | 0 | 4 | 0 | 0 | 0 | 0 | 0 | 0 | 0 | 0 | 0 | 0 | 0 |
| E11-HH-TW600-10 | *Haao*+/- | Limited | Dead | 0 | 9 | 0 | 0 | 0 | 0 | 0 | 0 | 0 | 0 | 0 | 0 | 0 |
| E11-HH-TW600-11 | *Haao*+/- | Limited | Dead | 0 | 7 | 0 | 0 | 0 | 0 | 0 | 0 | 0 | 0 | 0 | 0 | 0 |
| E11-HH-TW600-12 | *Haao*+/- | Limited | Dead | 0 | 8 | 0 | 0 | 0 | 0 | 0 | 0 | 0 | 0 | 0 | 0 | 0 |

WT, wild-type; *Haao*+/-, heterozygosity in the gene *Haao* which encodes 3-hydroxyanthranilate 3,4-dioxygenase.

^1^Numbers indicate the number of embryos with the indicated malformation type; embryos with multiple malformations are counted in all applicable malformation categories.

**Table S7.** E14.5 embryo phenotyping details.

| Litter ID | Maternal genotype | Diet | Litter classification | Total implantations | | Embryo genotypes | | | Phenotype (live embryos) | | Malformation types^1^ | | | | | | | |
| --- | --- | --- | --- | --- | --- | --- | --- | --- | --- | --- | --- | --- | --- | --- | --- | --- | --- | --- |
|  |  |  |  | Live | Dead | *Haao*+/+ | *Haao*+/- | *Haao*-/- | Normal | Malformed | Exen-cephaly | Limbs | Digits | Tail | Eye | Jaw / palate | Kidney | Oedema |
| E14-WT-NF-1 | WT | Sufficient | Normal | 6 | 1 | 6 | 0 | 0 | 6 | 0 | 0 | 0 | 0 | 0 | 0 | 0 | 0 | 0 |
| E14-WT-NF-2 | WT | Sufficient | Normal | 8 | 0 | 8 | 0 | 0 | 8 | 0 | 0 | 0 | 0 | 0 | 0 | 0 | 0 | 0 |
| E14-WT-NF-3 | WT | Sufficient | Normal | 7 | 0 | 7 | 0 | 0 | 7 | 0 | 0 | 0 | 0 | 0 | 0 | 0 | 0 | 0 |
| E14-WT-NF-4 | WT | Sufficient | Normal | 7 | 0 | 7 | 0 | 0 | 7 | 0 | 0 | 0 | 0 | 0 | 0 | 0 | 0 | 0 |
| E14-WT-NF-5 | WT | Sufficient | Normal | 7 | 0 | 7 | 0 | 0 | 7 | 0 | 0 | 0 | 0 | 0 | 0 | 0 | 0 | 0 |
| E14-WT-TW600-1 | WT | Limited | Normal | 7 | 2 | 7 | 0 | 0 | 7 | 0 | 0 | 0 | 0 | 0 | 0 | 0 | 0 | 0 |
| E14-WT-TW600-2 | WT | Limited | Normal | 8 | 0 | 8 | 0 | 0 | 8 | 0 | 0 | 0 | 0 | 0 | 0 | 0 | 0 | 0 |
| E14-WT-TW600-3 | WT | Limited | Normal | 9 | 0 | 9 | 0 | 0 | 9 | 0 | 0 | 0 | 0 | 0 | 0 | 0 | 0 | 0 |
| E14-WT-TW600-4 | WT | Limited | Normal | 8 | 0 | 8 | 0 | 0 | 7 | 1 | 0 | 0 | 0 | 0 | 0 | 0 | 0 | 1 |
| E14-WT-TW600-5 | WT | Limited | Malformed | 1 | 6 | 1 | 0 | 0 | 0 | 1 | 1 | 1 | 1 | 1 | 1 | 1 | 1 | 1 |
| E14-WT-TW600-6 | WT | Limited | Dead | 0 | 7 | 0 | 0 | 0 | 0 | 0 | 0 | 0 | 0 | 0 | 0 | 0 | 0 | 0 |
| E14-WT-TW600-7 | WT | Limited | Dead | 0 | 6 | 0 | 0 | 0 | 0 | 0 | 0 | 0 | 0 | 0 | 0 | 0 | 0 | 0 |
| E14-WT-TW600-8 | WT | Limited | Dead | 0 | 9 | 0 | 0 | 0 | 0 | 0 | 0 | 0 | 0 | 0 | 0 | 0 | 0 | 0 |
| E14-WT-TW600-9 | WT | Limited | Dead | 0 | 8 | 0 | 0 | 0 | 0 | 0 | 0 | 0 | 0 | 0 | 0 | 0 | 0 | 0 |
| E14-HH-NF-1 | *Haao*+/- | Sufficient | Normal | 8 | 0 | 4 | 4 | 0 | 8 | 0 | 0 | 0 | 0 | 0 | 0 | 0 | 0 | 0 |
| E14-HH-NF-2 | *Haao*+/- | Sufficient | Normal | 5 | 1 | 1 | 3 | 1 | 5 | 0 | 0 | 0 | 0 | 0 | 0 | 0 | 0 | 0 |
| E14-HH-NF-3 | *Haao*+/- | Sufficient | Normal | 6 | 2 | 1 | 2 | 3 | 6 | 0 | 0 | 0 | 0 | 0 | 0 | 0 | 0 | 0 |
| E14-HH-NF-4 | *Haao*+/- | Sufficient | Normal | 8 | 0 | 2 | 5 | 1 | 8 | 0 | 0 | 0 | 0 | 0 | 0 | 0 | 0 | 0 |
| E14-HH-NF-5 | *Haao*+/- | Sufficient | Normal | 7 | 1 | 2 | 4 | 1 | 7 | 0 | 0 | 0 | 0 | 0 | 0 | 0 | 0 | 0 |
| E14-HH-NF-6 | *Haao*+/- | Sufficient | Normal | 8 | 1 | 3 | 1 | 4 | 8 | 0 | 0 | 0 | 0 | 0 | 0 | 0 | 0 | 0 |
| E14-HH-NF-7 | *Haao*+/- | Sufficient | Normal | 6 | 1 | 1 | 4 | 1 | 6 | 0 | 0 | 0 | 0 | 0 | 0 | 0 | 0 | 0 |
| E14-HH-TW600-1 | *Haao*+/- | Limited | Normal | 7 | 1 | 3 | 4 | 0 | 7 | 0 | 0 | 0 | 0 | 0 | 0 | 0 | 0 | 0 |
| E14-HH-TW600-2 | *Haao*+/- | Limited | Normal | 8 | 0 | 3 | 5 | 0 | 8 | 0 | 0 | 0 | 0 | 0 | 0 | 0 | 0 | 0 |
| E14-HH-TW600-3 | *Haao*+/- | Limited | Normal | 8 | 0 | 1 | 4 | 3 | 8 | 0 | 0 | 0 | 0 | 0 | 0 | 0 | 0 | 0 |
| E14-HH-TW600-4 | *Haao*+/- | Limited | Malformed | 8 | 1 | 3 | 4 | 1 | 6 | 1 | 0 | 1 | 0 | 1 | 0 | 1 | 1 | 1 |
| E14-HH-TW600-5 | *Haao*+/- | Limited | Malformed | 6 | 1 | 1 | 4 | 1 | 0 | 6 | 0 | 1 | 6 | 1 | 3 | 2 | 1 | 3 |
| E14-HH-TW600-6 | *Haao*+/- | Limited | Malformed | 6 | 3 | 1 | 5 | 0 | 0 | 6 | 3 | 4 | 6 | 6 | 6 | 0 | 6 | 6 |
| E14-HH-TW600-7 | *Haao*+/- | Limited | Dead | 0 | 10 | 0 | 0 | 0 | 0 | 0 | 0 | 0 | 0 | 0 | 0 | 0 | 0 | 0 |
| E14-HH-TW600-8 | *Haao*+/- | Limited | Dead | 0 | 8 | 0 | 0 | 0 | 0 | 0 | 0 | 0 | 0 | 0 | 0 | 0 | 0 | 0 |
| E14-HH-TW600-9 | *Haao*+/- | Limited | Dead | 0 | 7 | 0 | 0 | 0 | 0 | 0 | 0 | 0 | 0 | 0 | 0 | 0 | 0 | 0 |
| E14-HH-TW600-10 | *Haao*+/- | Limited | Normal | 1 | 1 | 0 | 1 | 0 | 1 | 0 | 0 | 0 | 0 | 0 | 0 | 0 | 0 | 0 |

WT, wild-type; *Haao*+/-, heterozygosity in the gene *Haao* which encodes 3-hydroxyanthranilate 3,4-dioxygenase.

^1^Numbers indicate the number of embryos with the indicated malformation type; embryos with multiple malformations are counted in all applicable malformation categories.

**Table S8.** E9.5 maternal plasma NAD metabolome quantification.

| **Litter ID** | **Maternal Genotype** | **Diet** | **Litter Classification** | **TRP (µM)** | **KYN (µM)** | **3HK (nM)** | **3HAA (nM)** | **NAM (nM)** | **2PY (µM)** | **4PY (nM)** |
| --- | --- | --- | --- | --- | --- | --- | --- | --- | --- | --- |
| E9-NF-1 | WT | Sufficient | Normal | 91.1 | 3.83 | 63.0 | 45.9 | 282 | 10.1 | 9.80 |
| E9-NF-2 | WT | Sufficient | Normal | 76.0 | 3.37 | 75.4 | 38.6 | 283 | 11.3 | 11.8 |
| E9-NF-3 | WT | Sufficient | Normal | 94.1 | 3.92 | 54.5 | 58.4 | 601 | 58.1 | 58.1 |
| E9-NF-4 | WT | Sufficient | Normal | 63.2 | 3.93 | 53.1 | 51.1 | 460 | 12.2 | 10.8 |
| E9-NF-5 | WT | Sufficient | Normal | 75.7 | 1.79 | 38.8 | 21.9 | 886 | 167 | 150 |
| E9-TW600-1 | WT | Limited | Normal | 45.3 | 1.07 | 17.5 | 9.76 | 235 | <LOD | 2.73 |
| E9-TW600-2 | WT | Limited | Normal | 80.0 | 4.94 | 45.9 | 20.2 | 862 | 36.7 | 33.3 |
| E9-TW600-3 | WT | Limited | Malformed | 25.3 | 0.570 | 6.85 | <LOD | 400 | 3.28 | 3.23 |
| E9-TW600-4 | WT | Limited | Delayed | 46.9 | 1.86 | 19.8 | 13.8 | 239 | <LOD | 2.71 |
| E9-TW600-5 | WT | Limited | Dead | 57.7 | 0.667 | 14.4 | 17.9 | 173 | <LOD | 2.79 |
| E9-TW600-6 | WT | Limited | Dead | 27.7 | 0.329 | 7.14 | <LOD | 119 | <LOD | <LOD |

WT, wild-type; <LOD, below the limit of detection; TRP, L-tryptophan; KYN, kynurenine; 3HK, 3-hydroxykynurenine; 3HAA, 3-hydroxyanthranilic acid; NAM, nicotinamide; 2PY, N-methyl-2-pyridone-5-carboxamide; 4PY, N-methyl-4-pyridone-5-carboxamide.

**Table S9.** E11.5 maternal plasma NAD metabolome quantification.

| **Litter ID** | **Maternal genotype** | **Diet** | **Litter classification** | **TRP (µM)** | **KYN (µM)** | **3HK (nM)** | **3HAA (nM)** | **NAM (nM)** | **2PY (µM)** | **4PY (nM)** |
| --- | --- | --- | --- | --- | --- | --- | --- | --- | --- | --- |
| E11-WT-NF-1 | WT | Sufficient | Normal | 99.7 | 3.05 | 51.3 | 22.4 | 1085 | 203 | 206.9 |
| E11-WT-NF-2 | WT | Sufficient | Normal | 99.0 | 8.31 | 72.5 | 34.7 | 3047 | 384 | 218.1 |
| E11-WT-NF-3 | WT | Sufficient | Normal | 85.9 | 3.25 | 64.9 | 22.2 | 1238 | 65.8 | 65.9 |
| E11-WT-NF-4 | WT | Sufficient | Normal | 110 | 3.81 | 69.0 | 33.8 | 1477 | 418 | 422 |
| E11-WT-NF-5 | WT | Sufficient | Normal | 103 | 5.97 | 74.8 | 24.7 | 1952 | 394 | 412 |
| E11-WT-TW600-1 | WT | Limited | Normal | 69.6 | 2.99 | 53.1 | 17.9 | 1148 | 149 | 153 |
| E11-WT-TW600-2 | WT | Limited | Normal | 28.8 | 1.47 | 22.9 | 13.2 | 467 | 4.00 | 4.96 |
| E11-WT-TW600-3 | WT | Limited | Normal | 79.2 | 2.55 | 43.4 | 12.9 | 279 | 6.73 | 5.36 |
| E11-WT-TW600-4 | WT | Limited | Normal | 49.1 | 2.71 | 55.2 | 11.2 | 1244 | 66.8 | 69.1 |
| E11-WT-TW600-5 | WT | Limited | Normal | 54.7 | 1.13 | 25.4 | 5.62 | 1414 | 159 | 142 |
| E11-WT-TW600-6 | WT | Limited | Dead | 56.5 | 1.35 | 19.7 | <LOD | 299 | <LOD | 1.64 |
| E11-WT-TW600-7 | WT | Limited | Dead | 52.5 | 1.75 | 24.4 | 5.12 | 240 | 3.35 | <LOD |
| E11-HH-NF-1 | *Haao+/-* | Sufficient | Normal | 110 | 3.43 | 94.3 | 65.5 | 2387 | 537 | 526 |
| E11-HH-NF-2 | *Haao+/-* | Sufficient | Normal | 81.5 | 4.19 | 68.6 | 120 | 1410 | 88.2 | 105 |
| E11-HH-NF-3 | *Haao+/-* | Sufficient | Normal | 142 | 3.51 | 67.0 | 76.8 | 1939 | 420 | 399 |
| E11-HH-NF-4 | *Haao+/-* | Sufficient | Normal | 126 | 4.31 | 69.4 | 107 | 1982 | 151 | 155 |
| E11-HH-NF-5 | *Haao+/-* | Sufficient | Normal | 96.1 | 4.60 | 83.2 | 81.3 | 1694 | 532 | 523 |
| E11-HH-TW600-1 | *Haao+/-* | Limited | Normal | 57.5 | 3.53 | 88.4 | 54.4 | 1044 | 101 | 102 |
| E11-HH-TW600-2 | *Haao+/-* | Limited | Normal | 84.7 | 4.35 | 81.4 | 42.3 | 574 | 119 | 110 |
| E11-HH-TW600-3 | *Haao+/-* | Limited | Normal | 94.1 | 6.17 | 75.6 | 98.2 | 599 | 10.0 | 9.74 |
| E11-HH-TW600-4 | *Haao+/-* | Limited | Malformed | 19.9 | 0.809 | 15.2 | 29.8 | 699 | 5.66 | 6.23 |
| E11-HH-TW600-5 | *Haao+/-* | Limited | Malformed | 33.3 | 1.04 | 17.4 | 5.73 | 428 | <LOD | <LOD |
| E11-HH-TW600-6 | *Haao+/-* | Limited | Malformed | 64.4 | 1.18 | 27.5 | 7.43 | 1067 | 5.10 | 5.48 |
| E11-HH-TW600-7 | *Haao+/-* | Limited | Malformed | 42.5 | 1.38 | 24.1 | 17.0 | 437 | 4.53 | 3.99 |
| E11-HH-TW600-8 | *Haao+/-* | Limited | Dead | 94.4 | 2.22 | 38.2 | 24.8 | 288 | 4.29 | 3.66 |
| E11-HH-TW600-9 | *Haao+/-* | Limited | Dead | 43.1 | 0.695 | 13.0 | 4.83 | 442 | 3.75 | 4.35 |
| E11-HH-TW600-10 | *Haao+/-* | Limited | Dead | 63.6 | 3.19 | 54.9 | 27.9 | 556 | 7.43 | 7.38 |
| E11-HH-TW600-11 | *Haao+/-* | Limited | Dead | 37.4 | 1.46 | 25.1 | 7.59 | 301 | <LOD | 3.71 |
| E11-HH-TW600-12 | *Haao+/-* | Limited | Dead | 22.5 | 0.633 | 11.7 | <LOD | 180 | <LOD | 1.67 |

WT, wild-type; *Haao*+/-, heterozygosity in the gene *Haao* which encodes 3-hydroxyanthranilate 3,4-dioxygenase; <LOD, below the limit of detection; TRP, L-tryptophan; KYN, kynurenine; 3HK, 3-hydroxykynurenine; 3HAA, 3-hydroxyanthranilic acid; NAM, nicotinamide; 2PY, N-methyl-2-pyridone-5-carboxamide; 4PY, N-methyl-4-pyridone-5-carboxamide.

**Table S10.** E14.5 maternal plasma NAD metabolome quantification.

| **Litter ID** | **Maternal Genotype** | **Diet** | **Litter Classification** | **TRP (µM)** | **KYN (µM)** | **3HK (nM)** | **3HAA (nM)** | **NAM (nM)** | **2PY (nM)** | **4PY (nM)** |
| --- | --- | --- | --- | --- | --- | --- | --- | --- | --- | --- |
| E14-WT-NF-1 | WT | Sufficient | Normal | 89.3 | 4.17 | 72.4 | 18.1 | 705 | 225 | 260 |
| E14-WT-NF-2 | WT | Sufficient | Normal | 91.5 | 4.76 | 90.1 | 23.7 | 775 | 62.7 | 70 |
| E14-WT-NF-3 | WT | Sufficient | Normal | 79.2 | 2.70 | 74.1 | 18.2 | 490 | 85.8 | 81 |
| E14-WT-NF-4 | WT | Sufficient | Normal | 71.5 | 3.14 | 70.6 | 21.5 | 718 | 136 | 145 |
| E14-WT-NF-5 | WT | Sufficient | Normal | 76.7 | 3.60 | 71.3 | 19.8 | 1136 | 142 | 136 |
| E14-WT-TW600-1 | WT | Limited | Normal | 79.4 | 4.34 | 74.0 | 18.4 | 1106 | 247 | 264 |
| E14-WT-TW600-2 | WT | Limited | Normal | 68.7 | 4.38 | 79.1 | 17.0 | 2024 | 494 | 554 |
| E14-WT-TW600-3 | WT | Limited | Normal | 53.0 | 4.98 | 98.9 | 16.5 | 3486 | 633 | 626 |
| E14-WT-TW600-4 | WT | Limited | Normal | 77.1 | 3.25 | 74.0 | 26.6 | 678 | 134 | 138 |
| E14-WT-TW600-5 | WT | Limited | Malformed | 77.0 | 2.26 | 33.0 | 12.6 | 217 | 2.77 | 3.91 |
| E14-WT-TW600-6 | WT | Limited | Dead | 67.6 | 2.51 | 42.0 | 13.0 | 593 | 7.56 | 10.2 |
| E14-WT-TW600-7 | WT | Limited | Dead | 67.7 | 2.26 | 43.3 | 13.1 | 460 | 9.27 | 9.87 |
| E14-WT-TW600-8 | WT | Limited | Dead | 70.3 | 2.21 | 33.7 | 18.7 | 497 | 5.01 | 5.30 |
| E14-HH-NF-1 | *Haao+/-* | Sufficient | Normal | 72.3 | 3.23 | 69.2 | 27.8 | 665 | 155 | 143 |
| E14-HH-NF-2 | *Haao+/-* | Sufficient | Normal | 67.4 | 2.46 | 53.2 | 32.4 | 775 | 132 | 147 |
| E14-HH-NF-3 | *Haao+/-* | Sufficient | Normal | 73.6 | 3.22 | 83.3 | 72.7 | 567 | 170 | 191 |
| E14-HH-NF-4 | *Haao+/-* | Sufficient | Normal | 69.9 | 3.26 | 66.0 | 35.4 | 572 | 81.8 | 91.5 |
| E14-HH-NF-5 | *Haao+/-* | Sufficient | Normal | 72.4 | 3.68 | 64.9 | 35.1 | 924 | 249 | 278 |
| E14-HH-NF-6 | *Haao+/-* | Sufficient | Normal | 66.5 | 3.77 | 77.8 | 70.2 | 512 | 103 | 116 |
| E14-HH-TW600-1 | *Haao+/-* | Limited | Normal | 63.1 | 3.79 | 89.5 | 30.6 | 1174 | 192 | 230 |
| E14-HH-TW600-2 | *Haao+/-* | Limited | Normal | 48.7 | 3.03 | 76.2 | 11.8 | 1590 | 159 | 163 |
| E14-HH-TW600-3 | *Haao+/-* | Limited | Normal | 65.8 | 2.13 | 39.0 | 27.6 | 1056 | 112 | 128 |
| E14-HH-TW600-4 | *Haao+/-* | Limited | Malformed | 54.7 | 2.77 | 54.0 | 28.3 | 948 | 125 | 140 |
| E14-HH-TW600-5 | *Haao+/-* | Limited | Malformed | 71.2 | 3.94 | 91.4 | 37.4 | 942 | 175 | 229 |
| E14-HH-TW600-6 | *Haao+/-* | Limited | Malformed | 42.5 | 1.79 | 29.6 | 8.65 | 756 | 52.2 | 65.2 |
| E14-HH-TW600-7 | *Haao+/-* | Limited | Dead | 58.2 | 1.81 | 34.4 | 15.4 | 296 | 9.64 | 11.9 |
| E14-HH-TW600-8 | *Haao+/-* | Limited | Dead | 29.3 | 0.899 | 17.4 | 3.43 | 772 | 11.9 | 11.1 |
| E14-HH-TW600-9 | *Haao+/-* | Limited | Dead | 55.8 | 1.35 | 32.7 | 10.9 | 440 | 11.0 | 11.8 |

WT, wild-type; *Haao*+/-, heterozygosity in the gene *Haao* which encodes 3-hydroxyanthranilate 3,4-dioxygenase; TRP, L-tryptophan; KYN, kynurenine; 3HK, 3-hydroxykynurenine; 3HAA, 3-hydroxyanthranilic acid; NAM, nicotinamide; 2PY, N-methyl-2-pyridone-5-carboxamide; 4PY, N-methyl-4-pyridone-5-carboxamide.

**Table S11.** Summary of embryo NAD(H) concentrations at E9.5, E11.5 and E14.5.

|  |  | Genotype | | | NAD(H) (nmol/mg protein ± SD) |  |  |
| --- | --- | --- | --- | --- | --- | --- | --- |
| Stage | Diet | Maternal | Paternal | Embryo |  | *n* | *p* |
| E9.5 | Sufficient | +/+ | +/+ | +/+ | 7.97 ± 1.55 | 15 |  |
|  | Limited | +/+ | +/+ | +/+ | 4.02 ± 3.01 | 12 | 0.0002 |
| E11.5 | Sufficient | +/+ | +/+ | +/+ | 3.05 ± 0.72 | 15 |  |
|  | Limited | +/+ | +/+ | +/+ | 1.97 ± 0.97 | 11 | 0.0111 |
|  | Sufficient | +/- | +/- | +/+ | 2.79 ± 0.83 | 6 | 0.9991 |
|  |  |  |  | +/- | 3.24 ± 0.32 | 10 | 0.9997 |
|  |  |  |  | -/- | 3.18 ± 0.70 | 10 | >0.9999 |
|  | Limited | +/- | +/- | +/+ | 1.80 ± 0.82 | 10 | 0.0084 |
|  |  |  |  | +/- | 1.68 ± 0.92 | 13 | 0.0008 |
|  |  |  |  | -/- | 1.22 ± 0.77 | 10 | <0.0001 |
| E14.5 | Sufficient | +/+ | +/+ | +/+ | 11.0 ± 1.37 | 15 |  |
|  | Limited | +/+ | +/+ | +/+ | 9.86 ± 2.79 | 13 | 0.8970 |
|  | Sufficient | +/- | +/- | +/+ | 10.4 ± 1.90 | 10 | 0.9989 |
|  |  |  |  | +/- | 11.1 ± 0.99 | 10 | >0.9999 |
|  |  |  |  | -/- | 10.7 ± 1.87 | 10 | >0.9999 |
|  | Limited | +/- | +/- | +/+ | 11.5 ± 1.22 | 10 | 0.9998 |
|  |  |  |  | +/- | 11.1 ± 0.88 | 12 | >0.9999 |
|  |  |  |  | -/- | 9.68 ± 262 | 5 | 0.9583 |

*p* values were calculated by one-way ANOVA with Tukey’s multiple comparisons test. The *p* values comparing the indicated treatment groups with the maternal wild-type on Sufficient Diet control group of the respective embryonic stage are shown. The *p* values comparing *Haao*+/+, *Haao*+/-, and *Haao*-/- embryos within the same diet condition are all >0.05, indicating no significant difference in NAD levels between embryo genotypes at E11.5 and E14.5. NAD(H), sum of NAD^+^ and NADH.

**Table S12.** E9.5 embryo NAD(H) concentrations.

| **Litter ID** | **Embryo ID** | **Embryo genotype** | **Maternal diet** | **Number of somites** | **NAD(H) (nmol / mg protein)** | **Average NAD(H) per litter** |
| --- | --- | --- | --- | --- | --- | --- |
| E9-NF-1 | E9-NF-1-a | WT | Sufficient | 17 | 5.94 | 5.81 |
|  | E9-NF-1-b | WT | Sufficient | 18 | 6.08 |  |
|  | E9-NF-1-c | WT | Sufficient | 19 | 5.39 |  |
| E9-NF-2 | E9-NF-2-a | WT | Sufficient | 16 | 9.28 | 7.98 |
|  | E9-NF-2-b | WT | Sufficient | 18 | 7.73 |  |
|  | E9-NF-2-c | WT | Sufficient | 17 | 6.95 |  |
| E9-NF-3 | E9-NF-3-a | WT | Sufficient | 19 | 8.92 | 8.91 |
|  | E9-NF-3-b | WT | Sufficient | 16 | 10.0 |  |
|  | E9-NF-3-c | WT | Sufficient | 19 | 7.80 |  |
| E9-NF-4 | E9-NF-4-a | WT | Sufficient | 14 | 6.34 | 8.00 |
|  | E9-NF-4-b | WT | Sufficient | 16 | 8.69 |  |
|  | E9-NF-4-c | WT | Sufficient | 15 | 8.99 |  |
| E9-NF-5 | E9-NF-5-a | WT | Sufficient | 13 | 7.79 | 9.15 |
|  | E9-NF-5-b | WT | Sufficient | 14 | 9.32 |  |
|  | E9-NF-5-c | WT | Sufficient | 17 | 10.3 |  |
| E9-TW600-1 | E9-TW600-1-a | WT | Limited | 22 | 1.83 | 1.77 |
|  | E9-TW600-1-b | WT | Limited | 21 | 1.62 |  |
|  | E9-TW600-1-c | WT | Limited | 19 | 1.87 |  |
| E9-TW600-2 | E9-TW600-2-a | WT | Limited | 19 | 7.45 | 8.52 |
|  | E9-TW600-2-b | WT | Limited | 22 | 9.41 |  |
|  | E9-TW600-2-c | WT | Limited | 22 | 8.70 |  |
| E9-TW600-3 | E9-TW600-3-a | WT | Limited | 14 | 3.76 | 4.22 |
|  | E9-TW600-3-b | WT | Limited | 17 | 3.27 |  |
|  | E9-TW600-3-c | WT | Limited | 18 | 5.64 |  |
| E9-TW600-4 | E9-TW600-4-a | WT | Limited | 20 | 1.62 | 1.55 |
|  | E9-TW600-4-b | WT | Limited | 17 | 1.75 |  |
|  | E9-TW600-4-c | WT | Limited | 21 | 1.26 |  |

WT, wild-type; NAD(H), sum of NAD^+^ and NADH.

**Table S13.** E11.5 embryo NAD(H) concentrations.

| **Litter ID** | **Embryo ID** | **Embryo genotype** | **Maternal diet** | **Embryo Phenotype** | **Embryo weight (mg)** | **NAD(H) (nmol / mg protein)** | **Average NAD(H) per litter** |
| --- | --- | --- | --- | --- | --- | --- | --- |
| E11-WT-NF-1 | E11-WT-NF-1-a | WT | Sufficient | Normal | 43.2 | 3.37 | 2.59 |
|  | E11-WT-NF-1-b | WT | Sufficient | Normal | 43.5 | 1.98 |  |
|  | E11-WT-NF-1-c | WT | Sufficient | Normal | 32.1 | 2.41 |  |
| E11-WT-NF-2 | E11-WT-NF-2-a | WT | Sufficient | Normal | 37.9 | 3.64 | 3.39 |
|  | E11-WT-NF-2-b | WT | Sufficient | Normal | 36.3 | 3.34 |  |
|  | E11-WT-NF-2-c | WT | Sufficient | Normal | 42.5 | 3.19 |  |
| E11-WT-NF-3 | E11-WT-NF-3-a | WT | Sufficient | Normal | 42.7 | 2.45 | 2.51 |
|  | E11-WT-NF-3-b | WT | Sufficient | Normal | 44.3 | 2.84 |  |
|  | E11-WT-NF-3-c | WT | Sufficient | Normal | 46.5 | 2.23 |  |
| E11-WT-NF-4 | E11-WT-NF-4-a | WT | Sufficient | Normal | 30.7 | 2.90 | 3.38 |
|  | E11-WT-NF-4-b | WT | Sufficient | Normal | 31.2 | 4.16 |  |
|  | E11-WT-NF-4-c | WT | Sufficient | Normal | 38.3 | 3.08 |  |
| E11-WT-NF-5 | E11-WT-NF-5-a | WT | Sufficient | Normal | 43.8 | 4.66 | 3.38 |
|  | E11-WT-NF-5-b | WT | Sufficient | Normal | 41.3 | 2.50 |  |
|  | E11-WT-NF-5-c | WT | Sufficient | Normal | 45.6 | 2.98 |  |
| E11-WT-TW600-1 | E11-WT-TW600-1-a | WT | Limited | Normal | 45.7 | 4.06 | 2.40 |
|  | E11-WT-TW600-1-b | WT | Limited | Normal | 45.3 | 1.52 |  |
|  | E11-WT-TW600-1-c | WT | Limited | Normal | 46.8 | 1.63 |  |
| E11-WT-TW600-2 | E11-WT-TW600-2-a | WT | Limited | Normal | 34.7 | 1.52 | 1.52 |
|  | E11-WT-TW600-2-b | WT | Limited | Normal | 33 | 1.82 |  |
|  | E11-WT-TW600-2-c | WT | Limited | Normal | 34 | 1.22 |  |
| E11-WT-TW600-3 | E11-WT-TW600-3-a | WT | Limited | Normal | 36.2 | 1.08 | 0.88 |
|  | E11-WT-TW600-3-b | WT | Limited | Normal | 30.9 | 0.79 |  |
|  | E11-WT-TW600-3-c | WT | Limited | Normal | 36.6 | 0.78 |  |
| E11-WT-TW600-4 | E11-WT-TW600-4-a | WT | Limited | Normal | 59.3 | 1.66 | 1.69 |
|  | E11-WT-TW600-4-b | WT | Limited | Normal | 54 | 1.67 |  |
|  | E11-WT-TW600-4-c | WT | Limited | Normal | 55 | 1.74 |  |
| E11-WT-TW600-5 | E11-WT-TW600-5-a | WT | Limited | Normal | 37.9 | 3.46 | 3.02 |
|  | E11-WT-TW600-5-b | WT | Limited | Normal | 36.2 | 3.29 |  |
|  | E11-WT-TW600-5-c | WT | Limited | Delayed | 15.1 | 2.66 |  |
|  | E11-WT-TW600-5-d | WT | Limited | Normal | 28.8 | 2.68 |  |
| E11-HH-NF-1 | E11-HH-NF-1-a | *Haao+/+* | Sufficient | Normal | 46.8 | 2.74 | 3.08 |
|  | E11-HH-NF-1-b | *Haao+/-* | Sufficient | Normal | 45.3 | 2.95 |  |
|  | E11-HH-NF-1-c | *Haao-/-* | Sufficient | Normal | 40.2 | 3.48 |  |
|  | E11-HH-NF-1-d | *Haao-/-* | Sufficient | Normal | 51.2 | 3.70 |  |
|  | E11-HH-NF-1-e | *Haao+/-* | Sufficient | Normal | 52.3 | 3.46 |  |
|  | E11-HH-NF-1-f | *Haao+/+* | Sufficient | Normal | 52.6 | 2.17 |  |
| E11-HH-NF-2 | E11-HH-NF-2-a | *Haao+/+* | Sufficient | Normal | 39.3 | 2.58 | 2.84 |
|  | E11-HH-NF-2-b | *Haao+/-* | Sufficient | Normal | 39.9 | 2.61 |  |
|  | E11-HH-NF-2-c | *Haao-/-* | Sufficient | Normal | 52.7 | 1.61 |  |
|  | E11-HH-NF-2-d | *Haao-/-* | Sufficient | Normal | 39.9 | 2.64 |  |
|  | E11-HH-NF-2-e | *Haao+/-* | Sufficient | Normal | 43.7 | 3.21 |  |
|  | E11-HH-NF-2-f | *Haao+/+* | Sufficient | Normal | 47.3 | 4.40 |  |
| E11-HH-NF-3 | E11-HH-NF-3-a | *Haao+/-* | Sufficient | Normal | 48.9 | 3.07 | 3.18 |
|  | E11-HH-NF-3-b | *Haao-/-* | Sufficient | Normal | 41.9 | 2.78 |  |
|  | E11-HH-NF-3-c | *Haao-/-* | Sufficient | Normal | 43.6 | 3.72 |  |
|  | E11-HH-NF-3-d | *Haao+/-* | Sufficient | Normal | 47.9 | 3.15 |  |
| E11-HH-NF-4 | E11-HH-NF-4-a | *Haao+/+* | Sufficient | Normal | 42.4 | 2.13 | 3.25 |
|  | E11-HH-NF-4-b | *Haao+/-* | Sufficient | Normal | 34.6 | 3.28 |  |
|  | E11-HH-NF-4-c | *Haao-/-* | Sufficient | Normal | 24.6 | 4.08 |  |
|  | E11-HH-NF-4-d | *Haao-/-* | Sufficient | Normal | 35.1 | 3.33 |  |
|  | E11-HH-NF-4-e | *Haao+/-* | Sufficient | Normal | 33.2 | 3.33 |  |
| E11-HH-NF-5 | E11-HH-NF-5-a | *Haao+/-* | Sufficient | Normal | 35.3 | 3.50 | 3.26 |
|  | E11-HH-NF-5-b | *Haao-/-* | Sufficient | Normal | 38.2 | 3.21 |  |
|  | E11-HH-NF-5-c | *Haao-/-* | Sufficient | Normal | 42.2 | 3.11 |  |
|  | E11-HH-NF-5-d | *Haao+/-* | Sufficient | Normal | 41.3 | 3.79 |  |
|  | E11-HH-NF-5-e | *Haao+/+* | Sufficient | Normal | 40.5 | 2.70 |  |
| E11-HH-TW600-1 | E11-HH-TW600-1-a | *Haao+/+* | Limited | Normal | 41.7 | 3.40 | 3.14 |
|  | E11-HH-TW600-1-b | *Haao+/-* | Limited | Normal | 44.2 | 3.06 |  |
|  | E11-HH-TW600-1-c | *Haao-/-* | Limited | Normal | 36.6 | 2.00 |  |
|  | E11-HH-TW600-1-d | *Haao-/-* | Limited | Normal | 34.8 | 3.06 |  |
|  | E11-HH-TW600-1-e | *Haao+/-* | Limited | Normal | 41.6 | 4.17 |  |
|  | E11-HH-TW600-1-f | *Haao+/+* | Limited | Normal | 36.3 | 3.15 |  |
| E11-HH-TW600-2 | E11-HH-TW600-2-a | *Haao+/+* | Limited | Normal | 45.6 | 1.72 | 1.33 |
|  | E11-HH-TW600-2-b | *Haao+/-* | Limited | Normal | 51.2 | 1.50 |  |
|  | E11-HH-TW600-2-c | *Haao-/-* | Limited | Normal | 60.1 | 0.71 |  |
|  | E11-HH-TW600-2-d | *Haao+/-* | Limited | Normal | 57.3 | 1.27 |  |
|  | E11-HH-TW600-2-e | *Haao+/+* | Limited | Normal | 53.4 | 1.45 |  |
| E11-HH-TW600-3 | E11-HH-TW600-3-a | *Haao-/-* | Limited | Normal | 34.3 | 0.77 | 0.90 |
|  | E11-HH-TW600-3-b | *Haao-/-* | Limited | Normal | 30.6 | 0.70 |  |
|  | E11-HH-TW600-3-c | *Haao+/-* | Limited | Normal | 32.2 | 0.98 |  |
|  | E11-HH-TW600-3-d | *Haao+/-* | Limited | Normal | 40.1 | 1.14 |  |
| E11-HH-TW600-4 | E11-HH-TW600-4-a | *Haao+/+* | Limited | Normal | 26.8 | 1.65 | 1.24 |
|  | E11-HH-TW600-4-b | *Haao+/-* | Limited | Normal | 29.2 | 1.22 |  |
|  | E11-HH-TW600-4-c | *Haao-/-* | Limited | Malformed | 22.2 | 1.20 |  |
|  | E11-HH-TW600-4-d | *Haao-/-* | Limited | Malformed | 20.1 | 0.64 |  |
|  | E11-HH-TW600-4-e | *Haao-/-* | Limited | Malformed | 24.9 | 1.28 |  |
|  | E11-HH-TW600-4-f | *Haao+/-* | Limited | Normal | 28.4 | 1.46 |  |
| E11-HH-TW600-6 | E11-HH-TW600-6-a | *Haao+/+* | Limited | Malformed | 23.8 | 1.12 | 1.30 |
|  | E11-HH-TW600-6-b | *Haao+/-* | Limited | Malformed | 22.5 | 1.36 |  |
|  | E11-HH-TW600-6-c | *Haao+/-* | Limited | Malformed | 23.6 | 1.08 |  |
|  | E11-HH-TW600-6-d | *Haao+/-* | Limited | Malformed | 28.8 | 1.27 |  |
|  | E11-HH-TW600-6-e | *Haao+/+* | Limited | Malformed | 28 | 1.77 |  |
|  | E11-HH-TW600-6-f | *Haao+/+* | Limited | Malformed | 33.8 | 1.20 |  |
| E11-HH-TW600-7 | E11-HH-TW600-7-a | *Haao+/+* | Limited | Malformed | 27.4 | 1.46 | 1.29 |
|  | E11-HH-TW600-7-b | *Haao+/-* | Limited | Malformed | 19.1 | 1.95 |  |
|  | E11-HH-TW600-7-c | *Haao-/-* | Limited | Malformed | 19.7 | 1.02 |  |
|  | E11-HH-TW600-7-d | *Haao+/+* | Limited | Malformed | 26.5 | 1.06 |  |
|  | E11-HH-TW600-7-e | *Haao-/-* | Limited | Malformed | 18.6 | 0.83 |  |
|  | E11-HH-TW600-7-f | *Haao+/-* | Limited | Malformed | 22.8 | 1.42 |  |

WT, wild-type; *Haao*+/+, embryo from a *Haao*+/- x *Haao*+/- intercross with two wild-type alleles in the gene *Haao* which encodes 3-hydroxyanthranilate 3,4-dioxygenase; *Haao*+/-, embryo with heterozygosity in *Haao*; *Haao*-/-, embryo with two loss-of-function alleles in *Haao*; NAD(H), sum of NAD^+^ and NADH.

**Table S14.** E14.5 embryo NAD(H) concentrations.

| **Litter ID** | **Embryo ID** | **Embryo genotype** | **Maternal diet** | **Embryo Phenotype** | **Embryo weight (mg)** | **NAD(H) (nmol / mg protein)** | **Average NAD(H) per litter** |
| --- | --- | --- | --- | --- | --- | --- | --- |
| E14-WT-NF-1 | E14-WT-NF-1-a | WT | Sufficient | Normal | 240.1 | 9.97 | 10.4 |
|  | E14-WT-NF-1-b | WT | Sufficient | Normal | 216.3 | 9.82 |  |
|  | E14-WT-NF-1-c | WT | Sufficient | Normal | 227.1 | 11.5 |  |
| E14-WT-NF-2 | E14-WT-NF-2-a | WT | Sufficient | Normal | 240.4 | 11.0 | 11.5 |
|  | E14-WT-NF-2-b | WT | Sufficient | Normal | 199.1 | 11.0 |  |
|  | E14-WT-NF-2-c | WT | Sufficient | Normal | 241.8 | 12.4 |  |
| E14-WT-NF-3 | E14-WT-NF-3-a | WT | Sufficient | Normal | 243.4 | 11.6 | 10.6 |
|  | E14-WT-NF-3-b | WT | Sufficient | Normal | 235.0 | 8.04 |  |
|  | E14-WT-NF-3-c | WT | Sufficient | Normal | 242.4 | 12.1 |  |
| E14-WT-NF-4 | E14-WT-NF-4-a | WT | Sufficient | Normal | 232.3 | 12.1 | 12.4 |
|  | E14-WT-NF-4-b | WT | Sufficient | Normal | 222.1 | 11.7 |  |
|  | E14-WT-NF-4-c | WT | Sufficient | Normal | 209.7 | 13.2 |  |
| E14-WT-NF-5 | E14-WT-NF-5-a | WT | Sufficient | Normal | 215.7 | 10.8 | 10.2 |
|  | E14-WT-NF-5-b | WT | Sufficient | Normal | 213.6 | 11.0 |  |
|  | E14-WT-NF-5-c | WT | Sufficient | Normal | 202.4 | 8.85 |  |
| E14-WT-TW600-1 | E14-WT-TW600-1-a | WT | Limited | Normal | 213.8 | 11.2 | 11.2 |
|  | E14-WT-TW600-1-b | WT | Limited | Normal | 222.8 | 10.3 |  |
|  | E14-WT-TW600-1-c | WT | Limited | Normal | 238.8 | 12.2 |  |
| E14-WT-TW600-2 | E14-WT-TW600-2-a | WT | Limited | Normal | 236.3 | 10.1 | 8.95 |
|  | E14-WT-TW600-2-b | WT | Limited | Normal | 228.0 | 9.66 |  |
|  | E14-WT-TW600-2-c | WT | Limited | Normal | 250.8 | 7.11 |  |
| E14-WT-TW600-3 | E14-WT-TW600-3-a | WT | Limited | Normal | 228.2 | 13.2 | 10.9 |
|  | E14-WT-TW600-3-b | WT | Limited | Normal | 231.8 | 10.5 |  |
|  | E14-WT-TW600-3-c | WT | Limited | Normal | 239.7 | 8.97 |  |
| E14-WT-TW600-4 | E14-WT-TW600-4-a | WT | Limited | Normal | 259.6 | 10.8 | 11.0 |
|  | E14-WT-TW600-4-b | WT | Limited | Normal | 237.1 | 11.2 |  |
|  | E14-WT-TW600-4-c | WT | Limited | Malformed | 251.4 | 10.9 |  |
| E14-WT-TW600-5 | E14-WT-TW600-5-a | WT | Limited | Malformed | 100.0 | 2.00 | 2.00 |
| E14-HH-NF-1 | E14-HH-NF-1-a | *Haao+/-* | Sufficient | Normal | 231.1 | 10.8 | 11.5 |
|  | E14-HH-NF-1-b | *Haao+/-* | Sufficient | Normal | 233.6 | 10.9 |  |
|  | E14-HH-NF-1-c | *Haao+/+* | Sufficient | Normal | 234.3 | 11.4 |  |
|  | E14-HH-NF-1-d | *Haao+/+* | Sufficient | Normal | 203.4 | 11.9 |  |
|  | E14-HH-NF-1-e | *Haao+/+* | Sufficient | Normal | 212.8 | 12.6 |  |
| E14-HH-NF-2 | E14-HH-NF-2-a | *Haao-/-* | Sufficient | Normal | 224.4 | 13.4 | 12.9 |
|  | E14-HH-NF-2-b | *Haao+/-* | Sufficient | Normal | 253.8 | 12.0 |  |
|  | E14-HH-NF-2-c | *Haao+/-* | Sufficient | Normal | 237.2 | 12.7 |  |
|  | E14-HH-NF-2-d | *Haao+/+* | Sufficient | Normal | 258.8 | 13.5 |  |
| E14-HH-NF-3 | E14-HH-NF-3-a | *Haao-/-* | Sufficient | Normal | 269.7 | 11.0 | 10.7 |
|  | E14-HH-NF-3-b | *Haao-/-* | Sufficient | Normal | 263.4 | 10.6 |  |
|  | E14-HH-NF-3-c | *Haao-/-* | Sufficient | Normal | 271.3 | 12.5 |  |
|  | E14-HH-NF-3-d | *Haao+/-* | Sufficient | Normal | 299.8 | 10.4 |  |
|  | E14-HH-NF-3-e | *Haao+/+* | Sufficient | Normal | 260.6 | 8.84 |  |
| E14-HH-NF-4 | E14-HH-NF-4-a | *Haao-/-* | Sufficient | Normal | 203.8 | 10.1 | 9.51 |
|  | E14-HH-NF-4-b | *Haao+/-* | Sufficient | Normal | 224.0 | 9.99 |  |
|  | E14-HH-NF-4-c | *Haao+/-* | Sufficient | Normal | 252.7 | 9.84 |  |
|  | E14-HH-NF-4-d | *Haao+/+* | Sufficient | Normal | 248.2 | 8.75 |  |
|  | E14-HH-NF-4-e | *Haao+/+* | Sufficient | Normal | 237.9 | 8.90 |  |
| E14-HH-NF-5 | E14-HH-NF-5-a | *Haao-/-* | Sufficient | Normal | 215.1 | 6.87 | 10.2 |
|  | E14-HH-NF-5-b | *Haao+/-* | Sufficient | Normal | 219.6 | 11.2 |  |
|  | E14-HH-NF-5-c | *Haao+/-* | Sufficient | Normal | 217.1 | 12.4 |  |
|  | E14-HH-NF-5-d | *Haao+/+* | Sufficient | Normal | 225.1 | 10.3 |  |
|  | E14-HH-NF-5-e | *Haao+/+* | Sufficient | Normal | 186.4 | 10.2 |  |
| E14-HH-NF-6 | E14-HH-NF-6-a | *Haao-/-* | Sufficient | Normal | 224.6 | 11.4 | 10.2 |
|  | E14-HH-NF-6-b | *Haao-/-* | Sufficient | Normal | 233.6 | 9.70 |  |
|  | E14-HH-NF-6-c | *Haao-/-* | Sufficient | Normal | 195.4 | 12.3 |  |
|  | E14-HH-NF-6-d | *Haao-/-* | Sufficient | Normal | 229.0 | 9.35 |  |
|  | E14-HH-NF-6-e | *Haao+/-* | Sufficient | Normal | 211.2 | 10.8 |  |
|  | E14-HH-NF-6-f | *Haao+/+* | Sufficient | Normal | 240.2 | 7.69 |  |
| E14-HH-TW600-1 | E14-HH-TW600-1-a | *Haao+/-* | Limited | Normal | 247.9 | 10.9 | 11.5 |
|  | E14-HH-TW600-1-b | *Haao+/+* | Limited | Normal | 243 | 12.1 |  |
|  | E14-HH-TW600-1-c | *Haao+/+* | Limited | Normal | 244.1 | 12.4 |  |
|  | E14-HH-TW600-1-d | *Haao+/+* | Limited | Normal | 237.1 | 10.6 |  |
| E14-HH-TW600-2 | E14-HH-TW600-2-a | *Haao+/-* | Limited | Normal | 200.9 | 12.4 | 11.8 |
|  | E14-HH-TW600-2-b | *Haao+/-* | Limited | Normal | 208.2 | 12.0 |  |
|  | E14-HH-TW600-2-c | *Haao+/-* | Limited | Normal | 209.6 | 11.6 |  |
|  | E14-HH-TW600-2-d | *Haao+/+* | Limited | Normal | 193.8 | 12.6 |  |
|  | E14-HH-TW600-2-e | *Haao+/+* | Limited | Normal | 206.2 | 10.4 |  |
| E14-HH-TW600-3 | E14-HH-TW600-3-a | *Haao-/-* | Limited | Normal | 228.8 | 13.4 | 11.2 |
|  | E14-HH-TW600-3-b | *Haao-/-* | Limited | Normal | 240.2 | 9.75 |  |
|  | E14-HH-TW600-3-c | *Haao-/-* | Limited | Normal | 245.7 | 9.36 |  |
|  | E14-HH-TW600-3-d | *Haao+/-* | Limited | Normal | 245.2 | 10.7 |  |
|  | E14-HH-TW600-3-e | *Haao+/+* | Limited | Normal | 285.0 | 12.9 |  |
| E14-HH-TW600-4 | E14-HH-TW600-4-a | *Haao-/-* | Limited | Malformed | 159.3 | 9.91 | 11.2 |
|  | E14-HH-TW600-4-b | *Haao+/-* | Limited | Normal | 210.1 | 11.3 |  |
|  | E14-HH-TW600-4-c | *Haao+/+* | Limited | Normal | 228.8 | 12.9 |  |
|  | E14-HH-TW600-4-d | *Haao+/+* | Limited | Normal | 183.2 | 10.5 |  |
| E14-HH-TW600-5 | E14-HH-TW600-5-a | *Haao-/-* | Limited | Malformed | 108.5 | 5.99 | 9.76 |
|  | E14-HH-TW600-5-b | *Haao+/-* | Limited | Malformed | 175.4 | 11.5 |  |
|  | E14-HH-TW600-5-c | *Haao+/-* | Limited | Malformed | 202.4 | 10.1 |  |
|  | E14-HH-TW600-5-d | *Haao+/-* | Limited | Malformed | 213.5 | 10.7 |  |
|  | E14-HH-TW600-5-e | *Haao+/+* | Limited | Malformed | 166.7 | 10.6 |  |
| E14-HH-TW600-6 | E14-HH-TW600-6-a | *Haao+/-* | Limited | Malformed | 231.1 | 9.41 | 10.4 |
|  | E14-HH-TW600-6-b | *Haao+/-* | Limited | Malformed | 150.3 | 11.9 |  |
|  | E14-HH-TW600-6-c | *Haao+/-* | Limited | Malformed | 166.4 | 10.3 |  |
|  | E14-HH-TW600-6-d | *Haao+/+* | Limited | Malformed | 187.2 | 9.75 |  |

WT, wild-type; *Haao*+/+, embryo from a *Haao*+/- x *Haao*+/- intercross with two wild-type alleles in the gene *Haao* which encodes 3-hydroxyanthranilate 3,4-dioxygenase; *Haao*+/-, embryo with heterozygosity in *Haao*; *Haao*-/-, embryo with two loss-of-function alleles in *Haao*; NAD(H), sum of NAD^+^ and NADH.

**Table S15.** Concentrations of NAD^+^ and related metabolites in E9.5 yolk sacs.

| **Litter ID** | **Maternal genotype** | **Maternal diet** | **Litter classification** | **TRP  (nmol / mg protein)** | **KYN  (pmol / mg protein)** | **3HK  (pmol / mg protein)** | **3HAA  (pmol / mg protein)** | **QA  (pmol / mg protein)** | **NAMN  (pmol / mg protein)** | **NAD^+^  (nmol / mg protein)** | **NAM  (pmol / mg protein)** | **NMN  (pmol / mg protein)** |
| --- | --- | --- | --- | --- | --- | --- | --- | --- | --- | --- | --- | --- |
| E9-NF-1 | WT | Sufficient | Normal | 2.30 | 285 | 42.7 | <LOD | 169 | 4.90 | 3.78 | 869 | 12.1 |
| E9-NF-2 | WT | Sufficient | Normal | 1.58 | 139 | 17.3 | 7.02 | 67.6 | 2.10 | 4.65 | 324 | 6.84 |
| E9-NF-3 | WT | Sufficient | Normal | 1.97 | 110 | 18.9 | <LOD | 67.0 | 1.57 | 4.20 | 268 | 7.75 |
| E9-NF-4 | WT | Sufficient | Normal | 1.46 | 208 | 21.9 | <LOD | 69.1 | 2.47 | 3.79 | 433 | 9.25 |
| E9-NF-5 | WT | Sufficient | Normal | 1.54 | 83.7 | 17.7 | <LOD | 85.9 | 2.25 | 3.89 | 812 | 7.59 |
| E9-TW600-1 | WT | Limited | Normal | 1.37 | 27.9 | <LOD | <LOD | 37.3 | 0.471 | 2.47 | 294 | 7.99 |
| E9-TW600-2 | WT | Limited | Normal | 2.06 | 179 | 16.5 | 3.73 | 114 | 2.21 | 4.19 | 493 | 7.83 |
| E9-TW600-3 | WT | Limited | Malformed | 2.33 | 33.3 | 3.50 | <LOD | 21.9 | 0.309 | 2.19 | 205 | 4.47 |
| E9-TW600-4 | WT | Limited | Delayed | 1.03 | 20.1 | 4.14 | <LOD | 28.2 | 0.653 | 2.98 | 370 | 4.96 |

WT, wild-type; <LOD, below the limit of detection; TRP, L-tryptophan; KYN, kynurenine; 3HK, 3-hydroxykynurenine; 3HAA, 3-hydroxyanthranilic acid; QA, quinolinic acid; NaMN, nicotinic acid mononucleotide; NAM, nicotinamide; NMN, nicotinamide mononucleotide.

The metabolites N-methyl-2-pyridone-5-carboxamide (2PY), N-methyl-4-pyridone-5-carboxamide (4PY), and kynurenic acid (KA) were below the detection limit in all samples.

**Table S16.** Summary of HAAO enzyme activity measured in yolk sacs collected at E11.5.

|  |  | Genotype | | | HAAO activity /mg protein ± SD^1^ |  |  |
| --- | --- | --- | --- | --- | --- | --- | --- |
| Stage | Diet | Maternal | Paternal | Embryo |  | *n* | *p* |
| E11.5 | Sufficient | +/+ | +/+ | +/+ | 16.16 ± 4.78 | 10 |  |
|  | Limited | +/+ | +/+ | +/+ | 16.14 ± 3.42 | 10 | >0.9999 |
|  | Sufficient | +/- | +/- | +/+ | 15.05 ± 1.57 | 3 | 0.9996 |
|  |  |  |  | +/- | 8.28 ± 1.42 | 5 | 0.0029 |
|  |  |  |  | -/- | 1.03 ± 0.67 | 5 | <0.0001 |
|  | Limited | +/- | +/- | +/+ | 15.79 ± 4.60 | 4 | >0.9999 |
|  |  |  |  | +/- | 8.69 ± 2.53 | 5 | 0.0054 |
|  |  |  |  | -/- | 1.21 ± 1.02 | 3 | <0.0001 |

*p* values were calculated by one-way ANOVA followed by Tukey’s multiple comparisons test. The *p* values comparing the indicated groups with the maternal wild-type on Sufficient Diet control group are shown.

^1^Values represent average HAAO activity (µmol/min/mg protein) ± standard deviation.

**Table S17.** HAAO enzyme activity in E11.5 yolk sacs.

| **Litter ID** | **Embryo ID** | **Embryo genotype** | **Maternal diet** | **Embryo phenotype** | **Embryo weight (mg)** | **HAAO activity (µmol/ min/ mg protein)** | **Average HAAO activity / genotype and diet^1^** |
| --- | --- | --- | --- | --- | --- | --- | --- |
| E11-WT-NF-1 | E11-WT-NF-1-b | WT | Sufficient | Normal | 43.5 | 16.25 | 16.16 |
| E11-WT-NF-1 | E11-WT-NF-1-d | WT | Sufficient | Normal | 43.2 | 15.60 |  |
| E11-WT-NF-2 | E11-WT-NF-2-c | WT | Sufficient | Normal | 42.5 | 27.79 |  |
| E11-WT-NF-2 | E11-WT-NF-2-d | WT | Sufficient | Normal | 43.8 | 15.66 |  |
| E11-WT-NF-3 | E11-WT-NF-3-c | WT | Sufficient | Normal | 46.5 | 14.46 |  |
| E11-WT-NF-3 | E11-WT-NF-3-d | WT | Sufficient | Normal | 45.0 | 11.73 |  |
| E11-WT-NF-4 | E11-WT-NF-4-b | WT | Sufficient | Normal | 31.2 | 12.30 |  |
| E11-WT-NF-4 | E11-WT-NF-4-d | WT | Sufficient | Normal | 30.8 | 17.54 |  |
| E11-WT-NF-5 | E11-WT-NF-5-c | WT | Sufficient | Normal | 45.6 | 18.90 |  |
| E11-WT-NF-5 | E11-WT-NF-5-d | WT | Sufficient | Normal | 44.5 | 11.34 |  |
| E11-WT-TW600-1 | E11-WT-TW600-1-c | WT | Limited | Normal | 46.8 | 16.90 | 16.14 |
| E11-WT-TW600-1 | E11-WT-TW600-1-d | WT | Limited | Normal | 46.1 | 17.64 |  |
| E11-WT-TW600-2 | E11-WT-TW600-2-b | WT | Limited | Normal | 33.0 | 15.89 |  |
| E11-WT-TW600-2 | E11-WT-TW600-2-c | WT | Limited | Normal | 34.0 | 17.39 |  |
| E11-WT-TW600-3 | E11-WT-TW600-3-c | WT | Limited | Normal | 36.6 | 14.64 |  |
| E11-WT-TW600-3 | E11-WT-TW600-3-d | WT | Limited | Normal | 35.7 | 12.12 |  |
| E11-WT-TW600-4 | E11-WT-TW600-4-b | WT | Limited | Normal | 54.0 | 16.47 |  |
| E11-WT-TW600-4 | E11-WT-TW600-4-c | WT | Limited | Normal | 55.0 | 13.21 |  |
| E11-WT-TW600-5 | E11-WT-TW600-5-b | WT | Limited | Normal | 36.2 | 24.12 |  |
| E11-WT-TW600-5 | E11-WT-TW600-5-e | WT | Limited | Normal | 39.7 | 12.98 |  |
| E11-HH-NF-1 | E11-HH-NF-1-f | *Haao+/+* | Sufficient | Normal | 52.6 | 13.61 | 15.05 |
| E11-HH-NF-2 | E11-HH-NF-2-f | *Haao+/+* | Sufficient | Normal | 47.3 | 16.73 |  |
| E11-HH-NF-5 | E11-HH-NF-5-e | *Haao+/+* | Sufficient | Normal | 40.5 | 14.80 |  |
| E11-HH-NF-1 | E11-HH-NF-1-e | *Haao+/-* | Sufficient | Normal | 52.3 | 7.161 | 8.284 |
| E11-HH-NF-2 | E11-HH-NF-2-e | *Haao+/-* | Sufficient | Normal | 43.7 | 9.844 |  |
| E11-HH-NF-3 | E11-HH-NF-3-d | *Haao+/-* | Sufficient | Normal | 47.9 | 9.416 |  |
| E11-HH-NF-4 | E11-HH-NF-4-e | *Haao+/-* | Sufficient | Normal | 33.2 | 8.477 |  |
| E11-HH-NF-5 | E11-HH-NF-5-d | *Haao+/-* | Sufficient | Normal | 41.3 | 6.521 |  |
| E11-HH-NF-1 | E11-HH-NF-1-d | *Haao-/-* | Sufficient | Normal | 51.2 | 1.227 | 1.034 |
| E11-HH-NF-2 | E11-HH-NF-2-d | *Haao-/-* | Sufficient | Normal | 39.9 | 1.072 |  |
| E11-HH-NF-3 | E11-HH-NF-3-c | *Haao-/-* | Sufficient | Normal | 43.6 | 2.028 |  |
| E11-HH-NF-4 | E11-HH-NF-4-d | *Haao-/-* | Sufficient | Normal | 35.1 | 0.3487 |  |
| E11-HH-NF-5 | E11-HH-NF-5-c | *Haao-/-* | Sufficient | Normal | 42.2 | 0.4934 |  |
| E11-HH-TW600-1 | E11-HH-TW600-1-f | *Haao+/+* | Limited | Normal | 36.3 | 10.38 | 15.79 |
| E11-HH-TW600-2 | E11-HH-TW600-2-e | *Haao+/+* | Limited | Normal | 53.4 | 20.76 |  |
| E11-HH-TW600-6 | E11-HH-TW600-6-e | *Haao+/+* | Limited | Malformed | 28.0 | 13.85 |  |
| E11-HH-TW600-7 | E11-HH-TW600-7-g | *Haao+/+* | Limited | Malformed | 26.8 | 18.17 |  |
| E11-HH-TW600-1 | E11-HH-TW600-1-e | *Haao+/-* | Limited | Normal | 41.6 | 8.151 | 8.689 |
| E11-HH-TW600-2 | E11-HH-TW600-2-d | *Haao+/-* | Limited | Normal | 57.3 | 6.033 |  |
| E11-HH-TW600-4 | E11-HH-TW600-4-f | *Haao+/-* | Limited | Normal | 28.4 | 12.77 |  |
| E11-HH-TW600-6 | E11-HH-TW600-6-d | *Haao+/-* | Limited | Malformed | 28.8 | 9.020 |  |
| E11-HH-TW600-7 | E11-HH-TW600-7-f | *Haao+/-* | Limited | Malformed | 22.8 | 7.471 |  |
| E11-HH-TW600-1 | E11-HH-TW600-1-d | *Haao-/-* | Limited | Normal | 34.8 | 2.380 | 1.213 |
| E11-HH-TW600-4 | E11-HH-TW600-4-e | *Haao-/-* | Limited | Malformed | 24.9 | 0.5194 |  |
| E11-HH-TW600-7 | E11-HH-TW600-7-e | *Haao-/-* | Limited | Malformed | 18.6 | 0.7387 |  |

WT, wild-type; *Haao*+/+, embryo from a *Haao*+/- x *Haao*+/- intercross with two wild-type alleles in the gene *Haao* which encodes 3-hydroxyanthranilate 3,4-dioxygenase; *Haao*+/-, embryo with heterozygosity in *Haao*; *Haao*-/-, embryo with two loss-of-function alleles in *Haao.*

^1^Average HAAO activity of each embryo *Haao* genotype and maternal diet combination.

**Table S18.** Concentrations of NAD^+^ and related metabolites in E11.5 yolk sacs.

| **Litter ID** | **Embryo ID** | **Maternal genotype** | **Embryo phenotype** | **Embryo weight (mg)** | **Embryo NAD(H)** | **TRP** | **KYN** | **3HK** | **3HAA** | **NAMN** | **NAD^+^** | **NAM** | **NMN** | **1MNA** | **2PY** | **4PY** | **KA** |
| --- | --- | --- | --- | --- | --- | --- | --- | --- | --- | --- | --- | --- | --- | --- | --- | --- | --- |
| E11-HH-NF-2 | E11-HH-NF-2-c | *Haao-/-* | Normal | 52.7 | 1.61 | 137 | 33.4 | 2461 | 11.5 | 44.4 | 72.0 | 50.6 | 3.57 | 479 | 90.9 | 114 | 818 |
| E11-HH-NF-5 | E11-HH-NF-5-b | *Haao-/-* | Normal | 38.2 | 3.21 | 117 | 20.6 | 2226 | 11.9 | 34.0 | 81.9 | 41.9 | 2.66 | <LOD | 255 | 227 | 520 |
| E11-HH-NF-1 | E11-HH-NF-1-c | *Haao-/-* | Normal | 40.2 | 3.48 | 104 | 33.2 | 3523 | 12.8 | 26.0 | 72.5 | 25.9 | 3.08 | 594 | 263 | 270 | 538 |
| E11-HH-NF-3 | E11-HH-NF-3-b | *Haao-/-* | Normal | 41.9 | 2.78 | 130 | 30.7 | 3699 | 15.7 | 24.8 | 66.9 | 42.1 | 3.35 | 459 | 200 | 204 | 566 |
| E11-HH-NF-4 | E11-HH-NF-4-c | *Haao-/-* | Normal | 24.6 | 4.08 | 102 | 30.1 | 2637 | 19.6 | 29.1 | 59.8 | 41.0 | 2.70 | <LOD | 117 | 98.5 | 538 |
| E11-HH-TW600-4 | E11-HH-TW600-4-c | *Haao-/-* | Malformed | 22.2 | 1.20 | 31.4 | 5.23 | 188 | 5.16 | 11.4 | 29.2 | 17.2 | 1.38 | <LOD | <LOD | <LOD | 204 |
| E11-HH-TW600-7 | E11-HH-TW600-7-c | *Haao-/-* | Malformed | 19.7 | 1.02 | 67.6 | 15.4 | 315 | 10.4 | 15.9 | 26.6 | 17.6 | 1.55 | <LOD | <LOD | <LOD | 212 |
| E11-HH-TW600-4 | E11-HH-TW600-4-d | *Haao-/-* | Malformed (kinked tail) | 20.1 | 0.64 | 30.8 | 4.72 | 322 | 4.49 | <LOQ | 32.3 | 7.70 | 0.194 | 308 | <LOD | <LOD | 234 |
| E11-HH-TW600-3 | E11-HH-TW600-3-a | *Haao-/-* | Normal | 34.3 | 0.77 | 175 | 24.5 | 2411 | 14.4 | <LOQ | 60.7 | 14.4 | 0.725 | <LOD | <LOD | <LOD | 552 |
| E11-HH-TW600-2 | E11-HH-TW600-2-c | *Haao-/-* | Normal | 60.1 | 0.71 | 140 | 27.3 | 704 | 15.4 | 60.4 | 62.3 | 44.6 | 7.42 | <LOD | 73.1 | 80.9 | 498 |
| E11-HH-TW600-1 | E11-HH-TW600-1-c | *Haao-/-* | Normal | 36.6 | 2.00 | 108 | 22.1 | 2512 | 17.0 | 62.2 | 56.1 | 39.8 | 2.14 | <LOD | <LOD | 49.7 | 463 |
| E11-HH-NF-3 | E11-HH-NF-3-a | *Haao+/-* | Normal | 48.9 | 3.07 | 123 | 35.0 | 1263 | 0.110 | 867 | 82.3 | 39.7 | 4.29 | 576 | 236 | 232 | 920 |
| E11-HH-NF-1 | E11-HH-NF-1-b | *Haao+/-* | Normal | 45.3 | 2.95 | 123 | 36.5 | 567 | 0.167 | 1027 | 88.2 | 28.4 | 2.53 | 684 | 198 | 230 | 1295 |
| E11-HH-NF-2 | E11-HH-NF-2-b | *Haao+/-* | Normal | 39.9 | 2.61 | 117 | 35.7 | 1185 | <LOD | 732 | 93.0 | 38.1 | 2.56 | 825 | 109 | 87.1 | 612 |
| E11-HH-NF-4 | E11-HH-NF-4-b | *Haao+/-* | Normal | 34.6 | 3.28 | 93.6 | 27.1 | 396 | <LOD | 858 | 59.2 | 31.4 | 2.86 | 250 | 60.3 | 57.8 | 327 |
| E11-HH-NF-5 | E11-HH-NF-5-a | *Haao+/-* | Normal | 35.3 | 3.50 | 159 | 27.4 | 778 | <LOD | 969 | 91.9 | 47.5 | 4.13 | 440 | 282 | 293 | 580 |
| E11-HH-TW600-6 | E11-HH-TW600-6-c | *Haao+/-* | Malformed | 23.6 | 1.08 | 101 | 18.6 | 431 | 0.238 | 1105 | 92.6 | 47.6 | 0.920 | <LOD | <LOD | <LOD | 491 |
| E11-HH-TW600-6 | E11-HH-TW600-6-b | *Haao+/-* | Malformed | 22.5 | 1.36 | 90.3 | 14.6 | 292 | <LOD | 1080 | 64.2 | 40.4 | 2.94 | 227 | <LOD | <LOD | 217 |
| E11-HH-TW600-7 | E11-HH-TW600-7-b | *Haao+/-* | Malformed | 19.1 | 1.95 | 70.2 | 11.3 | 333 | <LOD | 917 | 85.3 | 41.2 | 5.56 | <LOD | <LOD | <LOD | 187 |
| E11-HH-TW600-2 | E11-HH-TW600-2-b | *Haao+/-* | Normal | 51.2 | 1.50 | 114 | 33.1 | 500 | <LOD | 1255 | 102 | 50.9 | 8.47 | <LOD | 50.4 | 72.4 | 608 |
| E11-HH-TW600-1 | E11-HH-TW600-1-b | *Haao+/-* | Normal | 44.2 | 3.06 | 90.4 | 24.8 | 473 | <LOD | 973 | 81.9 | 44.4 | 4.80 | 475 | 79.9 | 56.5 | 740 |
| E11-HH-TW600-4 | E11-HH-TW600-4-b | *Haao+/-* | Normal | 29.2 | 1.22 | 18.4 | <LOD | 82 | <LOD | 137 | 43.4 | 14.7 | 1.98 | <LOD | <LOD | <LOD | 76.5 |
| E11-HH-NF-1 | E11-HH-NF-1-a | *Haao+/+* | Normal | 46.8 | 2.74 | 113 | 23.9 | 668 | <LOD | 828 | 61.7 | 30.3 | 3.28 | <LOD | <LOD | <LOD | 1229 |
| E11-HH-NF-2 | E11-HH-NF-2-a | *Haao+/+* | Normal | 39.3 | 2.58 | 121 | 34.6 | 530 | <LOD | 958 | 73.6 | 49.6 | 3.11 | 453 | 80.2 | 62.1 | 445 |
| E11-HH-NF-4 | E11-HH-NF-4-a | *Haao+/+* | Normal | 42.4 | 2.13 | 116 | 27.3 | 507 | <LOD | 894 | 75.9 | 41.5 | 3.98 | <LOD | 134 | 116 | 580 |
| E11-HH-TW600-7 | E11-HH-TW600-7-d | *Haao+/+* | Malformed | 26.5 | 1.06 | 109 | 12.6 | 487 | 0.150 | 1250 | 111 | 33.8 | 6.27 | 222 | <LOD | <LOD | 439 |
| E11-HH-TW600-6 | E11-HH-TW600-6-a | *Haao+/+* | Malformed | 23.8 | 1.12 | 90.4 | 16.7 | 359 | <LOD | 711 | 69.5 | 41.0 | 5.43 | <LOD | <LOD | <LOD | 318 |
| E11-HH-TW600-7 | E11-HH-TW600-7-a | *Haao+/+* | Malformed | 27.4 | 1.46 | 74.1 | 12.5 | 318 | <LOD | 813 | 74.6 | 52.4 | 6.19 | <LOD | <LOD | <LOD | 185 |
| E11-HH-TW600-2 | E11-HH-TW600-2-a | *Haao+/+* | Normal | 45.6 | 1.72 | 120 | 20.0 | 523 | <LOD | 1124 | 88.2 | 61.2 | 8.40 | <LOD | 91.0 | 69.6 | 375 |
| E11-HH-TW600-1 | E11-HH-TW600-1-a | *Haao+/+* | Normal | 41.7 | 3.40 | 89.9 | 21.8 | 435 | <LOD | 923 | 68.6 | 25.0 | 5.30 | <LOD | 53.3 | 52.4 | 383 |
| E11-HH-TW600-4 | E11-HH-TW600-4-a | *Haao+/+* | Normal | 26.8 | 1.65 | 44.4 | 4.43 | 124 | <LOD | 252 | 55.5 | 19.3 | 2.24 | <LOD | 189 | 54.5 | 148 |
| E11-WT-NF-3 | E11-WT-NF-3-b | WT | Normal | 44.3 | 2.84 | 173 | 37.6 | 709 | 0.205 | 1413 | 93.2 | 30.7 | 1.82 | 442 | 43.4 | 59.6 | 671 |
| E11-WT-NF-1 | E11-WT-NF-1-a | WT | Normal | 43.2 | 3.37 | 111 | 24.9 | 300 | <LOD | 893 | 86.1 | 23.6 | 3.84 | 320 | 83.3 | 77.7 | 619 |
| E11-WT-NF-2 | E11-WT-NF-2-a | WT | Normal | 37.9 | 3.64 | 149 | 54.2 | 953 | <LOD | 1495 | 84.7 | 43.0 | 3.73 | 474 | 183 | 198 | 587 |
| E11-WT-NF-3 | E11-WT-NF-3-a | WT | Normal | 42.7 | 2.45 | 147 | 27.5 | 539 | <LOD | 654 | 94.8 | 27.1 | 4.88 | <LOD | 105 | 73.4 | 763 |
| E11-WT-NF-4 | E11-WT-NF-4-a | WT | Normal | 30.7 | 2.90 | 126 | 28.4 | 684 | <LOD | 1431 | 98.4 | 52.9 | 4.17 | 495 | 252 | 231 | 598 |
| E11-WT-NF-5 | E11-WT-NF-5-a | WT | Normal | 43.8 | 4.66 | 184 | 35.1 | 1085 | <LOD | 1197 | 98.4 | 36.1 | 3.32 | 563 | 237 | 240 | 867 |
| E11-WT-TW600-1 | E11-WT-TW600-1-b | WT | Normal | 45.3 | 1.52 | 152 | 29.6 | 1613 | 0.520 | 1092 | 97.6 | 20.4 | 2.45 | 434 | 81.3 | 111 | 885 |
| E11-WT-TW600-1 | E11-WT-TW600-1-a | WT | Normal | 45.7 | 4.06 | 106 | 18.2 | 954 | <LOD | 879 | 76.1 | 34.4 | 4.69 | <LOD | 106 | 86.9 | 700 |
| E11-WT-TW600-2 | E11-WT-TW600-2-a | WT | Normal | 34.7 | 1.52 | 62.8 | 7.84 | 238 | <LOD | 378 | 59.2 | 28.2 | 2.89 | <LOD | <LOD | <LOD | 262 |
| E11-WT-TW600-3 | E11-WT-TW600-3-a | WT | Normal | 36.2 | 1.08 | 114 | 19.4 | 310 | <LOD | 846 | 70.9 | 31.4 | 4.17 | <LOD | <LOD | <LOD | 399 |
| E11-WT-TW600-4 | E11-WT-TW600-4-a | WT | Normal | 59.3 | 1.66 | 123 | 29.0 | 299 | <LOD | 802 | 73.0 | 31.7 | 4.06 | 432 | <LOD | 43.7 | 580 |
| E11-WT-TW600-5 | E11-WT-TW600-5-a | WT | Normal | 37.9 | 3.46 | 101 | 21.0 | 584 | <LOD | 957 | 85.4 | 41.8 | 3.75 | <LOD | 78.4 | 72.0 | 289 |

Embryo NAD(H) is in nmol/mg protein, all other metabolite concentrations are in pmol/ mg protein.

WT, wild-type; *Haao*+/-, heterozygosity in the gene *Haao* which encodes 3-hydroxyanthranilate 3,4-dioxygenase; TRP, L-tryptophan; KYN, kynurenine; 3HK, 3-hydroxykynurenine; 3HAA, 3-hydroxyanthranilic acid; QA, quinolinic acid; NaMN, nicotinic acid mononucleotide; NAM, nicotinamide; NMN, nicotinamide mononucleotide; 1MNA, 1-methylnicotinamide; 2PY, N-methyl-2-pyridone-5-carboxamide; 4PY, N-methyl-4-pyridone-5-carboxamide; KA, kynurenic acid.

NB: 1MNA was not included in analyses shown in Figure 4A, B.

**SI References**

1 Cuny, H. *et al.* NAD deficiency due to environmental factors or gene-environment interactions causes congenital malformations and miscarriage in mice. *Proc Natl Acad Sci U S A* **117**, 3738-3747 (2020). <https://doi.org:10.1073/pnas.1916588117>

2 Cuny, H. *et al.* Maternal heterozygosity of Slc6a19 causes metabolic perturbation and congenital NAD deficiency disorder in mice. *Dis Model Mech* **16**, dmm049647 (2023). <https://doi.org:10.1242/dmm.049647>

3 Szot, J. O. *et al.* A metabolic signature for NADSYN1-dependent congenital NAD deficiency disorder. *J Clin Invest* **134**, e174824 (2024). <https://doi.org:10.1172/JCI174824>

4 Bozon, K. *et al.* Impaired yolk sac NAD metabolism disrupts murine embryogenesis with relevance to human birth defects. *eLife* **13**, RP97649 (2024). <https://doi.org:10.7554/elife.97649.1>

5 Bachmanov, A. A., Reed, D. R., Beauchamp, G. K. & Tordoff, M. G. Food intake, water intake, and drinking spout side preference of 28 mouse strains. *Behav Genet* **32**, 435-443 (2002). <https://doi.org:10.1023/a:1020884312053>

6 Goldsmith, G. A. Niacin-tryptophan relationships in man and niacin requirement. *Am J Clin Nutr* **6**, 479-486 (1958). <https://doi.org:10.1093/ajcn/6.5.479>
